# Retarding moisture-induced chemical degradation of Yttrium Tellurides by tailoring grain boundary chemistry

**DOI:** 10.1038/s41467-026-76056-8

**Published:** 2026-07-25

**Authors:** Kyuseon Jang, Jamil Ur Rahman, Su-Hyun Yoo, Chanwon Jung, Eric Woods, Ruben Bueno-Villoro, Kornelius Nielsch, Christina Scheu, Yonghyuk Lee, Pyuck-Pa Choi, Ran He, Siyuan Zhang

**Affiliations:** 1 Department of Materials Science and Engineering, Korea Advanced Institute of Science and Technology (KAIST), Daejeon, Republic of Korea; 2 Max Planck Institute for Sustainable Materials, Düsseldorf, Germany; 3 Thermoelectric Materials and Devices, IFW Dresden, Dresden, Germany; 4 Digital Chemical Research Center, Korea Research Institute of Chemical Technology, Daejeon, Republic of Korea; 5 Department of Materials Science and Engineering, Pukyong National University, Busan, Republic of Korea; 6 Department of Chemical and Biochemical Engineering, Dongguk University, Seoul, Republic of Korea

**Keywords:** Thermoelectrics, Thermoelectric devices and materials

## Abstract

Grain boundary engineering has been extensively applied to improve thermoelectric performance, but its potential to enhance chemical stability remains underexplored. Here, we demonstrate that modifying grain boundary chemistry can effectively suppress the chemical degradation of Y_2_Te_3_ under ambient conditions. Scanning transmission electron microscopy and atom probe tomography reveal that H_2_O preferentially infiltrates along grain boundaries, initiating oxidation of Y_2_Te_3_ into Y–O–H phases and causing chemo-mechanical breakdown of the matrix. This process, remarkably, can be retarded by just 1 at.% of Bi incorporation due to its segregation along grain boundaries. Density functional theory calculations reveal the thermodynamic and kinetic origins of Bi segregation, and show how segregated Bi modifies the local electronic and chemical environment of grain boundaries, thereby linking GB chemistry to both chemical stability and thermoelectric performance. These findings establish multifunctional grain boundary engineering as a generalizable strategy for the design of next-generation thermoelectric materials.

## Abstract (web summary)

Y_2_Te_3_ thermoelectrics degrade in moist air as water molecules enter along grain boundaries. The authors show that 1 at.% Bi segregates to these boundaries, suppressing moisture ingress and preventing chemical degradation while preserving performance.

## Introduction

The ongoing global energy crisis underscores the need for sustainable energy conversion technologies^1^. Among various options, thermoelectric (TE) technology, which directly converts waste heat into electricity, has garnered considerable interest^2^. The scalability and mechanical reliability of TE materials make them particularly appealing for waste heat recovery and active cooling applications^3^. For practical application, however, TE materials must combine high energy conversion efficiency with long-term stability^4^. Although substantial progress has been made in improving the dimensionless figure-of-merit (zT), stability remains a major technical bottleneck for real-world applications^5^.

Thermal and chemical stability are both essential for reliable TE operation, but they represent distinct challenges. Thermal stability describes the retention of TE performance during thermal cycling or prolonged high-temperature operation, whereas chemical stability refers to resistance against reactive environments such as air, moisture, or residual contaminants^6^. TE materials inevitably encounter oxygen and moisture during service, fabrication steps (e.g., high-energy ball milling^7^, sintering^8^) and module assembly^9^. Nevertheless, most stability studies have focused on thermal stability under a vacuum or an inert atmosphere^10–12^, while chemical degradation under reactive environments remains less understood.

Recent studies have examined chemical degradation in several TE compounds, including Zintl compounds^6,13^, tellurides^14^, oxides^15,16^, and skutterudites^17^. Surface coatings, such as boron nitride spray or atomic layer deposition, have been explored as protective strategies^9,18^. However, additional coatings increase processing complexity and may introduce interfacial reactions during high-temperature operation^19^. An attractive alternative is to intrinsically design the microstructure so that the material resists chemical attack without sacrificing TE transport properties.

Grain boundary (GB) engineering offers a promising route toward this goal. In nanostructured TE materials, GBs occupy a significant volume fraction and strongly influence TE properties. Initially used to reduce lattice thermal conductivity^20^, GB engineering has since expanded to enhance electrical conductivity^21^ and the Seebeck coefficient^22^ through control over boundary resistance^23^, energy filtering effects^24^, and complexion formation^12,25^. More recently, it has also been applied to improve the thermal stability^26^, and the broader role of GBs in governing both transport and stability of TE systems has been reviewed^27–30^. At the same time, GBs can act as fast diffusion pathways for reactive species, making them both functional interfaces and potential degradation pathways^31^. This duality of GBs makes GBs a compelling target for the rational design of multifunctional TE materials that combine high zT and long-term chemical stability. Despite this progress, the potential of GB engineering for mitigating chemical degradation has received less attention to date.

**Fig. 1:**
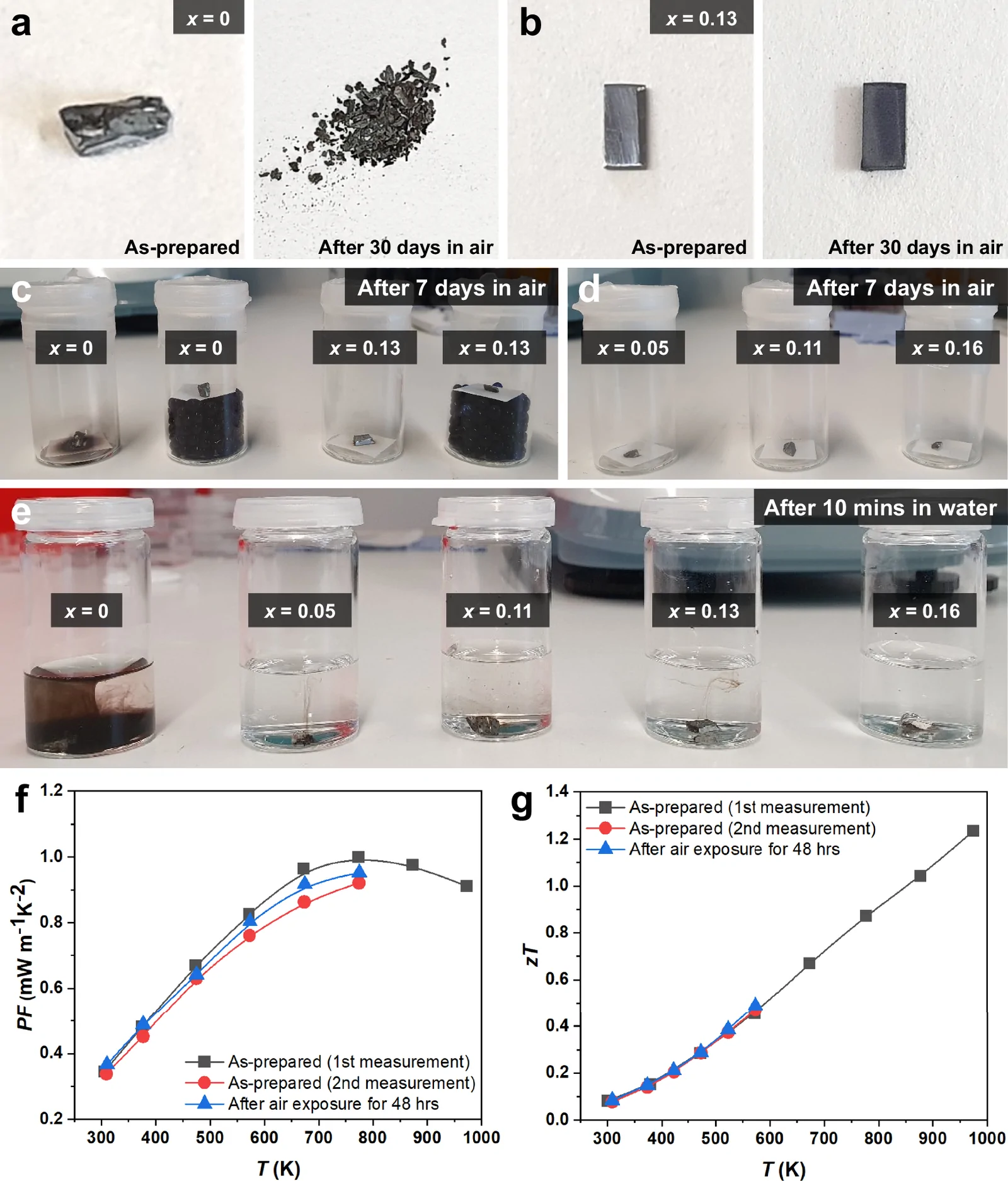
Enhanced chemical stability of Y_2.1_Te_3_Bi_*x*_ for air and water exposure by Bi incorporation. **a**, **b** Optical images comparing the air stability of (**a**) Y_2.1_Te_3_ and (**b**) Y_2.1_Te_3_Bi_0.13_ in the as-prepared state and after 30 days of exposure to open air. **c**, **d** Optical images of as-prepared Y_2.1_Te_3_Bi_*x*_ (*x* = 0, 0.05, 0.11, 0.13, and 0.16) samples after 7 days of air exposure. For *x* = 0 and 0.13, additional samples were stored in vials containing silica gel (dark material at the bottom of the right-hand-side vials) to distinguish the effects of dry and humid air. Samples were placed on filter paper to capture visible signs of degradation. **e** Optical images of the air-exposed samples after immersion in water for 10 min. **f**, **g** Power factor (*PF*) and thermoelectric figure-of-merit (*zT*) of Y_2.1_Te_3_Bi_0.13_ before and after air exposure for 48 h. Scale bars in (**c**–**e**) represent 2 cm. Source data are provided as a Source data file.

Motivated by this knowledge gap, we focus on the multifaceted role of Bi in Y_2_Te_3_, an emerging n-type TE material with intrinsically low lattice thermal conductivity, first identified in 2021^32^. Our previous work demonstrated that introducing excess Y and minor Bi incorporation modifies charge carrier scattering at GBs and increases the Seebeck coefficient, leading to a peak zT of 1.23 at 970 K for Y_2.1_Te_3_Bi_0.13_ with a two-fold increase in zT than Y_2.1_Te_3_^22^. However, the chemical stability and degradation behavior of these materials were not investigated in that study. In the present work, we identified a striking contrast in environmental durability. While pristine Y_2.1_Te_3_ rapidly disintegrates into powder within 30 days of air exposure, Y_2.1_Te_3_Bi_0.13_ preserves its structure over the same period (Fig. 1a, b). This contrast raises fundamental questions, such as: How does Y_2_Te_3_ degrade in reactive environments? What specific role does Bi play in impeding this process?

Here, we elucidate the moisture-induced degradation mechanism of Y_2_Te_3_ and the stabilizing role of Bi using a combination of scanning transmission electron microscopy (STEM), atom probe tomography (APT), and density functional theory (DFT). We show that H_2_O, rather than O_2_, drives degradation by infiltrating along GBs, where it initiates Y–O–H formation, Te redistribution, and chemo-mechanical breakdown. Bi incorporation drastically suppresses this process through segregation at GBs, thereby reducing H_2_O ingress and preserving both structural integrity and TE performance. DFT calculations confirm both the thermodynamic and kinetic tendency for Bi segregation at GBs, and Bi at GBs traps the H_2_O-related species. Our findings establish solute-driven GB engineering as a strategy for linking chemical durability with thermoelectric performance in air-sensitive TE materials.

## Results

### Bi-induced suppression of chemical degradation under humid conditions

The chemical stability of Y_2.1_Te_3_Bi_*x*_ was first evaluated under exposure to both ambient air and water. The as-prepared *x* = 0 and 0.13 samples were placed in sealed glass vials, with and without silica gel to control humidity (Figure S1). The two compositions exhibit distinct degradation behaviors after 7 days of air exposure (Fig. 1c). For *x* = 0, visible discoloration and decomposition were observed in the sample without silica gel, as evidenced by the stained filter paper. Notably, the *x* = 0 samples stored in dry air (with silica gel) also showed no signs of degradation. This result indicates that moisture (H_2_O), rather than oxygen, is the primary factor triggering the chemical breakdown of Y_2.1_Te_3_. This conclusion was further supported by thermogravimetric analysis (TGA) under controlled atmospheres: dry air (N_2_/O_2_) induced negligible mass gain (< 0.5% after 40 min) even upon heating to 420 °C to accelerate degradation, while humid N_2_ (N_2_/H_2_O) caused a rapid weight increase (> 10% after 40 min) with diffusion-limited kinetics (Figure S2)^33,34^.

In contrast, all Bi-containing samples remained intact both with and without silica gel after 7 days of air exposure, showing that even ~1 at.% Bi is sufficient to markedly improve chemical stability (Fig. 1d). The water immersion test showed the same trend. The Bi-free sample (*x* = 0) immediately decomposed upon immersion and fully disintegrated within an hour (Fig. 1e). Bubbling was observed during the decomposition due to gas evolution (Figure S3). In contrast, Bi-containing Y_2.1_Te_3_ samples degrade much more slowly (Fig. 1e). These results highlight that Bi also effectively retards the chemical breakdown by water. This stability difference also affected functional performance, as Bi-free Y_2.1_Te_3_ became electrically non-contactable just after 1 h of air exposure (Figure S4), while Y_2.1_Te_3_Bi_0.13_ retained its TE transport properties and *zT* after 48 h of air exposure (Fig. 1f, g and Figure S5). Based on these observations, both *x* = 0 and *x* = 0.13 samples (hereafter, denoted as Y_2.1_Te_3_ and Bi-Y_2.1_Te_3_) were further examined using advanced microstructural and spectroscopic analyses to elucidate their respective degradation pathways.

### Microstructural evolution and degradation pathway

**Fig. 2:**
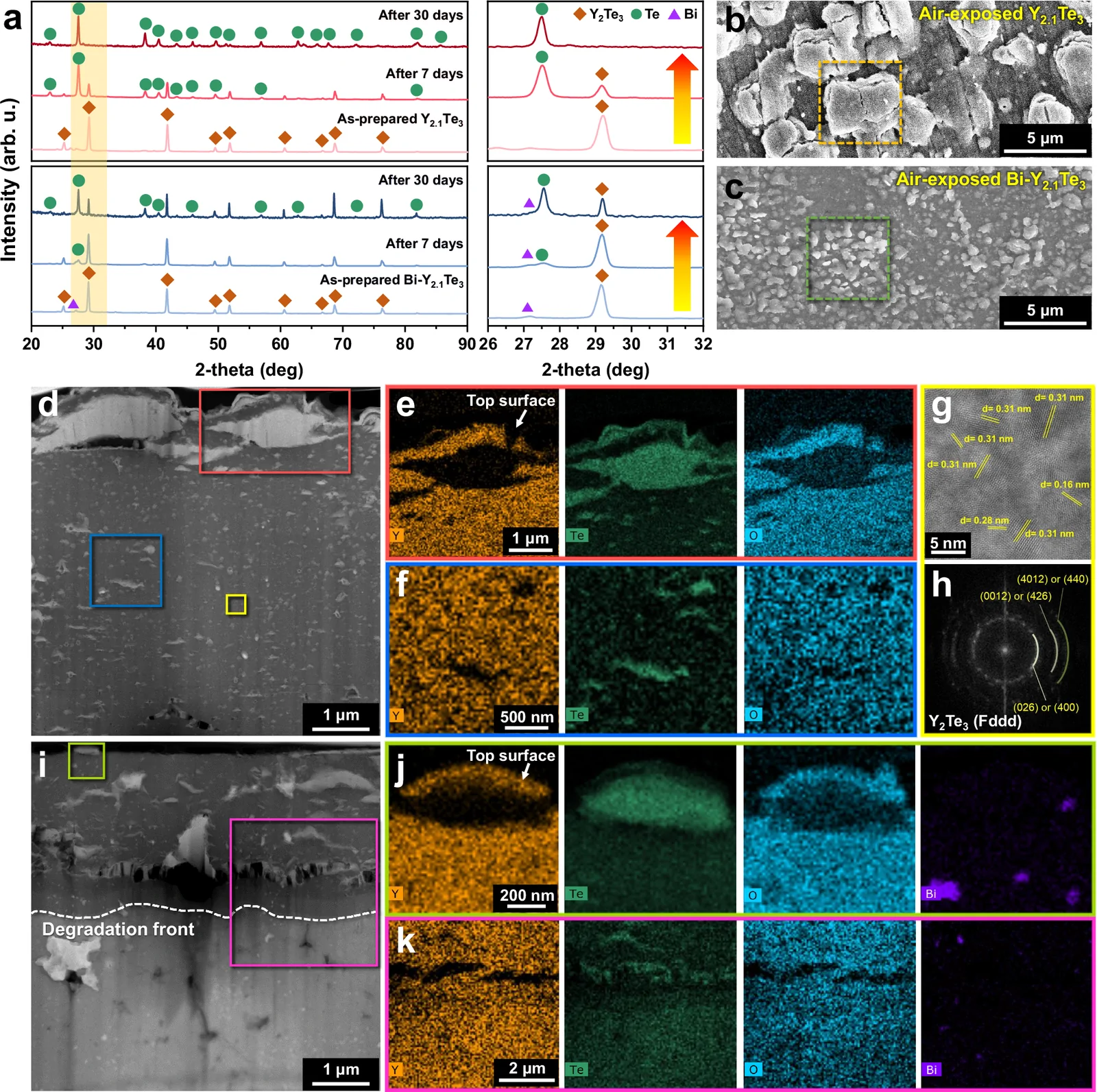
Structural characterization of Y_2.1_Te_3_ and Bi-Y_2.1_Te_3_ after air exposure. **a** X-ray diffraction patterns of the Y_2.1_Te_3_ and Bi-Y_2.1_Te_3_ samples after air exposure. The magnified patterns (right) are extracted from the shaded region in the left panel. **b**, **c** SEM surface images of Y_2.1_Te_3_ after 7 days and Bi-Y_2.1_Te_3_ after 30 days of air exposure, respectively. TEM lamellae were prepared from the dotted regions. **d**–**h** STEM analysis of the 7-day air-exposed Y_2.1_Te_3_: **d** cross-sectional HAADF-STEM image and **e**, **f** EDS elemental maps from the squared regions marked in (**d**); **g** High-resolution HAADF-STEM image of the degraded matrix (area marked in (**d**)), and (**h**) its fast Fourier-transform pattern. **i**–**k** STEM analysis of 30-day air-exposed Bi-Y_2.1_Te_3_: **i** cross-sectional HAADF-STEM image and **j**, **k** corresponding EDS elemental maps from the squared region marked in (**i**). The analysis is based on a single dataset without independent replicate measurements. Source data are provided as a Source data file.

X-ray diffraction (XRD) confirmed progressive phase evolution of Y_2.1_Te_3_ and Bi-Y_2.1_Te_3_ during air exposure (Fig. 2a). The diffraction peaks of the as-prepared samples matched the Y_2_Te_3_ phase (PDF#01-081-7493). The Bi-Y_2.1_Te_3_ additionally exhibited a metallic Bi peak (PDF#05-01-0225), consistent with discrete Bi particles as previously reported^22^. After 7 days of air exposure, the intensity of Y_2_Te_3_ peaks decreased for both samples, while peaks of elemental Te appeared (PDF#00-036-1452). These results indicate that moisture ingress induces decomposition of the Y_2.1_Te_3_ matrix into Y-oxide compounds and elemental Te. Notably, no crystalline Y-oxide phases were detected in the XRD patterns, likely due to their amorphous or nanocrystalline nature as further confirmed by Fourier-transform infrared (FT-IR) spectroscopy and high-resolution STEM discussed below.

The intensity of Te peaks continued to increase with prolonged exposure. After 30 days, the Y_2.1_Te_3_ completely disintegrated into fine powders and exhibited only Te peaks, whereas Bi-Y_2.1_Te_3_ retained its bulk integrity and preserved the Y_2_Te_3_ diffraction peaks (Fig. 2a). A pristine metallic surface could be readily recovered by polishing. Note that the calculated X-ray penetration depth is only ~2 μm at 2θ = 27.5°, so the degradation observed in Fig. 2a mainly reflects the near-surface region.

Scanning electron microscopy (SEM) and energy-dispersive X-ray spectroscopy (EDS) analyses of the disintegrated 7-day air-exposed Y_2.1_Te_3_ powder revealed a porous surface (Figure S6). This porous structure is associated with gas evolution similar to that observed during water exposure, along with chemical phase separation into Y,O-rich and Te-rich regions after degradation (Figure S7). FT-IR spectroscopy of the degraded powder confirmed the formation of Y–O–H compounds, with characteristic O–H bending (1632 cm^−1^) and stretching (3438 cm^−1^) peaks (Figure S8)^35^. According to previous reports, the degradation products can be YOH (oxyhydride), YO_2_H (oxyhydroxide), and Y(OH)_3_ (hydroxide), which are thermodynamically stable solid phases under ambient conditions^35–37^.

Phase separation became visually evident at the surface. The pristine metallic surfaces of as-prepared samples (Figure S9) transformed into surfaces covered with distinct Te nodules and an oxidized Y-rich surface after air exposure (Fig. 2b, c; Spot EDS in Figure S10). The Te nodules ranged from 2 to 5 μm in Y_2.1_Te_3_ after 7 days of exposure, while those in Bi-Y_2.1_Te_3_ were much smaller (0.5–1 μm) after 30 days.

STEM-EDS mapping of the as-prepared samples revealed homogeneous Y and Te distributions with discrete Bi particles in Bi-Y_2.1_Te_3_ (Figure S11). The average grain size was smaller for Bi-Y_2.1_Te_3_ (153 ± 49 nm) than for Y_2.1_Te_3_ (314 ± 115 nm) (Figure S12), indicating that Bi suppressed grain growth during sintering^22^. Cross-sectional STEM-EDS analyses after 7 and 30 days of air exposure identified Te nodules beneath the sample surface (Fig. 2d, i). After 7 days, numerous micron-sized Te nodules were distributed throughout the degraded Y_2.1_Te_3_ matrix (Fig. 2e, f), consisting of pure elemental Te as confirmed by XRD and fast Fourier-transform (FFT) analysis (Figure S13).

Given that intrinsic Y vacancies in Y_2_Te_3_, Y atoms preferentially diffuse and react with moisture to form Y–O–H phases by breaking Y–Te bonds. As local charge neutrality is disrupted, the remaining Te^2–^ species become unstable and are oxidized to elemental Te, accounting for the residual Te domains. The corresponding matrix exhibited strong Y and O signals and no discernible grain structure. High-resolution HAADF-STEM further revealed the transformation of Y_2.1_Te_3_ grains into nanocrystalline domains ( ~5 nm), as confirmed by FFT (Fig. 2g, h). Such nanocrystallization accounts for the absence of crystalline Y–O–H phases in the XRD results.

For the Bi-Y_2.1_Te_3_ sample exposed to air for 30 days, STEM-EDS analyses revealed a much thinner Te-depleted region (~6 µm) compared to the whole-lamella degradation observed in Y_2.1_Te_3_ after 7 days, along with smaller Te nodules on the surface (Figs. 2i, j). In the middle of the lamella, a ripped region surrounded by Te indicated chemo-mechanical failure caused by phase transformation into Y–O–H and Te. A clear degradation front separating the degraded regions from the underlying Y_2.1_Te_3_ was identified by image contrast (Fig. 2i). Across this front, EDS maps showed a Te depletion from the degraded surface (31.6 ± 6.7 at.%) than the Y_2.1_Te_3_ bulk below (49.2 ± 7.8 at.%) and O enrichment (40.0 ± 6.7 at.% (top); 25.7 ± 3.0 at.% (bottom)), whereas Y concentration remained nearly constant (28.4 ± 6.3 at.% (top); 25.2 ± 5.9 at.% (bottom)) (Fig. 2k). A complementary EDS line scan across the degradation front directly visualized these compositional changes (Figure S14). These results indicate that the overall Y content of the matrix is preserved during degradation; the degradation front is therefore governed by Y–O–H formation and associated Te redistribution.

Integrating these observations, the net chemical degradation reactions involve H_2_O ingress, Y–O–H formation, local Te segregation, and H_2_ gas evolution:

1 $${{{\rm{Y}}}}_{2}{{{\rm{Te}}}}_{3}+{2{{\rm{H}}}}_{2}{{\rm{O}}}\to 2{{\rm{YOH}}}+3{{\rm{Te}}}+{{{\rm{H}}}}_{2}{{\uparrow }}$$

2 $${{{\rm{Y}}}}_{2}{{{\rm{Te}}}}_{3}+{4{{\rm{H}}}}_{2}{{\rm{O}}}\to {2{{\rm{YO}}}}_{2}{{\rm{H}}}+3{{\rm{Te}}}+{3{{\rm{H}}}}_{2}{{\uparrow }}$$

3 $${{{\rm{Y}}}}_{2}{{{\rm{Te}}}}_{3}+{6{{\rm{H}}}}_{2}{{\rm{O}}}\to {2{{\rm{Y}}}({{\rm{OH}}})}_{3}+3{{\rm{Te}}}+{3{{\rm{H}}}}_{2}{{\uparrow }}$$

The corresponding molar volume expansions are 2.4, 10.8, and 32.1% (Table S1; calculation method in Supplementary text S2), generating substantial internal stress that drives chemo-mechanical breakdown from dense bulk to powder.

Having identified the microstructural evolution and chemical reactions driven by H_2_O ingress, we next addressed how H_2_O penetrates the Y_2.1_Te_3_ matrix and what role Bi plays. We hypothesized that GBs mediate this process, since they typically act as high-diffusivity pathways with room-temperature transport orders of magnitude faster than the bulk lattice^38^. Notably, despite a smaller average grain size and therefore higher GB density (Figure S12), Bi-Y_2.1_Te_3_ exhibited superior chemical stability than Y_2.1_Te_3_, implying that factors beyond GB density govern the enhanced stability.

One plausible explanation is that Bi modifies the GB structure^39^, thereby affecting diffusivity and chemical reactivity^38^. Transmission Kikuchi diffraction (TKD) revealed that both samples consist mainly of randomly oriented grains with a high fraction (~99%) of high-angle GBs (HAGBs; misorientation >15°), and the overall GB character (HAGB to low-angle GB ratio and average misorientation) was nearly identical between the two samples (Figure S15). High-resolution STEM further confirmed the HAGB character of representative as-prepared Bi-Y_2.1_Te_3_ GBs, with distinct atomic arrangements between adjoining grains and a clear <0$\bar{3}$1> (426)// <013> (400) orientation relationship (Figure S16). These results indicate that Bi incorporation does not alter the crystallographic character or geometry of the GB network.

### Retarded chemical degradation via grain boundary segregation of Bi

The compositional evolution within the bulk and along GBs during the degradation was further investigated using APT, which is an advanced analytical technique that combines field ion microscopy with time-of-flight mass spectrometry^40^. APT can reliably detect light elements such as O, H, B, and C^8,41–43^. Owing to these unique capabilities, APT has been widely applied to elucidate oxidation and corrosion mechanisms across diverse material systems, including engineering alloys^42,44,45^ and heterogeneous catalysts^46,47^.

To precisely track compositional changes from equivalent regions, we performed a controlled environmental exposure on APT specimens^48^. Multiple APT specimens were prepared from specific locations of the as-prepared Y_2.1_Te_3_ and Bi-Y_2.1_Te_3_ samples: a subset was analyzed immediately, while the remaining tips were exposed to ambient air for 7 days on the silicon posts (Figure S17). After exposure, the tips were sharpened using a focused ion beam and then analyzed. The air-exposed specimens showed noticeable surface transformations during exposure due to the large surface-to-volume ratios of APT specimens (Figure S17).

**Fig. 3:**
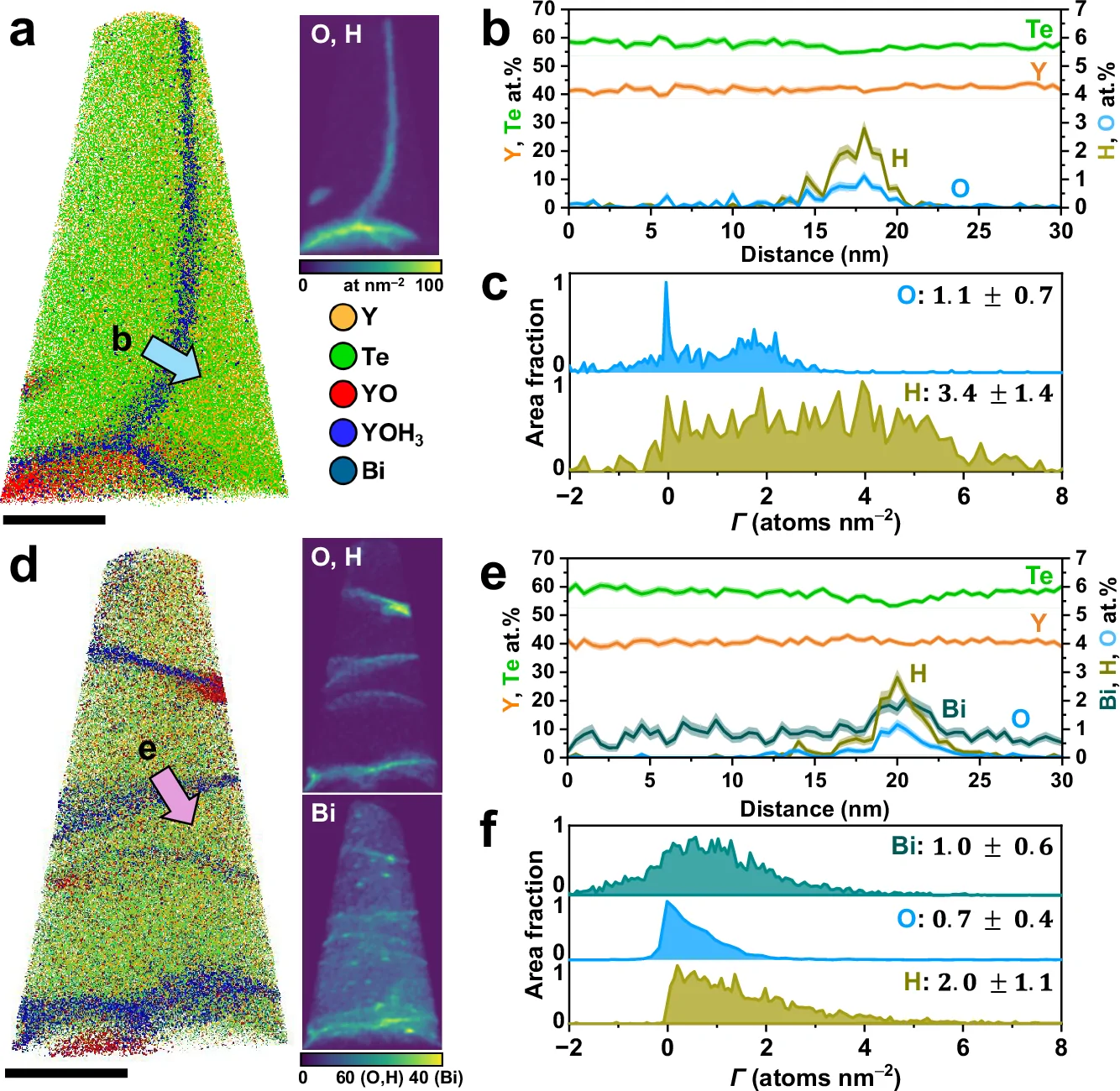
Atom probe tomography (APT) analysis of the as-prepared specimens. **a**, **d** APT reconstructions of (**a**) Y_2.1_Te_3_ and (**d**) Bi-Y_2.1_Te_3_. Insets display the 2D density plots of O + H and Bi atoms, highlighting their segregation at grain boundaries (GBs). **b**, **e** 1D concentration profiles across the GB marked by arrows in (**a**) and (**d**), respectively. Data in (**b**, **e**) are presented as bin-averaged concentration values, and the shaded regions indicate ±1 sigma statistical counting error. **c**, **f** The distribution of interfacial excess values (*Γ*) of O, H, and Bi atoms at GBs of (**c**) Y_2.1_Te_3_ and (**f**) Bi-Y_2.1_Te_3_ obtained by in-plane GB analyses, represented by area fraction. Scale bars are 50 nm for (**a**) and 100 nm for (**d**). Source data are provided as a Source data file.

The 3D atom map of the as-prepared Y_2.1_Te_3_ revealed a homogeneous Y and Te distribution close to the nominal stoichiometric composition (Y_2.2_Te_3_) (Fig. 3a). Distinct YO particles were observed within the matrix and adjacent to GBs. O and H segregation at GBs was resolved in the 2D density plot and 1D concentration profile, detected as YOH_3_^2+^ molecular ions (Fig. 3a, b). These molecular ions are likely to arise from the strong affinity of Y for O and H under the high electric field at GBs during APT analysis^36,49,50^. Accordingly, YOH_3_^2+^ ions were used as reliable GB indicators in all APT datasets.

The preferential enrichment of O and H at GBs, with negligible concentrations in grain interiors, is consistent with a moisture-driven degradation process and identifies GBs as primary diffusion pathways for H_2_O ingress. Potential sources of these light elements include residual impurities in raw materials, processing steps (e.g., ball milling and spark plasma sintering), or APT specimen preparation^51^. Similarly, the as-prepared Bi-Y_2.1_Te_3_ showed a uniform Y and Te distribution close to stoichiometric composition (Y_2.3_Te_3_) (Fig. 3d), with O, H, and additionally Bi segregated at GBs (Fig. 3e). The matrix Bi concentration of ~0.6 at.% (representing the bulk solubility) increased to ~2 at.% at GBs, and the smaller grain size of Bi-Y_2.1_Te_3_ allowed more GBs to be captured in the APT datasets.

To quantitatively compare O and H segregation at GBs between the two specimens, the in-plane Gibbsian interfacial excess (*Γ*) at GBs was determined from each 3D atom map using the convolutional neural network-based GB recognition technique (see Methods and Figure S18)^52^. The average *Γ* values of O and H decreased by ~40% after Bi incorporation, with high *Γ* area fractions (*Γ*_O_ > 2 and *Γ*_H_ > 4 atoms nm^–2^) significantly reduced in Bi-Y_2.1_Te_3_ (Fig. 3c, f).

**Fig. 4:**
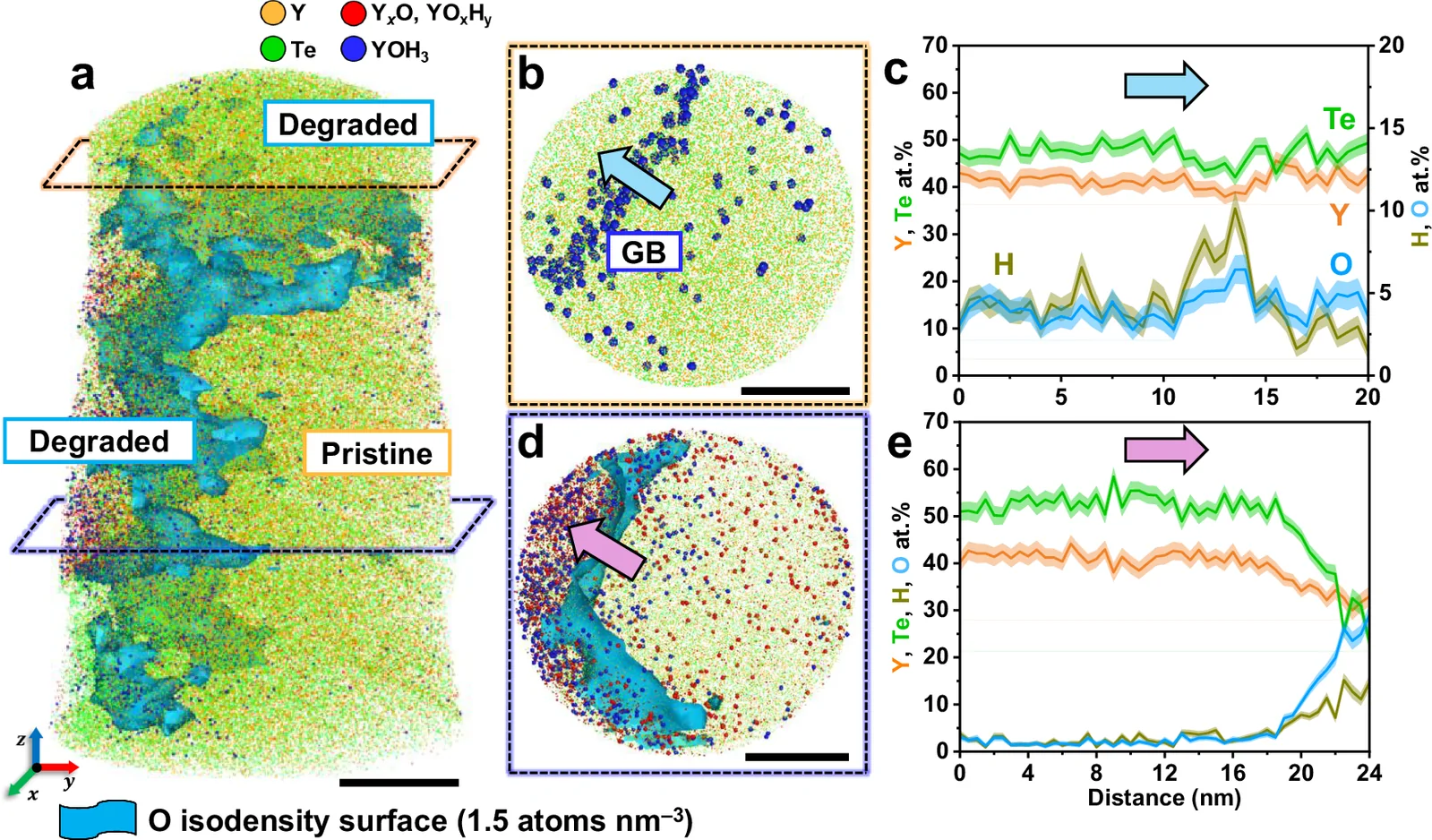
APT analysis of the air-exposed Y_2.1_Te_3_ for 7 days. **a** APT reconstruction of the air-exposed Y_2.1_Te_3_ for 7 days, showing degraded and pristine regions with the isodensity surface of O (1.5 atoms nm^–3^). **b** A thin slice of the dotted region in (**a**) and (**c**) 1D concentration profiles along the cyan arrow indicated in (**b**), representing the degraded GB. **d** A thin slice of the dotted region in (**a**) and (**e**) 1D concentration profile along the orange arrow indicated in (**d**), representing the interface between degraded and pristine regions. Data in (**c**, **e**) are presented as bin-averaged concentration values, and the shaded regions indicate ±1 sigma statistical counting error. Scale bars represent 10 nm. Source data are provided as a Source data file.

The APT results of air-exposed specimens provided further evidence for GB-mediated degradation. In the air-exposed Y_2.1_Te_3_, the O isodensity surface delineated the degraded region from the pristine region (Fig. 4a). A 5-nm-thick slice from the upper part revealed a GB (Fig. 4b), where O and H concentrations were nearly threefold higher than in the as-prepared state (Fig. 4c). The adjacent grain interior showed pronounced O and H enrichment (~5 at.%), in stark contrast to the negligible values in the as-prepared specimen (Fig. 4c). These results confirm that H_2_O is transported along GBs and subsequently diffuses into the grains, initiating Y oxidation accompanied by Te domain formation and yielding a Y-rich, Te-depleted matrix (Y:Te ≈ 1:1).

A second 5-nm slice from the lower region captured the interface between degraded and pristine regions (Fig. 4d). The pristine region retained a nominal stoichiometry with low O and H (1–2 at.%), but the degraded side showed marked O and H enrichment, severe Te depletion, and characteristic Y_*x*_O (*x* = 1, 2) and YO_*x*_H_*y*_ (*x, y* = 1, 2, and 3) molecular ions (Fig. 4e). This transitional region is consistent with the interreaction zone identified in the STEM-EDS maps, representing a pathway toward full phase separation (Fig. 2k).

A separate APT dataset of the air-exposed Y_2.1_Te_3_ captured residual Te segregated within the degraded Y_2.1_Te_3_ matrix, with a nearly pure Te region adjacent to O, H-enriched degraded zones (Figure S19). The spatial correlation between O, H enrichment and Te segregation indicates that Y–O–H phase growth along GBs drives local Te displacement, defining the reaction front that propagates into the grain interior.

**Fig. 5:**
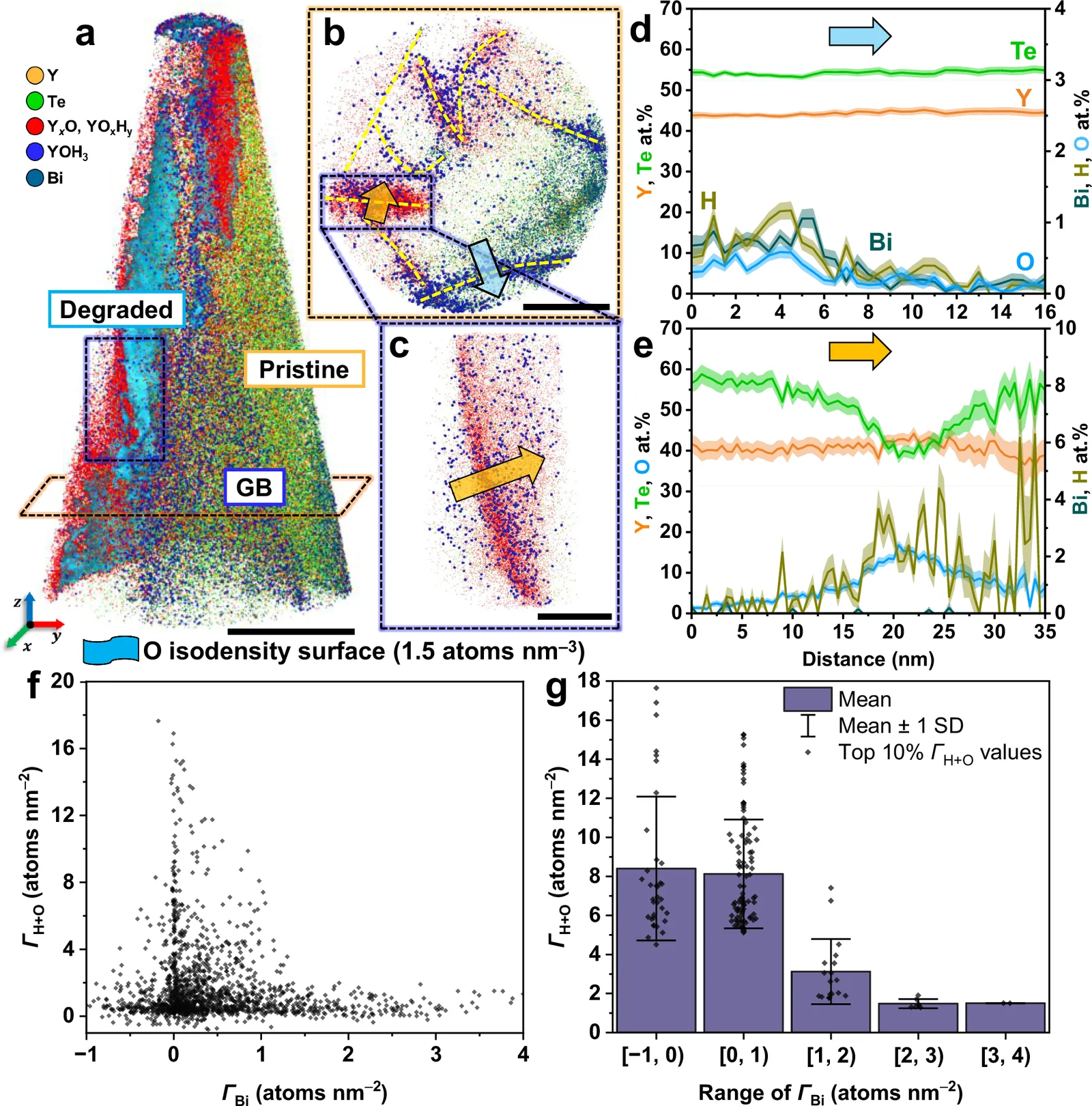
APT analysis of the air-exposed Bi-Y_2.1_Te_3_ for 7 days. **a** APT reconstruction of the air-exposed Bi-Y_2.1_Te_3_ for 7 days, showing degraded and pristine regions, and GBs with the isodensity surface of O (1.5 atoms nm^–3^). **b** A thin slice of the dotted region (orange) in (**a**), with GBs indicated by yellow dotted lines. **c** Side view of the dotted region (blue) in (**a**) and (**b**). **d** 1D concentration profile along the arrow indicated in (**b**), showing the pristine GB. **e** 1D concentration profile along the arrow indicated in (**b**) and (**c**), representing the degraded GB. Data in (**d**, **e**) are presented as bin-averaged concentration values, and the shaded regions indicate ±1 sigma statistical counting error. Scale bars are (**a**, **b**) 30 nm and (**c**) 20 nm. **f** Correlation between the Gibbsian interfacial excess of Bi (*Γ*_Bi_) and H + O (*Γ*_H+O_) at GBs from the APT dataset in (**a**). Each data point represents the average *Γ* values of the three vertices of an individual triangular GB mesh. **g** Mean *Γ*_H+O_ values of the top 10% highest *Γ*_H+O_ data points within each *Γ*_Bi_ interval from –1 to 4 atoms nm^–2^. Bars represent mean values, error bars indicate ±1 standard deviation (SD), and overlaid points show the individual top 10% *Γ*_H+O_ values used to calculate the mean and SD. The analysis was performed using *n* = 35, 101, 18, 7, and 2 data points for *Γ*_Bi_ intervals [–1, 0), [0, 1), [1, 2), [2, 3), and [3, 4) atoms nm^–2^, respectively. The analysis is based on a single dataset without independent replicate measurements. Source data are provided as a Source data file.

The 3D atom map of the air-exposed Bi-Y_2.1_Te_3_ specimen highlights the role of Bi segregation in chemical stability. Both degraded and pristine regions are distinguishable, separated by the spatial distribution of Y_*x*_O (*x* = 1, 2) and YO_*x*_H_*y*_ (*x, y* = 1, 2, and 3) species (Fig. 5a, b). Notably, Bi is preferentially located in pristine regions and segregated at GBs within the pristine region (‘pristine GB’), whereas only trace Bi (~0.1 at.%) was detected at GBs and adjacent grains in the degraded region (‘degraded GB’) (Fig. 5c). Concentration profiles across the pristine GB showed a Y:Te ratio close to the nominal stoichiometry with only minor O, H, and Bi, similar to GBs in as-prepared Bi-Y_2.1_Te_3_ (Fig. 5d). In contrast, the degraded GB exhibited markedly higher O (~15 at.%) and H (~4 at.%) with Te depletion, analogous to degraded GB in Y_2.1_Te_3_ (Fig. 5e). Possible effect of Ga implantation on such O and H enrichment was evaluated by analyzing the Ga distribution in the air-exposed APT specimens. Although residual Ga was detected in the datasets, Ga showed no spatial correlation with O-rich degraded regions, GBs, or degraded regions in both Y_2.1_Te_3_ and Bi-Y_2.1_Te_3_ (Figure S20), indicating that the observed O and H enrichment is not governed by Ga-induced damage.

The anticorrelation between Bi segregation and degradation extent is visualized by correlating *Γ*_Bi_ and *Γ*_H+O_ for each GB mesh (Fig. 5f): pronounced *Γ*_H+O_ increases were predominantly observed at low-*Γ*_Bi_ GBs, whereas high-*Γ*_Bi_ GBs absorbed little H or O. Quantitatively, the mean *Γ*_H+O_ of the top 10% highest data points decreased from ~8 atoms nm^–2^ for –1 ≤ *Γ*_Bi_ < 1, decreasing to ~3 and ~1.5 atoms nm^–2^ for 1 ≤ *Γ*_Bi_ < 2 and 2 ≤ *Γ*_Bi_ < 4, respectively (Fig. 5g). These results indicate that H_2_O preferentially attacks low-Bi GBs, whereas Bi-segregated GBs act as effective barriers against H_2_O ingress and the subsequent chemical degradation.

The retardation of H_2_O diffusion through GBs in Bi-Y_2.1_Te_3_ can be rationalized by a reduction in the GB interfacial free energy (${\gamma }_{{GB}}$), which increases the activation energy for H_2_O transport. Applying Cahn’s formalism and the Gibbs adsorption isotherm to our APT concentration profiles^53,54^, the change in ${\gamma }_{{GB}}$ induced by solute species *i* is given by:

4 $$\Delta {\gamma }_{{GB}}^{i}=-{k}_{B}T\cdot {\varGamma }_{i}\cdot {\mathrm{ln}}\left(\frac{{C}_{i}^{m}\left(x\right)}{{C}_{i}^{0}\left(x\right)}\right)$$

where ${\varGamma }_{i}$ is the interfacial excess of species *i* at position *x*, ${C}_{i}^{m}\left(x\right)$ is the measured local concentration of species *i*, and ${C}_{i}^{0}\left(x\right)$ is the reference concentration without segregation. Integration across the GB using the average *Γ*_Bi_ of 1.0 ± 0.6 atoms nm^–2^ measured by APT yields a ${\gamma }_{{GB}}$ reduction of 2.7 to 10.7 mJ m^–2^.

5 $${\Delta \gamma }_{{GB}}=\,{\gamma }_{{GB}}^{{Bi}}-{\gamma }_{{GB}}^{0}=\,\frac{{RT}}{2{a}^{2}{N}_{A}}{{\mathrm{ln}}}\left(\frac{{D}_{{GB}}^{{Bi}}}{{D}_{{GB}}^{0}}\right)$$

Combining this with the Borisov relationship (Eq. (5)) linking GB interfacial energy to GB diffusivity^55–57^ (see Supplementary text S2 for full derivation), and adopting an effective jump distance of *a* = 10 Å based on the Y_2.1_Te_3_ lattice parameters (a = 12.2 Å, b = 8.6 Å, c = 25.9 Å) determined by XRD, the corresponding increase in activation energy ${\Delta Q}_{{GB}}$ ranges from 3.25 kJ mol^–1^ to 12.9 kJ mol^–1^. The corresponding ratio ${D}_{{GB}}^{{Bi}}/{D}_{{GB}}^{0}$ from the ${\Delta Q}_{{GB}}$ is estimated to lie between 0.27 and 0.006. These results imply that Bi-induced reductions in ${\gamma }_{{GB}}$ can suppress GB diffusivity by nearly two orders of magnitude.

**Fig. 6:**
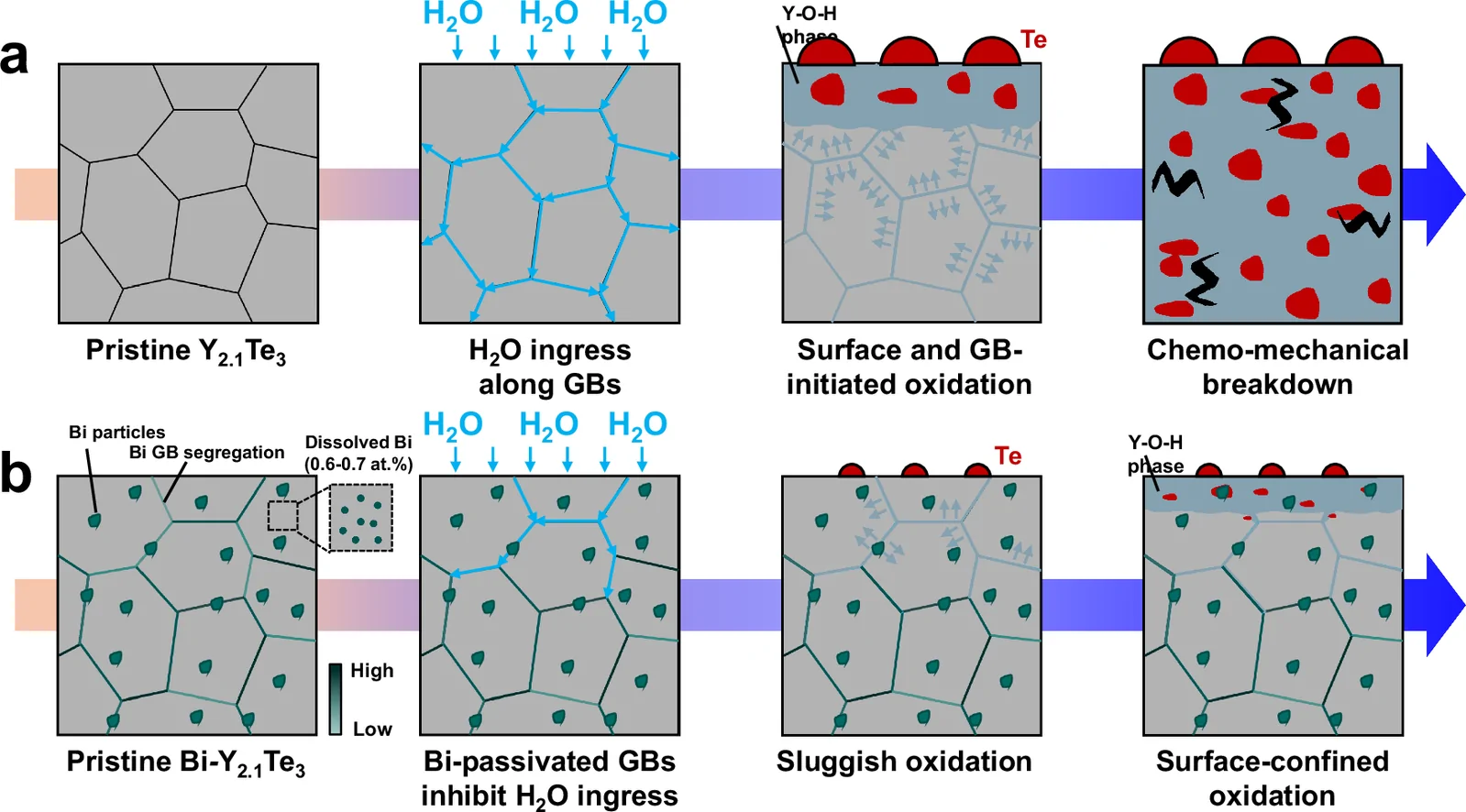
Schematic illustration of the degradation mechanism in Y_2.1_Te_3_ and its mitigation by Bi incorporation. **a** In Y_2.1_Te_3_, H_2_O rapidly diffuses along GBs, initiating GB oxidation that subsequently propagates into grain interiors. The associated phase transformation and volume expansion lead to chemo-mechanical failure of the matrix. **b** In Bi-Y_2.1_Te_3_, Bi segregation at GBs effectively suppresses H_2_O ingress and retards overall degradation. GBs with lower Bi content remain more vulnerable and are preferentially degraded.

By integrating all experimental results, the degradation mechanism is summarized in Fig. 6. In Y_2.1_Te_3_, degradation begins with the surface oxidation followed by rapid H_2_O diffusion along GBs, which accumulates and initiates local oxidation propagating into grain interiors (Fig. 6a). This oxidation breaks Y–Te bonding to form a nanocrystalline Y–O–H matrix with discrete Te nodules, and the accompanying volumetric expansion causes chemo-mechanical failure that disintegrates the bulk into powder. In Bi-Y_2.1_Te_3_, Bi segregation at GBs forms a chemically passivated interface that impedes H_2_O ingress, delaying GB oxidation and structural breakdown (Fig. 6b). Although localized degradation can still occur at GBs with lower Bi content, the overall suppression preserves the microstructural integrity of Bi-Y_2.1_Te_3_ during prolonged air exposure.

### Thermodynamic and kinetic origins of Bi segregation: Implications for multifunctional GB engineering

Experimental observations of Bi segregation at GBs raise another important question as to whether this segregation behavior represents a universal thermodynamic tendency of Bi in Y_2_Te_3_ or is merely a product of specific non-equilibrium synthesis methods such as ball milling and spark plasma sintering. To address this question, we performed first-principles DFT calculations to investigate the thermodynamic driving force, kinetic accessibility, and chemical environment modifications associated with Bi segregation.

As a preliminary step, we examined Bi defect energetics on the Y_2_Te_3_ (026) surface, the dominant facet observed in XRD (Figure S21). All Bi-related defects exhibited enhanced stability near the surface compared to bulk, with interstitial Bi (Bi_i_) and Bi adatoms showing strong surface stabilization (Figure S22a). A control calculation with interstitial Y (Y_i_) did not exhibit favorable surface segregation behavior, confirming that this surface affinity is specific to Bi (Figure S23). Gibbs free energies of binding (Δ*G*_bind_) of Bi defects as a function of Δ*μ*_Te_ further identified Bi_Te_ as the most stable defect under Te-poor (Y-rich) conditions, consistent with our synthesis environment and previous literature (Figure S22b; see Supplementary text S3)^58^.

**Fig. 7:**
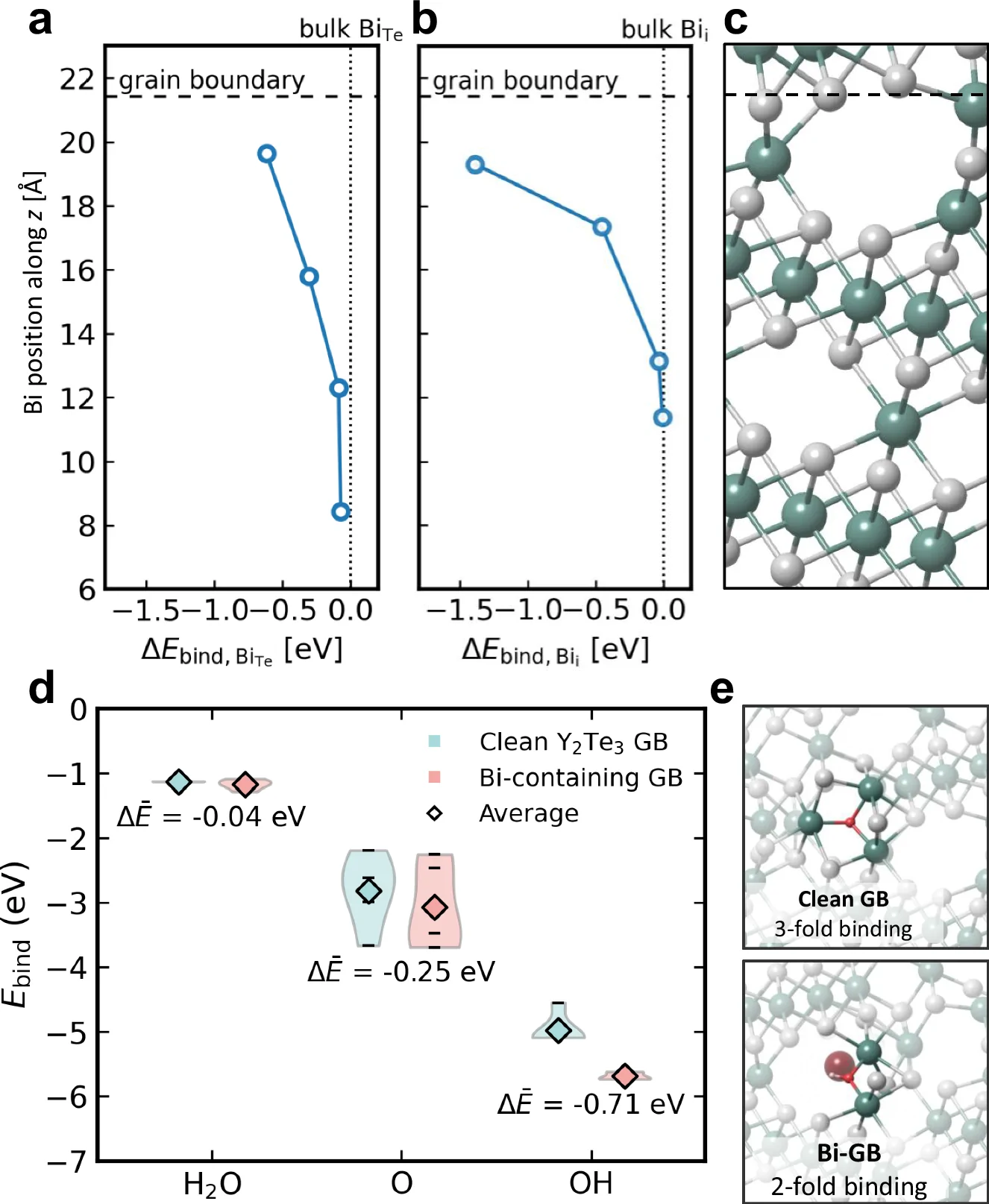
Binding energy behavior of Bi defects and H_2_O-related species at GBs. **a**–**c** Relative binding energy of Bi_Te_ (**a**) and Bi_i_ (**b**) as a function of distance from the GB plane with (**c**) the atomic structure of the Σ3 GB (teal: Y, white: Te). The zero reference corresponds to the bulk defect energy, and horizontal dashed lines indicate the GB core. **d** Binding energy distributions of H_2_O, O, and OH adsorbed at clean (blue) and Bi-containing (red) GBs. The shaded violin regions represent the distribution of calculated binding energies for different adsorption configurations, the short horizontal ticks indicate individual DFT-calculated structures, and diamond markers indicate average values. **e** Representative adsorption configurations of OH at clean and Bi-containing GBs (red: O, pink: H, dark red: Bi). Source data are provided as a Source data file.

Extending the analysis from surfaces to GBs, we modeled a symmetric Σ3 tilt GB as a computationally tractable representative of the experimentally observed HAGBs. Δ*G*_bind_ at the GB showed that Bi_Te_ was favored under Te-poor conditions, Bi_Y_ was favored under Te-rich, and Bi_i_ is metastable throughout the considered Δ*μ*_Te_ range (Figure S24). Despite its metastable character, Bi_i_ remains kinetically important because interstitial species generally exhibit substantially higher mobility than substitutional defects. Relative stabilization energies (Δ*E*_bind_) of Bi_Te_ and Bi_i_ as a function of distance from the GB plane (Fig. 7a–c) show that both defects become progressively more stabilized toward the GB, with Bi_i_ exhibiting the largest stabilization near the GB plane. This result indicates that kinetically accessible interstitial Bi species are efficiently trapped at GBs.

These thermodynamic findings are supported by climbing image-nudged elastic band (CI-NEB) calculations of Bi migration pathways in bulk and GB environments (Figure S25)^59^. APT measurements indicate that ~0.6 at.% of Bi resides within the bulk lattice (Fig. 3e). Given the ordered interstitial channels in bulk Y_2_Te_3_, we considered interstitial Bi diffusion as the primary transport mechanism.

In the bulk, Bi_i_ migrates with moderate intralayer (interlayer) barriers of 0.45 eV (0.61 eV) and readily transforms into thermodynamically stable substitutional configurations at neighboring vacancies (barrierless when a neighboring V_Te_ is present; 0.54 eV (0.43 eV) when a neighboring V_Y_ is present). At the GB, Bi_i_ migration is even more facile (0.46 eV) and conversion to Bi_Te_ upon encountering V_Te_ occurs with a low barrier of 0.25 eV (Figure S25b). In contrast, migration between neighboring Bi_Te_ sites exhibits a substantially higher barrier of 2.49 eV, implying that once substitutional Bi defects are formed at the GB, they become kinetically immobilized. Although diffusion prefactors were not explicitly evaluated, the migration barrier is expected to provide the dominant contribution when comparing relative diffusion kinetics for the same diffusing species within the same host lattice.

Combined with the thermodynamic segregation tendency (Fig. 7), these kinetic results support a mechanism in which metastable yet mobile Bi interstitials migrate through ordered-vacancy channels and become trapped at GBs as substitutional defects under realistic synthesis and annealing conditions. This trapping mechanism is further supported by our calculation of the work of GB separation (see Supplementary text S4), which shows that the presence of Bi does not compromise intrinsic GB cohesion, allowing Bi to remain stably incorporated without inducing mechanical instability.

In addition to the thermodynamic and kinetic aspects of Bi segregation, we investigated how Bi incorporation modifies the local electronic structure of Y_2_Te_3_. Projected density of states and Bader charge analyses revealed that Bi_Te_ behaves as a shallow acceptor-like defect in bulk and surface environments, but switches to a donor-like character at GBs (Figs. S26 and S27). In contrast, Bi_i_ is consistently donor-like regardless of its location, and Bi_Y_ remains electronically inert. This amphoteric character, governed by the local bonding geometry, modifies the GB chemical reactivity toward H_2_O-related species and helps explain the previously reported nonlinear carrier concentration trends in Y_2.1_Te_3_Bi_*x*_ (see Supplementary text S5). This result clarifies how Bi simultaneously enhances the chemical stability and electrical performance of Y_2_Te_3_.

To elucidate the role of Bi in moisture-induced degradation, we investigated H_2_O adsorption and its dissociated derivatives (O and OH) at clean and Bi-containing GBs (Fig. 7d; see Supplementary text S6). While molecular H_2_O shows comparable binding at both GBs (Δ*E*_*bind*_ ≈ 0.06 eV), dissociated species (particularly OH) are significantly stabilized by Bi segregation (Δ*E*_*bind*_ ≈ 0.7 eV), indicating that Bi preferentially stabilizes dissociated O- and H-containing species at the GB rather than intact molecular H_2_O. Interestingly, this stabilization is not due to direct Bi-OH bonding (nearest distance 4.21 Å); OH instead remains coordinated to Y atoms with the preferred motif shifting from threefold to a stronger two-fold coordination at Bi-rich GBs (Fig. 7e). This would be because the donor-like character of Bi defects at GBs enhances electron density and thus likely stabilizes OH adsorption even with reduced coordination. These findings agree well with our experimental APT observations, which reveal clear segregation of O and H at GBs together with the formation of Y–O–H phases.

Overall, our DFT results show that Bi segregation to surfaces and GBs of Y_2_Te_3_ is energetically favorable and kinetically accessible. Once segregated, Bi modifies the local electronic and chemical environment of GBs, thereby preferentially stabilizing dissociated H_2_O-derived species. Together with the experimental APT results, these findings explain the reduced susceptibility of Bi-rich GBs to moisture-induced chemical degradation.

## Discussion

In this study, we elucidated the fundamental degradation mechanism of Y_2.1_Te_3_ under ambient conditions and clarified the stabilizing effect of Bi incorporation. We showed that the chemical instability of Y_2.1_Te_3_ originates from GB-mediated moisture transport, which initiates oxidation and ultimately leads to chemo-mechanical failure. Remarkably, incorporation of only ~1 at.% of Bi was sufficient to substantially retard degradation through preferential GB segregation. Bi enrichment at surfaces may also contribute to the improved resistance against the initial surface reaction, as suggested by the smaller Te nodules formed on Bi-Y_2.1_Te_3_ after air exposure. However, the presence of a degraded near-surface region after prolonged exposure shows that surface enrichment alone does not fully block moisture ingress. Instead, APT reveals a direct anticorrelation between Bi segregation and H plus O accumulation at individual GBs, indicating that Bi-segregated GBs control the subsequent propagation of degradation into the bulk. We therefore consider Bi surface segregation as a secondary contribution that delays the onset of degradation, while GB segregation provides the dominant mechanism for suppressing moisture transport into the bulk and resulting chemical degradation.

By combining TKD, STEM, and in-depth APT analyses, we confirmed that this Bi-induced GB stabilization arises from a chemical effect. DFT calculations further revealed that Bi segregation to GBs is both energetically and kinetically favorable, facilitated by kinetically accessible interstitial diffusion through ordered-vacancy channels and subsequent stabilization at GB regions. The calculations further showed that Bi incorporation electronically modifies GB environments and preferentially stabilizes reactive O- and H-containing species at GBs. These results provide a mechanistic explanation for how Bi incorporation simultaneously enhances both chemical stability and thermoelectric performance in Y_2.1_Te_3_.

Beyond Y_2.1_Te_3_, this strategy is readily extendable to other emerging thermoelectric systems, such as Mg_3_(Sb,Bi)_2_ and CoSb_3_-based skutterudites, where GB engineering has already been shown to improve *zT* ^60,61^, but chemical instability under air and humid environments remains a major challenge^6,13,62^. Overall, our work highlights the great potential of GB segregation as a unified solution for simultaneously achieving high performance and long-term durability in thermoelectric and other air-sensitive energy materials.

## Methods

### Sample preparation, stability test, and thermoelectric property measurements

Y_2.1_Te_3_Bi_*x*_ (*x* = 0, 0.05, 0.11, 0.13, 0.16) samples were synthesized by mechanical alloying of stoichiometric amounts of Y pieces (99.9%, Alfa Aesar), Te granules (99.99%, Alfa Aesar), and Bi pieces (99.999%, MaTeck) in a glove box using a SPEX 8000D ball mill for 13 h, followed by spark plasma sintering at 1123 K and 50 MPa for 3 min (FCT System GmbH) in a 12.7 mm diameter graphite die, as described in our earlier work^22^.

For long-term air stability tests, Y_2.1_Te_3_ and Y_2.1_Te_3_Bi_0.13_ were exposed to open laboratory air for 30 days (relative humidity: 60–80%). For the controlled stability test, Y_2.1_Te_3_Bi_*x*_ samples were stored in parafilm-sealed glass vials for 7 days (relative humidity: 64%). For *x* = 0 and 0.13 (denoted Y_2.1_Te_3_ and Bi-Y_2.1_Te_3_, respectively), an additional vial containing silica gel was used to assess the effect of moisture. Air-exposed samples were also immersed in DI water to evaluate aqueous chemical stability.

The electrical conductivity and Seebeck coefficient were measured using a commercial LSR-3 system (Linseis) with a four-probe method under a He atmosphere. The thermal conductivity was determined according to *κ* = *DρC*_*p*_, where *D* is the thermal diffusivity, *ρ* is the density, and *C*_*p*_ is the heat capacity. The *D* was measured using laser-flash analysis (LFA, Linseis). The *C*_*p*_ was approximated using the Dulong-Petit limit, while the *ρ* was calculated from the measured dimensions and mass of each specimen.

### Thermogravimetric analysis

All TGA measurements were conducted on ~50 mg of Y_2.1_Te_3_. Dry-air TGA was performed on a TG209 F1 Libra (NETZSCH) under a mixed N_2_ (80%)/O_2_ (20%) flow at 50 ml/min with an additional N_2_ purge at 30 ml/min, ramping from 50 to 420 °C at 10 °C/min. Humidity-controlled TGA was performed on a STA8122 (Rigaku) coupled with a HUM-1 humidity generator, which provides a humidified N_2_ carrier gas with controlled relative humidity (RH) up to ~90% RH. Mass change was recorded at room temperature and under two modes: (i) constant RH at 75%, and (ii) gradually increasing RH from 75% to 90% (+ 2.5% every 10 min). Both experiments ran for 85–90 min.

### Scanning electron microscopy and X-ray diffraction

Sample surfaces were observed by SEM (Zeiss Sigma 360 VP) at a working distance of 10 mm and an acceleration voltage of 10 kV (imaging) and 15 kV (EDS mapping). XRD analyses were performed on a Rigaku Smartlab (micro-area setup, Cu Kα radiation), with peak assignment based on the PDF-2 2024 database. The X-ray penetration depth was estimated using the online calculator provided by TU Wien (gixa.ati.tuwien.ac.at/tools/penetrationdepth.xhtml) for Y_2.1_Te_3_ with a density of 5.43 g/cm^3^ and Cu Kα energy of 8047.8 eV.

### Scanning transmission electron microscopy

Electron-transparent TEM lamellae were prepared using a Scios 2 dual-beam focused ion beam (FIB, Thermo Fisher) with a ~3 μm-thick protective Pt cap over a 2 × 10 μm^2^ region of interest. The extracted lamellae were initially thinned to ~200 nm using 30 kV Ga^+^ ion milling, and were finally thinned to ~100 nm with surface cleaning at 5 kV and 2 kV. STEM imaging and EDS were conducted on a Titan Themis 60-300 probe-corrected microscope (Thermo Fisher) at 300 kV with a Super-X EDS detector. HAADF imaging used a 78–200 mrad collection angle, 68 pA probe current, and 23.6 mrad semi-convergence angle.

### Fourier transform infrared spectroscopy

FT-IR was performed using a Thermo Scientific Nicolet iS50 spectrometer (400 to 3600 cm^–1^), 1.928 cm^–1^ resolution. The decomposed Y_2.1_Te_3_ powder was prepared as a KBr pellet, and data were analyzed using OMNIC software (Version 9.2.106).

### Transmission Kikuchi diffraction

The TKD lamellae were prepared on Cu half-grids using a Helios 5 CX DualBeam FIB (Thermo Fisher). The lamellae were initially thinned to around 400 nm using a 30 kV Ga^+^ ion beam and further reduced to ~300 nm at 5 kV, 68 pA, sufficient to obtain TKD patterns from both Y_2.1_Te_3_ and Bi-Y_2.1_Te_3_. TKD patterns were acquired using a direct electron detector (Clarity Plus) integrated into the Helios 5 CX system, operated at 30 kV, 2.8 nA, and 4 mm working distance. Patterns were analyzed by spherical indexing in EDAX OIM Analysis 9.1 (bandwidth 127), which enhances pattern recognition for low-symmetry structures.

### Atom probe tomography

APT specimens were prepared by FIB (Scios 2 DualBeam, Thermo Fisher) following the standard lift-out protocol^63^. Final low-kV (5 kV) milling was applied to reduce the Ga^+^ implantation. APT analyses were performed using a CAMECA LEAP 5000X HR system in pulsed UV laser mode. The analyses were conducted at a pulse frequency of 100 kHz, a detection rate of 0.5–1%, the base temperature of 50 K, and a laser pulse energy of 30–60 pJ. Data reconstruction and analysis were carried out using the AP Suite 6.3.2 software provided by CAMECA Instruments. For the air-exposed APT specimens, FIB-cut lamellae mounted on Si posts were exposed to ambient air for 7 days while still in their lift-out form, and the FIB sharpening into APT needles was performed only after this exposure (Figure S17). Although several of these tips fractured during APT analysis, representative datasets could be obtained because the analyzed volumes, exposed by the post-exposure FIB sharpening, corresponded to partially degraded reaction-front regions rather than fully disintegrated outer regions. These transitional regions retained sufficient mechanical integrity for field evaporation under the APT measurement conditions described above. The APT mass spectra (Figs. S28 and S29), ionic compositions (Table S2), and atomic compositions (Table S3) of each specimen are provided in Supplementary Information.

In-plane interfacial excess at GBs was determined using APT_GB software^52^. The software identified GB planes using a pre-trained convolutional neural network and discretized them into triangular mesh elements with a unit area of ~8 nm^2^ ^41^. For each mesh, ladder diagrams were generated at its three vertices to compute local interfacial excess values. The correlation of interfacial excess values between Bi and H + O was assessed by averaging the *Γ* values at the vertices of triangular meshes and plotting them. Meshes located near YO particles were excluded from correlation analysis due to artificially elevated *Γ* values caused by the presence of particles.

### Density functional theory

Density functional theory (DFT) calculations were carried out using the projector augmented wave (PAW) method as implemented in the Vienna Ab initio Simulation Package (VASP)^64^. The valence electron configurations explicitly included the 5d, 6s, and 6p orbitals for Bi, the 4s, 4p, 4d, and 5s orbitals for Y, and the 5s and 5p orbitals for Te. Exchange-correlation effects were treated using the generalized gradient approximation (GGA) within the Perdew-Burke-Ernzerhof (PBE) formulation^59^. To better capture the electronic properties of Y_2_Te_3_, an on-site Coulomb interaction was applied to the Y 4 d states via the DFT + *U* approach following the Dudarev formalism^65^, with an effective Hubbard U parameter (*U*_eff_) of 3.0 eV^66^.

A plane-wave energy cutoff of 450 eV was used throughout. The Brillouin zone was sampled using a k-point mesh corresponding to a reciprocal space resolution of 0.04 Å^–1^, resulting in a (2 × 3 × 1) grid and four irreducible k-points for bulk Y_2_Te_3_. Geometry optimizations of the bulk structure were performed until the residual stress dropped below 0.5 kbar and all atomic forces were less than 0.01 eV/Å. Electronic self-consistency was achieved with a convergence threshold of 10^–5^ eV, while ionic relaxations proceeded until the total energy change between steps was smaller than 10^–4^ eV. The fully relaxed lattice parameters of orthorhombic (*Fddd*) Y_2_Te_3_ were found to be a =  12.55 Å, b =  8.91 Å, and c =  26.61 Å. These optimized parameters were used for constructing supercells and surface/grain boundary models. Comprehensive computational setups for the surface and GB models, along with the specific details used for diffusion behavior in bulk and GB via the climbing-image nudged-elastic band (CI-NEB) method, are provided in Supplementary text S7.

## Supplementary information

Supplementary Information

Description of Additional Supplementary Files

Supplementary Data 1

Transparent Peer Review file

## Source data

Source Data

## Data Availability

The relevant data generated in this study are provided in the Supplementary Information. Source data are provided with this paper. All the raw data relevant to the study are available from the corresponding author upon request. Source data are provided with this paper.
