# Supplementary material for: Retarding moisture-induced chemical degradation of Yttrium Tellurides by tailoring grain boundary chemistry
Source: Nat Commun. 2026 Jul 25;17:9069. doi: 10.1038/s41467-026-76056-8 (PMC13519032; doi:10.1038/s41467-026-76056-8)
Supplement: Supplementary file 1 [file 41467_2026_76056_MOESM1_ESM.pdf]

Supplementary Information for

Retarding moisture-induced chemical degradation of Yttrium Tellurides by tailoring grain boundary chemistry

Kyuseon Jang[1,2]†✉, Jamil Ur Rahman[3]†, Su-Hyun Yoo[4]†, Chanwon Jung[5], Eric Woods[2], Ruben Bueno Villoro[2], Kornelius Nielsch[3], Christina Scheu[2], Yonghyuk Lee[6]✉, Pyuck-Pa Choi[1]✉, Ran He[3]✉, Siyuan Zhang[2]✉

[1]Department of Materials Science and Engineering, Korea Advanced Institute of Science and Technology (KAIST), Daejeon 34141, Republic of Korea

[2]Max Planck Institute for Sustainable Materials, Max-Planck-Straße 1, Düsseldorf 40237, Germany

[3]Thermoelectric Materials and Devices, IFW Dresden, Helmholtzstraße 20, Dresden 01069, Germany

[4]Digital Chemical Research Center, Korea Research Institute of Chemical Technology, Gajeong-ro 141, Daejeon, 34114, Republic of Korea

[5]Department of Materials Science and Engineering, Pukyong National University, Busan 48513, Republic of Korea

[6]Department of Chemical and Biochemical Engineering, Dongguk University, Seoul, 04620, Republic of Korea

†K. Jang, J.U. Rahman, S. Yoo contribute equally to this work.

✉Corresponding author Email: k.jang@mpi-susmat.de, yhyuk@dongguk.edu, p.choi@kaist.ac.kr, r.he@ifw-dresden.de, siyuan.zhang@mpi-susmat.de

Supplementary text

S1. Estimation of molar volume changes during moisture-induced degradation

To assess the chemo-mechanical impact of moisture-induced degradation of Y_2Te_3, the molar volume change associated with the proposed reaction pathways (Reaction (1) to (3) in the manuscript) was estimated. For each reaction, the total molar volume of solid products was calculated and compared with that of the pristine Y_2Te_3. Gaseous species (H_2) and liquid reactants (H_2O) were excluded from the volume calculation, as they do not contribute to the solid-state volume change relevant to mechanical stress development.

The molar volume (V) of each solid phase was calculated using

$$V = \frac{M}{\rho}$$

where M is the molar mass and ρ is the density of the phase. The densities and molar masses used for Y_2Te_3, YOH, YO_2H, $Y(OH)_3$, and Te are summarized in Table S1. Based on these values, the molar volume of pristine Y_2Te_3 was determined to be 103.24 cm^3/mol, while the molar volumes of YOH, YO_2H, $Y(OH)_3$, and Te were 22.15, 26.50, 37.51, and 20.46 cm^3/mol, respectively.

For each degradation reaction, the total molar volume of the solid products was calculated by summing the molar volumes of the corresponding phases according to their stoichiometric coefficients. The relative volume change ΔV was then evaluated as

$$\Delta V = \frac{V_{products} - V_{Y2Te3}}{V_{Y2Te3}} \times 100\%$$

Using this approach, the reaction

$$Y_2Te_3 + 2H_2O \rightarrow 2YOH + 3Te + H_2\uparrow$$

was found to result in a modest molar volume expansion of around 2.4%. In contrast, the reactions form YO_2H and $Y(OH)_3$,

$$Y_2Te_3 + 4H_2O \rightarrow 2YO_2H + 3Te + 3H_2\uparrow$$

$$Y_2Te_3 + 6H_2O \rightarrow 2Y(OH)_3 + 3Te + 3H_2\uparrow$$

lead to larger molar volume expansions of approximately 10.8% and 32.1%, respectively.

These results indicate that progressive hydration and oxidation of Y_2Te_3 are accompanied by substantial solid-state volume expansion, particularly when Y-rich oxyhydroxide and hydroxide phases form.

S2. Linking GB interfacial energy to GB diffusivity by the Borisov relationship

The temperature dependence of GB diffusivity can be described by an Arrhenius-type equation[1]:

$$D_{GB} = D_0 \exp\left(-\frac{Q_{GB}}{RT}\right)$$

where D_{GB} is the GB diffusivity, D_0 is a pre-exponential factor related to the molecular jump frequency, and Q_{GB} is the activation energy for GB diffusion.

To conceptually connect changes in γ_{GB} to D_{GB}, we employed the Borisov relationship, which was originally proposed to link GB interfacial energy to GB self-diffusion in pure metals and has been used as a phenomenological framework for H and impurity diffusion in alloys[2–4]:

$$\gamma_{GB} = \frac{RT}{2a^2 N_A} \ln\left(\frac{D_{GB}}{D_L}\right)$$

where a is an effective atomic jump distance associated with GB diffusion, N_A is Avogadro's number, and D_L is the lattice diffusivity[4,5]. Taking the difference between Bi-$Y_{2.1}Te_3$ (γ_{GB}^{Bi}) and $Y_{2.1}Te_3$ (γ_{GB}^{0}), and assuming that D_L is comparable between the two specimens, $\Delta\gamma_{GB}$ is:

$$\Delta\gamma_{GB} = \gamma_{GB}^{Bi} - \gamma_{GB}^{0} = \frac{RT}{2a^2 N_A} \ln\left(\frac{D_{GB}^{Bi}}{D_{GB}^{0}}\right)$$

Combining this equation with the above equation, ΔQ_{GB} can be related to $\Delta\gamma_{GB}$ by

$$\Delta Q_{GB} = Q_{GB}^{Bi} - Q_{GB}^{0} = -2a^2 N_A \Delta\gamma_{GB}$$

These relations indicate that even modest reductions in γ_{GB} can exponentially

suppress D_{GB} by increasing Q_{GB}. Based on an effective jump distance of a =10 Å as a representative atomic length scale for GB diffusion and the given range of γ_{GB} from 2.7 to 10.7 mJ m^{-2}, the above equation yields ΔQ_{GB} values between 3.25 kJ mol^{-1} to 12.9kJ mol^{-1}. The corresponding ratio D_{GB}^{Bi}/D_{GB}^{0} is estimated to lie between 0.27 and 0.006.

S3. Surface segregation tendency and defect thermodynamics of Bi in Y_2Te_3

To further investigate the thermodynamic origin of Bi segregation behavior in Y_2Te_3, we examined Bi defect energetics on the Y_2Te_3(026) surface, which corresponds to the dominant crystallographic facet observed experimentally (Fig. S21). Three representative Bi defects were considered: Bi substituting for Y (Bi_Y), Bi substituting for Te (Bi_{Te}), and interstitial Bi (Bi_i). Their relative binding energies (ΔE_{bind}) were evaluated as a function of depth from the surface (Fig. S22a).

All three Bi defects exhibited enhanced stability near the surface compared to bulk-like regions, indicating a general thermodynamic preference for interfacial segregation. Among them, Bi_i showed the strongest surface affinity. Interestingly, although surface-site Bi_i was already strongly stabilized, the first subsurface configuration exhibited slightly lower energy. Structural relaxation revealed that the interstitial Bi partially displaced a neighboring surface Te atom and relaxed toward a geometry intermediate between subsurface and surface configurations, contributing to its enhanced stability.

We additionally considered Bi adatoms placed above the pristine surface without lattice substitution. Among the configurations examined, the most stable adatom geometry exhibited even stronger binding than all other Bi_i configurations, including surface and subsurface sites. This result further supports the strong interfacial affinity of Bi and is consistent with the experimentally observed Bi enrichment at surfaces and GBs.

To verify whether this behavior is specific to Bi, we performed a control calculation using interstitial Y (Y_i). In contrast to Bi_i, Yi exhibited strongly unfavorable binding near the surface and adatom regions (Fig. S23), indicating that the observed segregation tendency is not a generic feature of interstitial species in Y_2Te_3.

We further evaluated the Gibbs free energy of binding (ΔG_{bind}) for each Bi defect as a function of Te chemical potential ($\Delta\mu_{Te}$) (Fig. S24). Bi_{Te} was found to be most stable under Te-poor (Y-rich) conditions, consistent with the synthesis conditions and previous reports[6]. Under intermediate $\Delta\mu_{Te}$ conditions, multiple Bi defect configurations exhibited comparable stability, suggesting the possible coexistence of Bi_Y, Bi_{Te}, and Bi_i under realistic processing environments.

S4. Effect of Bi segregation on GB cohesion

To investigate whether Bi segregation induces mechanical embrittlement, we evaluated the work of separation (W_{sep}) for both clean and Bi-containing GBs. We considered multiple cleavage planes parallel to the GB interface to identify the minimum energy.

The calculated minimum W_{sep} values are 0.043 eV/Å^2 for the clean GB and 0.042 eV/Å^2 for the Bi-containing GB. This negligible difference indicates that Bi incorporation does not significantly alter the intrinsic decohesion resistance of the GB within DFT accuracy. These values suggests that the commonly reported embrittling effect of Bi is not strongly manifested in the clean GB structure considered in this work.

S5. Effect of Bi defects on the electronic structure of Y_2Te_3 at the surface, in the bulk, and at grain boundaries

Although Bi is isovalent with Y, it exhibits unexpected doping behavior in Y_2Te_3[6,7]. In our previous study, we also observed a nonlinear evolution of carrier concentration in $Y_{2.1}Te_3Bi_x$: an initial sharp drop to ~20% of the undoped level at low Bi content (x = 0.05), followed by a gradual increase with further doping (x = 0.11–0.16)[8]. This counterintuitive behavior suggests that the impact of Bi on electronic properties is not solely determined by its nominal valence but is strongly influenced by its specific defect configuration and local atomic environment.

To investigate the origin of this behavior, we conducted DFT calculations of the projected density of states (PDOS) and Bader charges for three representative Bi defects:

interstitial Bi (Bi_i), Bi substituting Y (Bi_Y), and Bi substituting Te (Bi_{Te}). For each defect type, we considered two representative positions within the (026) surface slab model: a surface-near site and the deepest subsurface site. Fig. S26 displays the PDOS profiles focusing on Bi orbital contributions near the band edges, alongside their corresponding Bader charges.

Among the configurations, Bi_i introduces shallow Bi p-states near the conduction band minimum (CBM) and exhibits a nearly neutral Bader charge, implying a weak donor-like nature. Bi_Y exhibits minimal effect of the host electronic structure and a moderately positive Bader charge, indicating that it is electrically inactive. In contrast, Bi_{Te} introduces pronounced Bi p-states near the valence band maximum (VBM) and shows a strongly negative Bader charge (≈ -1.2 e), indicating shallow acceptor-like behavior and potential p-type doping under surface and bulk conditions. These results suggest that both Bi_i and Bi_{Te} could contribute to carrier modulation in Y_2Te_3, but with different magnitudes and effects. Bi_{Te} is thermodynamically stable over a wide range of Te chemical potentials, making it a plausible dominant defect at low doping levels. Therefore, Bi_{Te} is potentially responsible for the experimentally observed reduction in carrier concentration.

To further examine how Bi behaves at grain boundaries (GBs), we extended our analysis to a Σ3 GB. At the GB, Bi_Y remains electrically inert, but both Bi_i and Bi_{Te} exhibit donor-like n-type characteristics, introducing additional states near the CBM and shifting the Fermi level into the conduction band (Fig. S27). Notably, Bi_{Te}, which acts as a p-type defect at the surface and bulk, shows n-type behavior at the GB. This contrast underscores its amphoteric nature, where the doping character is highly sensitive to the local bonding geometry and coordination[6].

This electronic duality offers a mechanistic explanation for the nonlinear carrier concentration trend in our previous experiments. At low Bi content, Bi_{Te} defects may dominate and reduce carrier concentration. As Bi concentration increases, segregation at GBs becomes more significant due to thermodynamically and kinetically driven processes (Fig. S25). Consequently, donor-like Bi_i or Bi_{Te} at GBs would highly emerge. This contributes to n-type doping and partial recovery of carrier concentration. These findings highlight the importance of considering defect site specificity when interpreting defect-induced doping effects in complex thermoelectric materials.

S6. Binding behavior of H_2O-related species at GBs

To further understand the role of Bi-segregated GBs in moisture-induced degradation, we evaluated the binding energies of representative H_2O-related species, including H_2O, O, and OH, at both clean and Bi-containing GBs (Fig. 7 in the main text). Unique adsorption sites were first identified using graph-based adsorption-site analysis[9], followed by pre-relaxation using the universal machine-learning potential MACE-MH-1[10]. The five most stable candidate structures for each species were subsequently refined using DFT relaxations.

Molecular H_2O exhibits relatively moderate binding energies ranging from –1.02 to –1.13 eV (average –1.11 eV) at the clean GB and from –1.09 to –1.29 eV (average –1.17 eV) at the Bi-containing GB. In contrast, dissociated species bind substantially more strongly. O exhibits binding energies ranging from –2.19 to –3.67 eV (average –2.82 eV) at the clean GB and from –2.26 to –3.70 eV (average –3.07 eV) at the Bi-containing GB. An even more pronounced effect is observed for OH, whose binding energies increase from –4.55 to –5.09 eV (average –4.98 eV) at the clean GB to –5.62 to –5.74 eV (average –5.69 eV) at the Bi-containing GB.

S7. Computational details

(Surface models) Y_2Te_3 was treated as a vacancy-ordered compound with intrinsic cation vacancies explicitly included in the crystallographic model. Surface calculations were conducted using slab models constructed within periodic boundary conditions, incorporating a vacuum region of at least 20 Å to eliminate spurious interactions between periodic images. The (026) surface was selected for detailed investigation, as it corresponds to the most prominent facet identified in X-ray diffraction measurements. Asymmetric slab geometries were generated based on the (1×1) surface unit cell, consisting of six atomic layers, with the bottom two layers fixed at their optimized bulk positions to simulate bulk-like constraints. Structural relaxation of the surface atoms was performed using the conjugate gradient algorithm. To minimize artificial dipole-dipole interactions arising from the slab asymmetry, a dipole correction was applied along the direction normal to the surface[11].

(Grain boundary models) Grain boundary (GB) structures were constructed by using Aimsgb,[12] based on a bulk Y_2Te_3 structure containing 16 formula units. Considering the presence of high-angle grain boundaries in experimental observations and the computational feasibility of DFT calculations, a $\Sigma3[0\bar{1}1]/(1\bar{1}\bar{1})$ symmetric tilt boundary was constructed. Various convergence tests were performed to ensure the reliability of the low-energy structure, given the limited number of prior GB studies on Y_2Te_3. Based on convergence tests on the interfacial gap and terminations between GB planes, $Y_{48}Te_{72}$ and $Y_{34}Te_{56}$ structures were selected as host configurations for Bi-incorporated GB. Additional convergence tests on supercell size indicated that 2×1 supercell is sufficient, as the energy difference between 2×1 and 2×2 supercells was within 50 meV/Bi atom. To construct Bi defects at the grain boundary, we considered both interstitial and substitutional configurations (i.e., Bi_i, Bi_{Te}, and Bi_Y). Bi-related defects were modeled using charge-neutral supercells, which allows us to study general trends of defect-related energies, local charge redistribution and donor-/acceptor-like electronic behavior at GB. Interstitial defect structures were constructed by employing a method based on the charge-density-based general cation insertion algorithm implemented in pymatgen[13,14]. For substitutional defects, Bi atoms were introduced by systematically replacing either Y or Te atoms in the pristine GB structure.

(CI-NEB calculations) Diffusion barriers of a Bi atom in bulk and GB of Y_2Te_3 were computed using the machine-learning-accelerated climbing-image nudged-elastic band (CI-NEB) method, as implemented in the AIDNEB framework[15–17]. For each diffusion pathway, a total of 9 images (including initial and final states) were used. The initial path was generated using the improved dimer projection (IDPP) interpolation scheme[18]. All NEB images were optimized until the maximum force perpendicular to the reaction coordinate was less than 0.02 eV/Å. The spring constant between images was set to 0.1 eV/Å, and the climbing-image scheme was employed to accurately locate the saddle point. To determine the diffusion pathway, a nearest-neighbor hopping mechanism was constructed based on the local atomic geometry. In the Y_2Te_3 structure, the diffusion channels are geometrically constrained and form a narrow and well-defined low-energy diffusion network due to the connectivity of available vacancy

sites. Alternative migration pathways are either symmetry-equivalent or involve significantly longer hops through sterically hindered regions, and are therefore not expected to contribute substantially to diffusion.

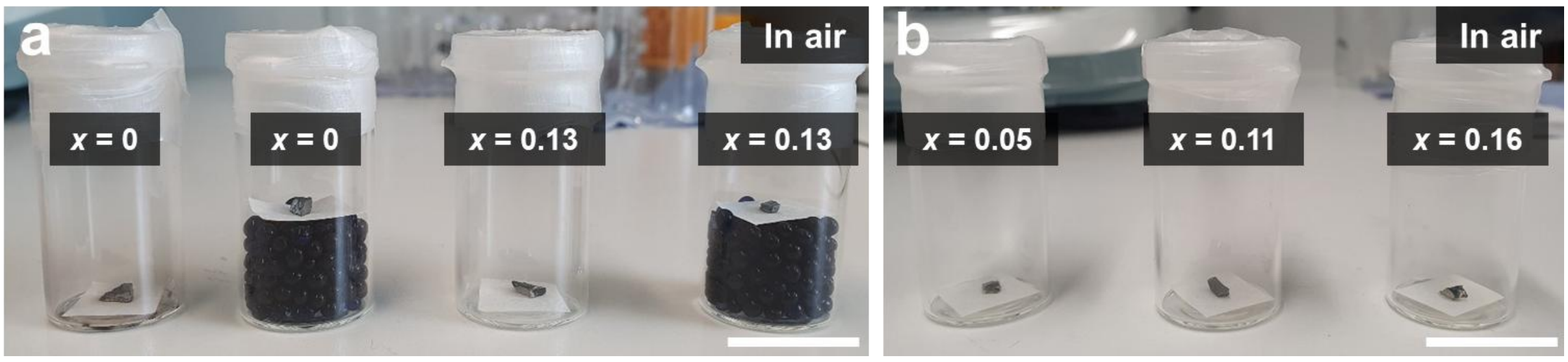

Figure S1. The images of as-prepared $Y_{2.1}Te_3Bi_x$ (x = 0, 0.05, 0.11, 0.13, 0.16) in vials before exposure to air for 7 days. (a) Samples of x = 0 and x = 0.13 in empty vials and one samples in vials filled with silica gel. (b) Samples of x = 0.05, 0.11, and 0.16 in empty vials. Scale bars represent 2 cm.

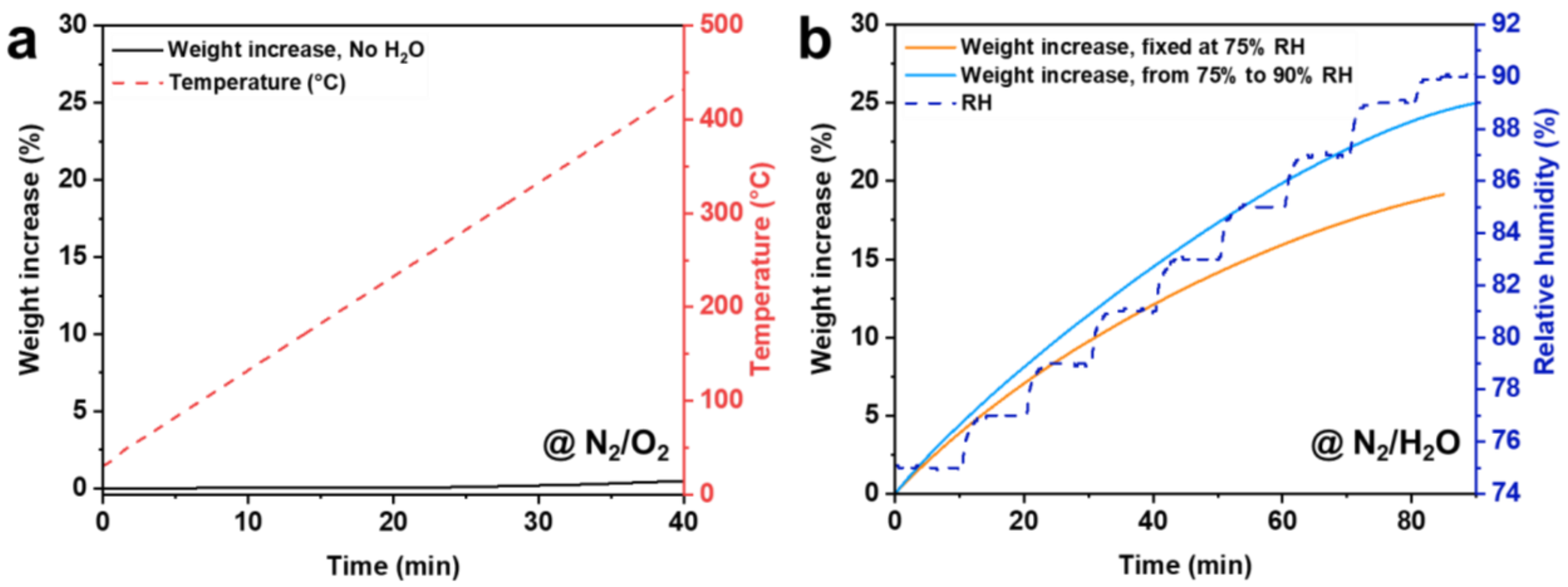

Figure S2. Thermogravimetric analysis (TGA) measurements. Time-dependent weight gain of $Y_{2.1}Te_3$ measured under (a) dry O_2 with increasing temperature and (b) humid air (N_2/H_2O, without O_2), where the relative humidity (RH) was constant at 75% and increased from 75% to 90% at room temperature. Overall weight gain rate increased further when the relative humidity was gradually raised from 75% to 90% compared with a fixed relative

humidity of 75%, showing that moisture governs the degradation.

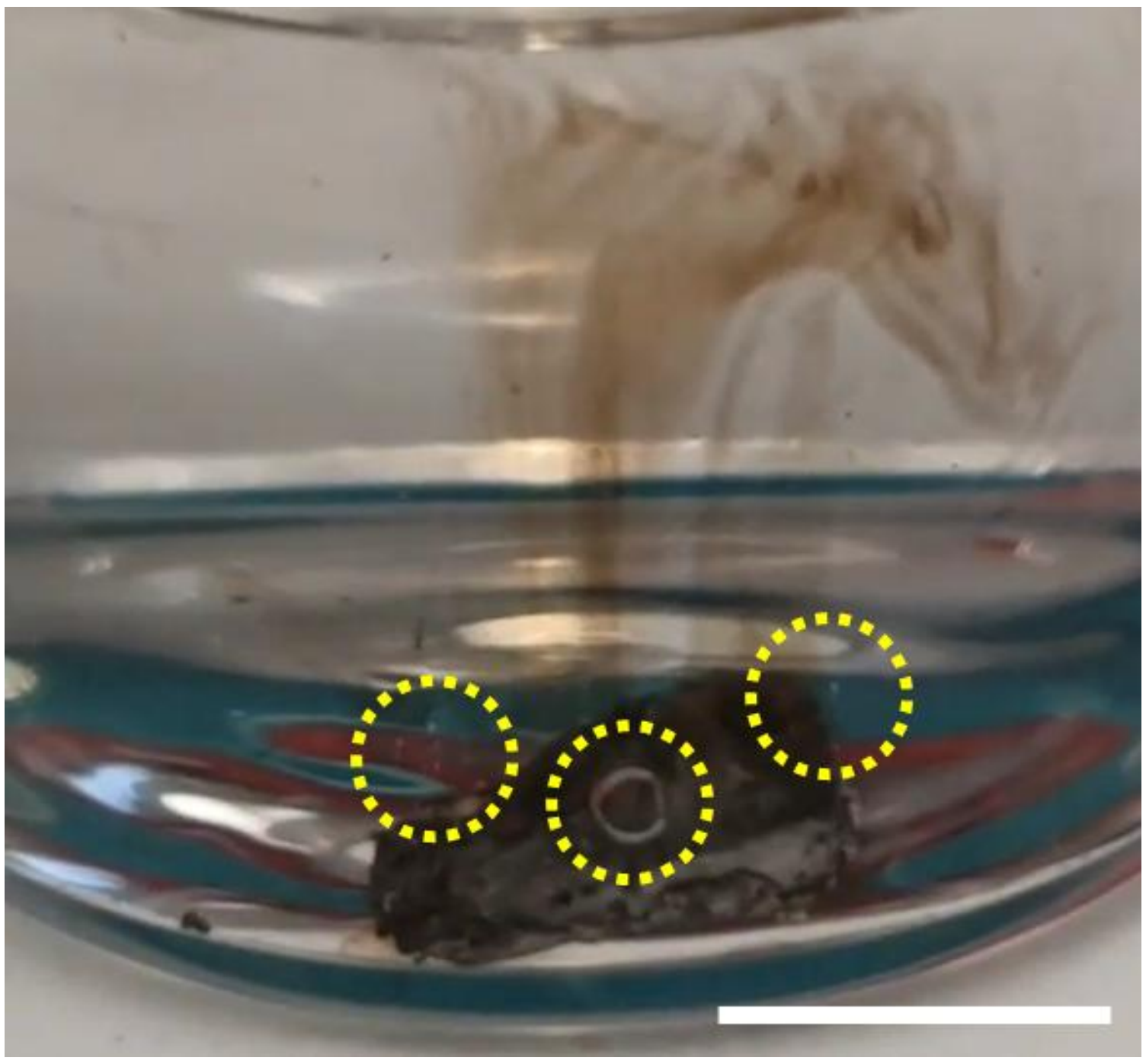

Figure S3. Optical image of bubble generation during the degradation of $Y_{2.1}Te_3$ in water. Yellow dotted circles indicate bubbles. Scale bar represents 5 mm.

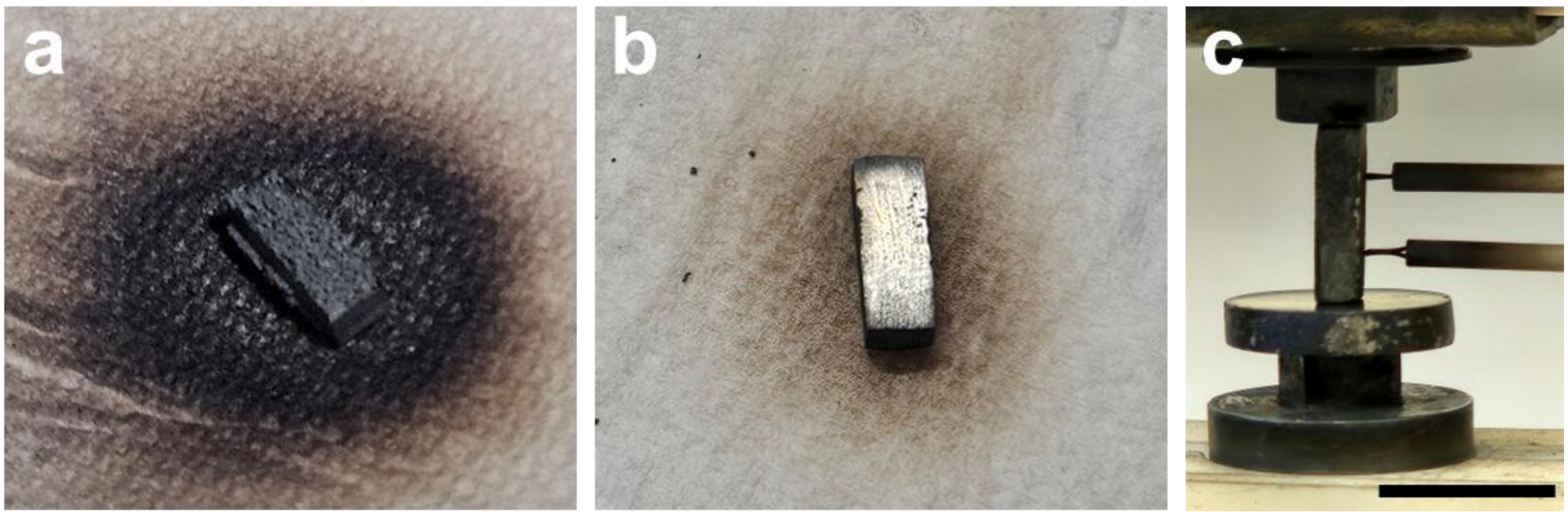

Figure S4. Electrical transport measurement of $Y_{2.1}Te_3$ after 1h of air exposure. (a) Optical image of the sample surface after the first electrical measurement and 1 hour of air exposure. (b) Metallic surface recovered after polishing. (c) Attempts to measure electrical transport properties after polishing; however, no reliable electrical contact could be established

since severe subsurface degradation makes the surface highly resistive. Scale bar in (c) represents 1 cm.

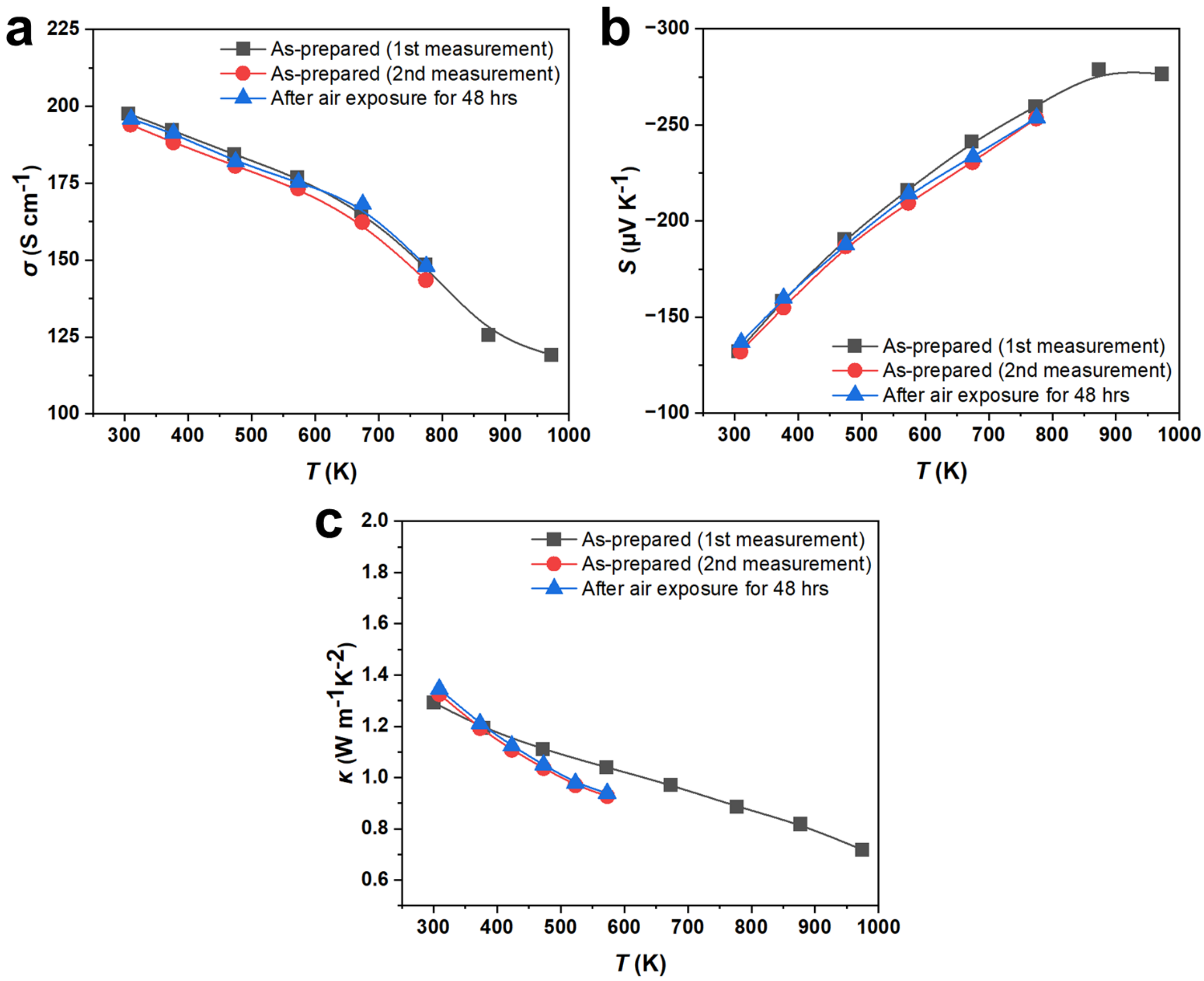

Figure S5. Thermoelectric transport properties of $Y_{2.1}Te_3Bi_{0.13}$ before and after 48 hours of air exposure. (a) Electrical conductivity, (b) Seebeck coefficient, and (c) thermal conductivity. The properties of the air-exposed sample remain unchanged within the measurement uncertainty.

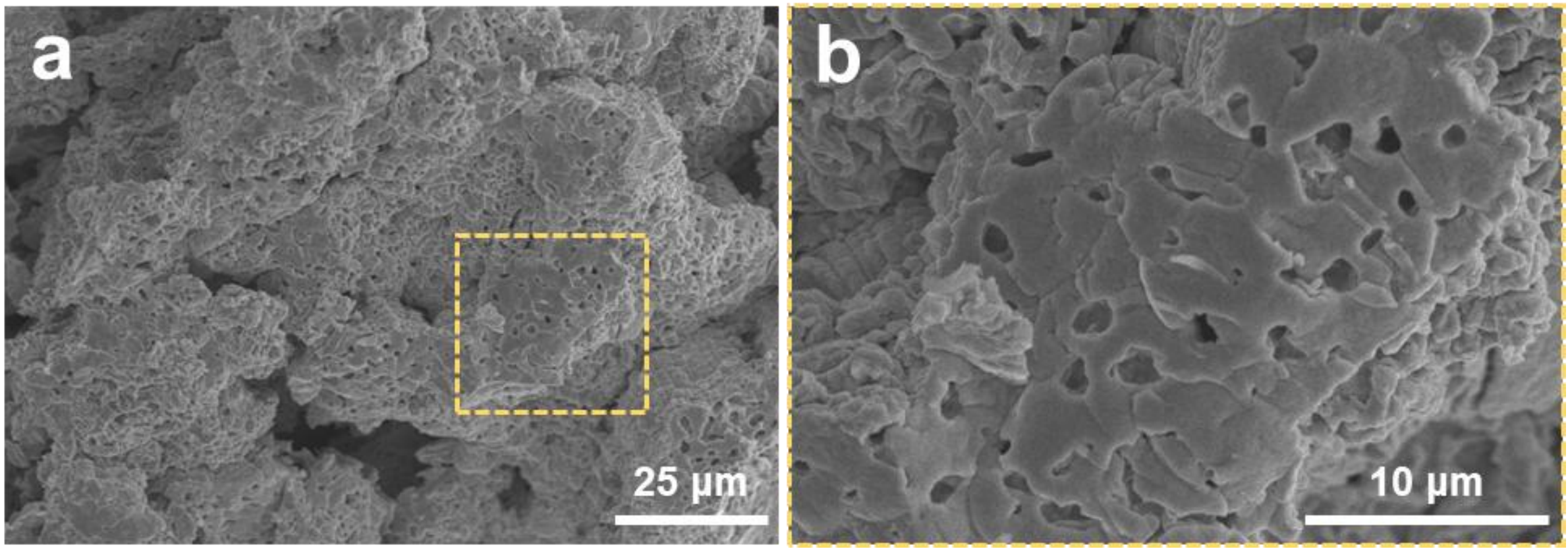

Figure S6. SEM surface images of degraded powder of the $Y_{2.1}Te_3$ sample after air exposure for 30 days. Images are taken in (a) low magnification and (b) high magnification as represented by a dotted square in (a).

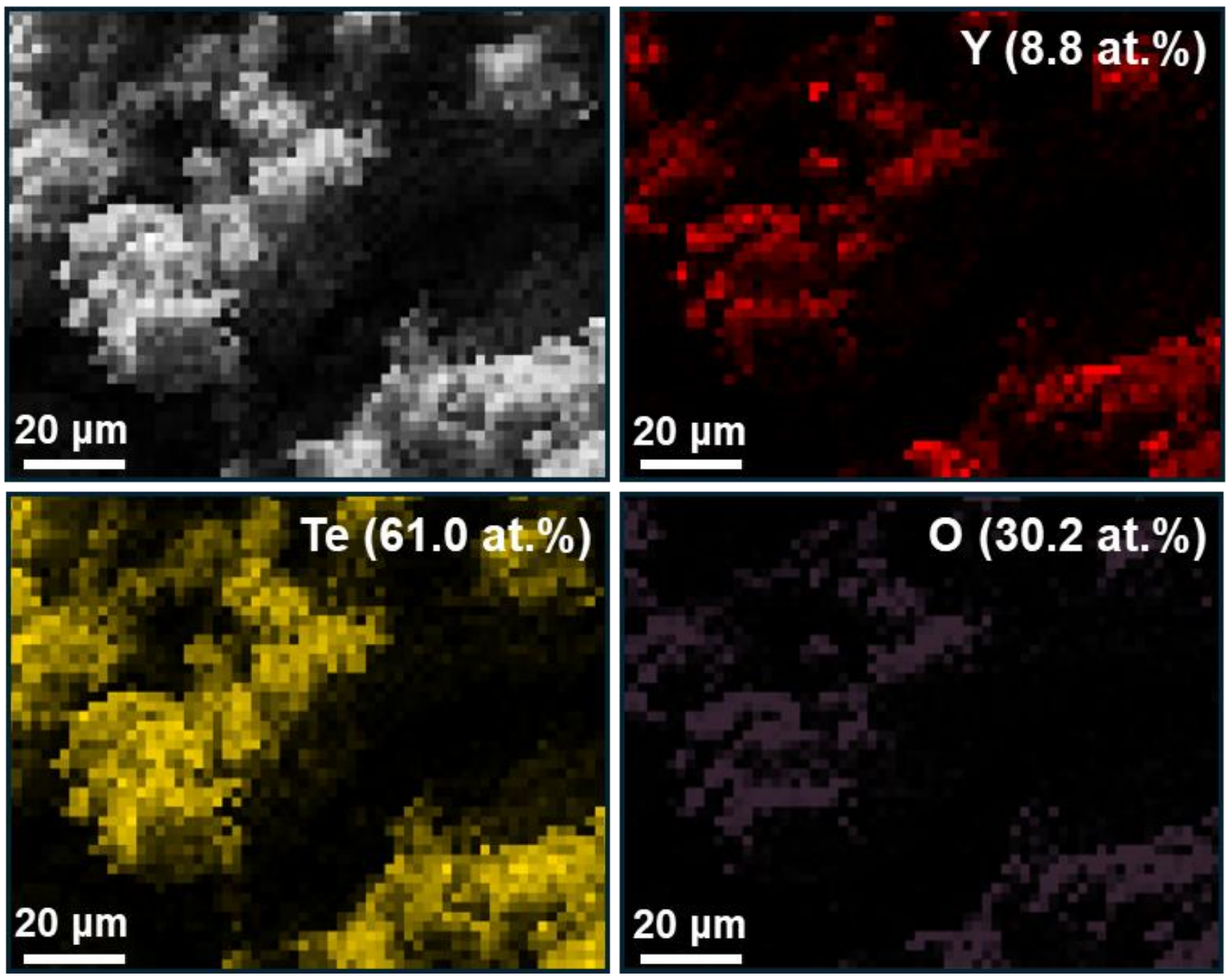

Figure S7. SEM-EDS composition mapping for degraded powder of the $Y_{2.1}Te_3$ after air exposure for 30 days. EDS maps and composition (unit: atomic percent) of Y, Te, and O show the heterogeneous elemental distribution and deviation from nominal stoichiometry after degradation.

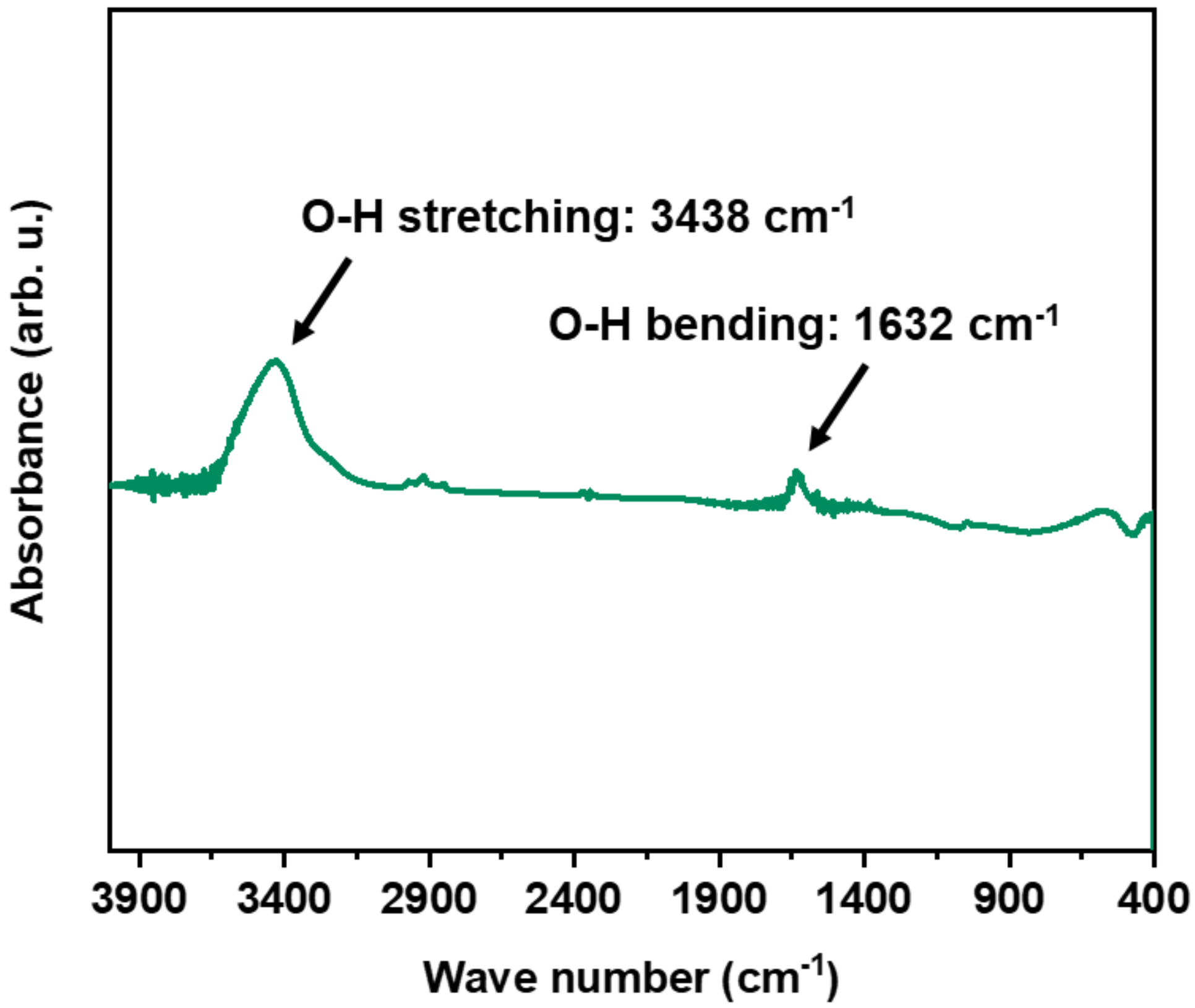

Figure S8. FT-IR spectrum of the $Y_{2.1}Te_3$ sample after complete decomposition. The peaks at 3438 cm^{-1} and 1632 cm^{-1} correspond to O-H vibrations.

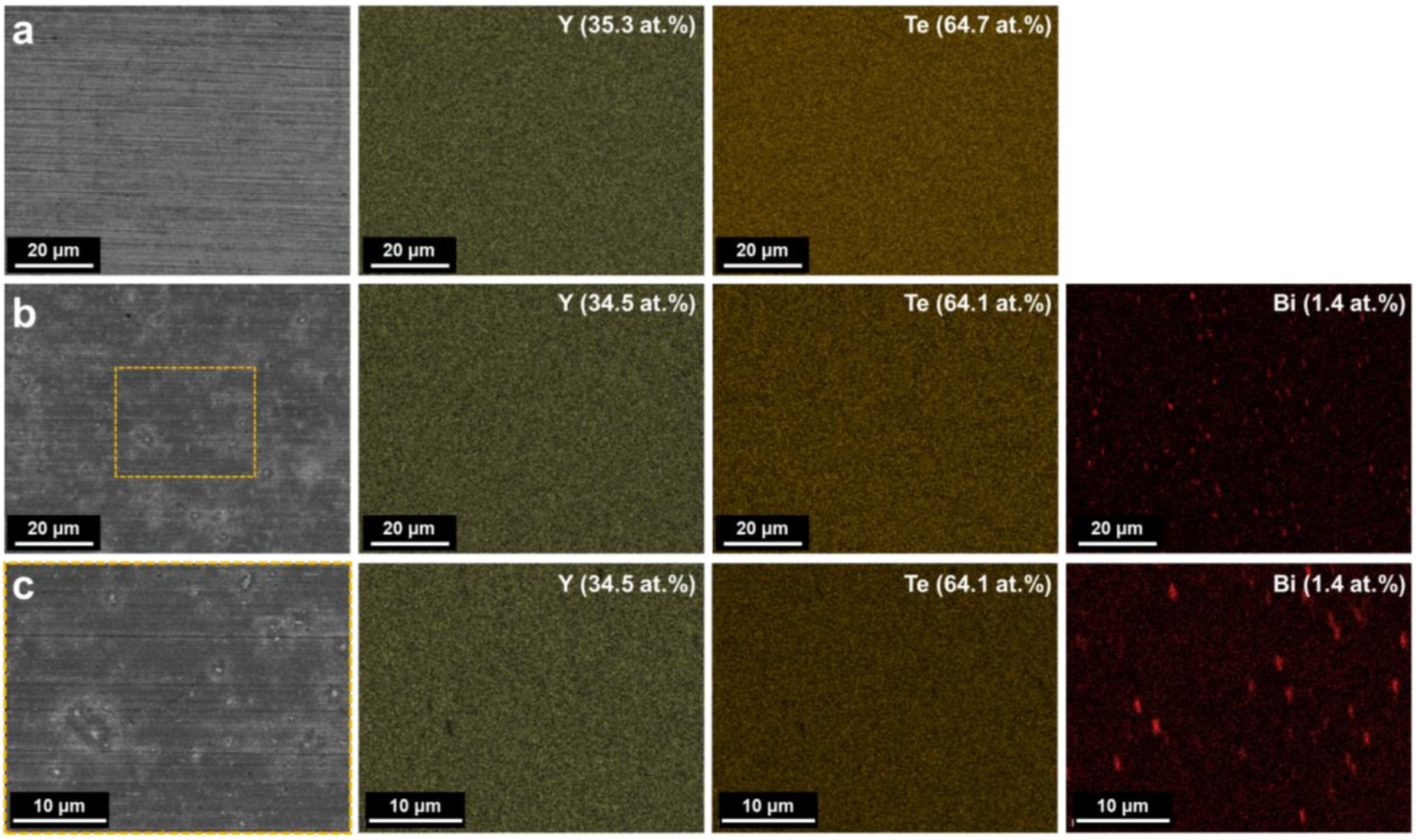

Figure S9. SEM-EDS results of as-prepared samples. Surface images and corresponding EDS maps of Y, Te, and Bi for (a) $Y_{2.1}Te_3$ and (b,c) $Y_{2.1}Te_3Bi_{0.13}$, along with their compositions (unit: atomic percent). Bi precipitates on the surface were represented.

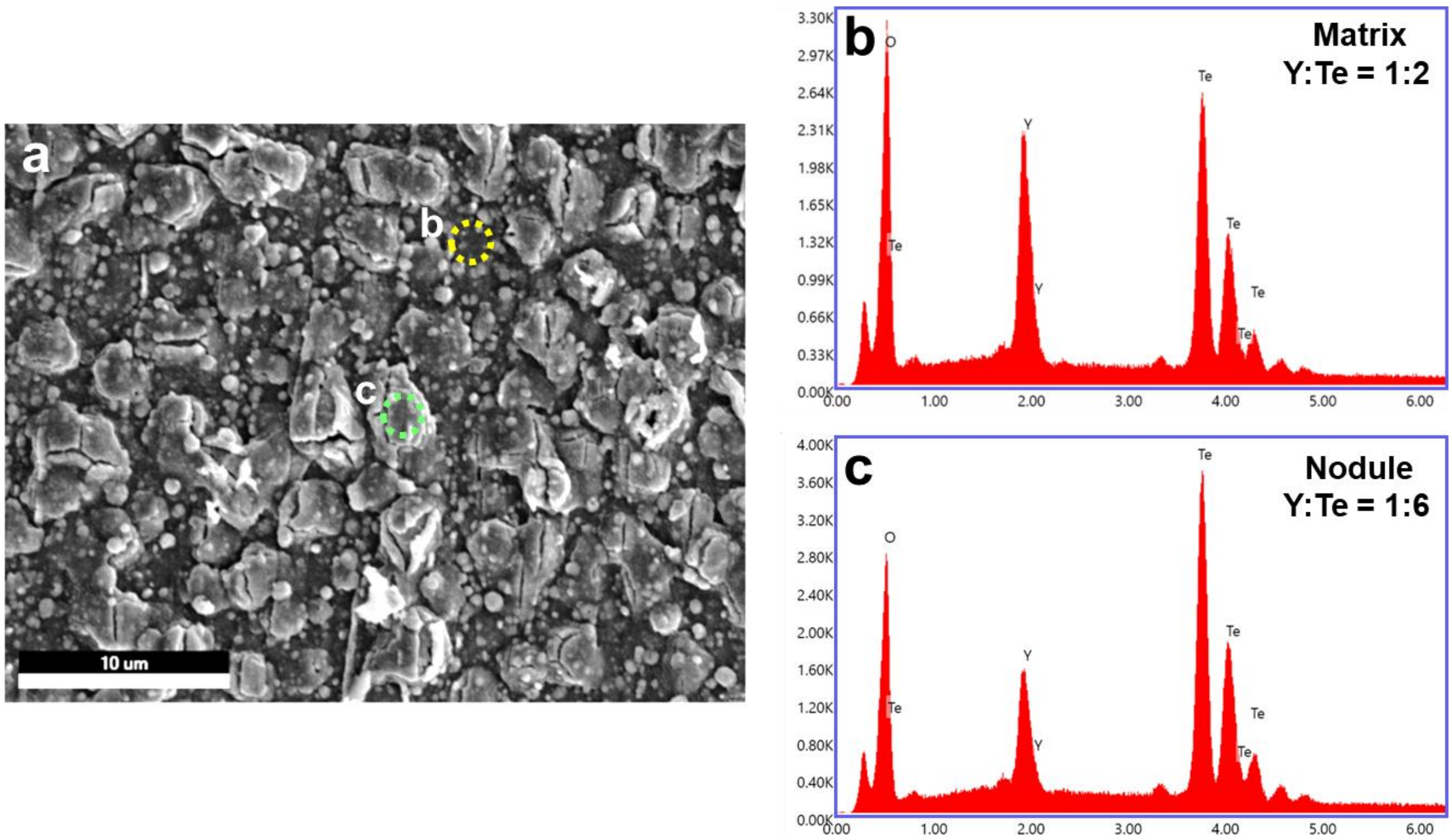

Figure S10. SEM-EDS results of 7 days air-exposed $Y_{2.1}Te_3$ samples. (a) Surface image and spot EDS spectra from (b) matrix and (c) nodule.

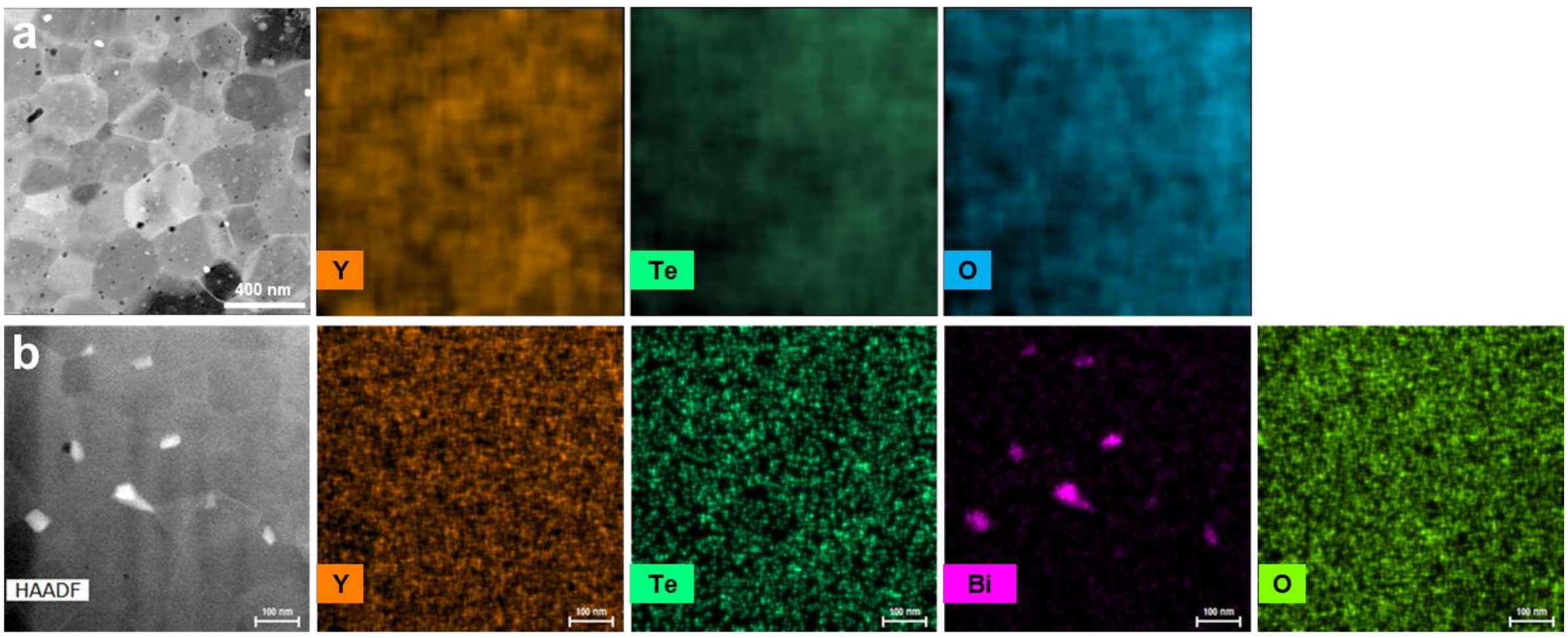

Figure S11. STEM-EDS results of the as-prepared samples. HAADF images and corresponding EDS maps of (a) $Y_{2.1}Te_3$ and (b) $Y_{2.1}Te_3Bi_{0.13}$ samples.

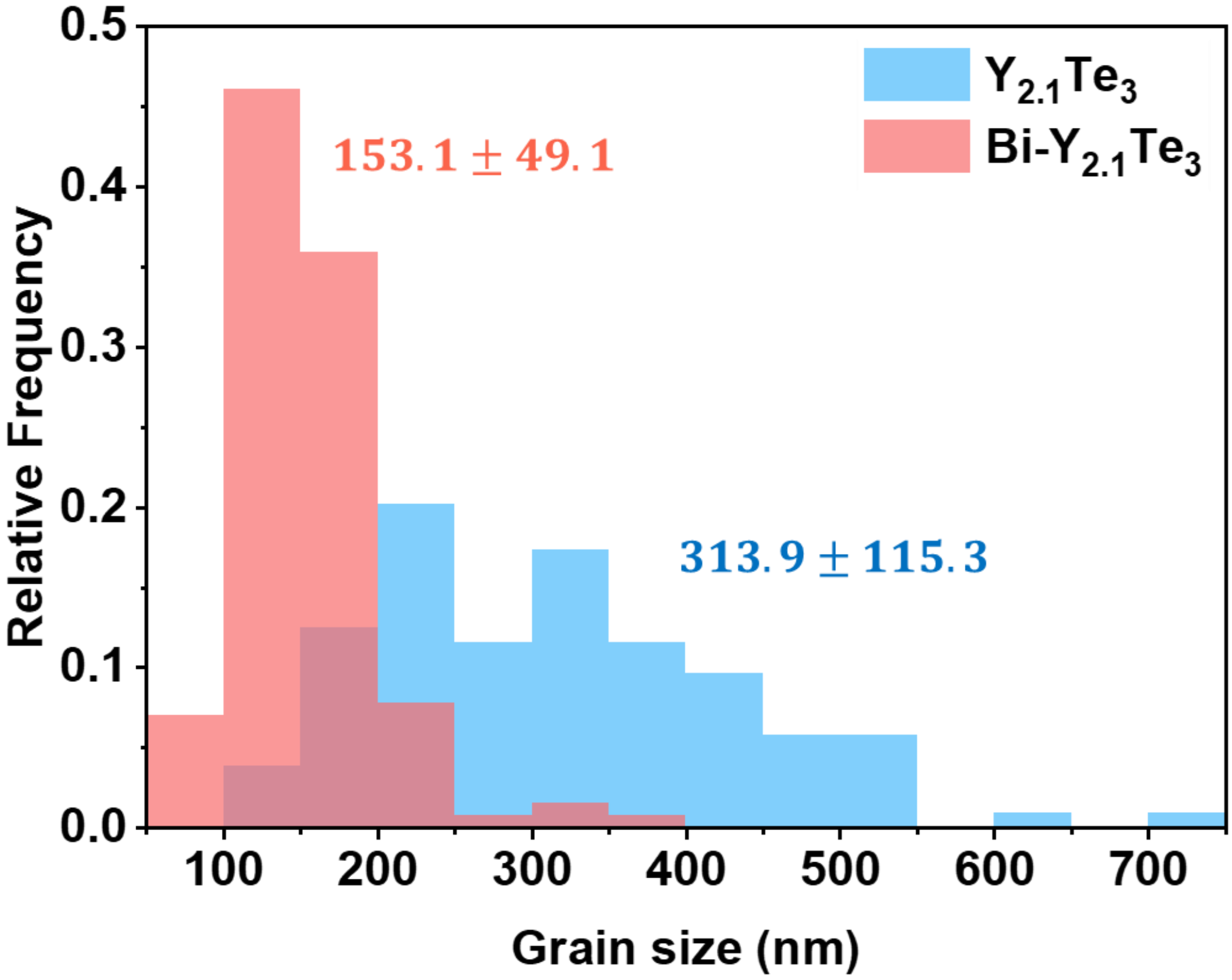

Figure S12. Grain size distribution of the as-prepared $Y_{2.1}Te_3$ ($Y_{2.1}Te_3$) and $Y_{2.1}Te_3Bi_{0.13}$ (Bi-$Y_{2.1}Te_3$) samples. Grain size histograms were obtained from HAADF-STEM images. Grain sizes are presented as mean ± standard deviation (SD). The analysis was performed using n = 104 grains for $Y_{2.1}Te_3$ and n = 128 grains for Bi-$Y_{2.1}Te_3$. The histogram bin width is 50 nm.

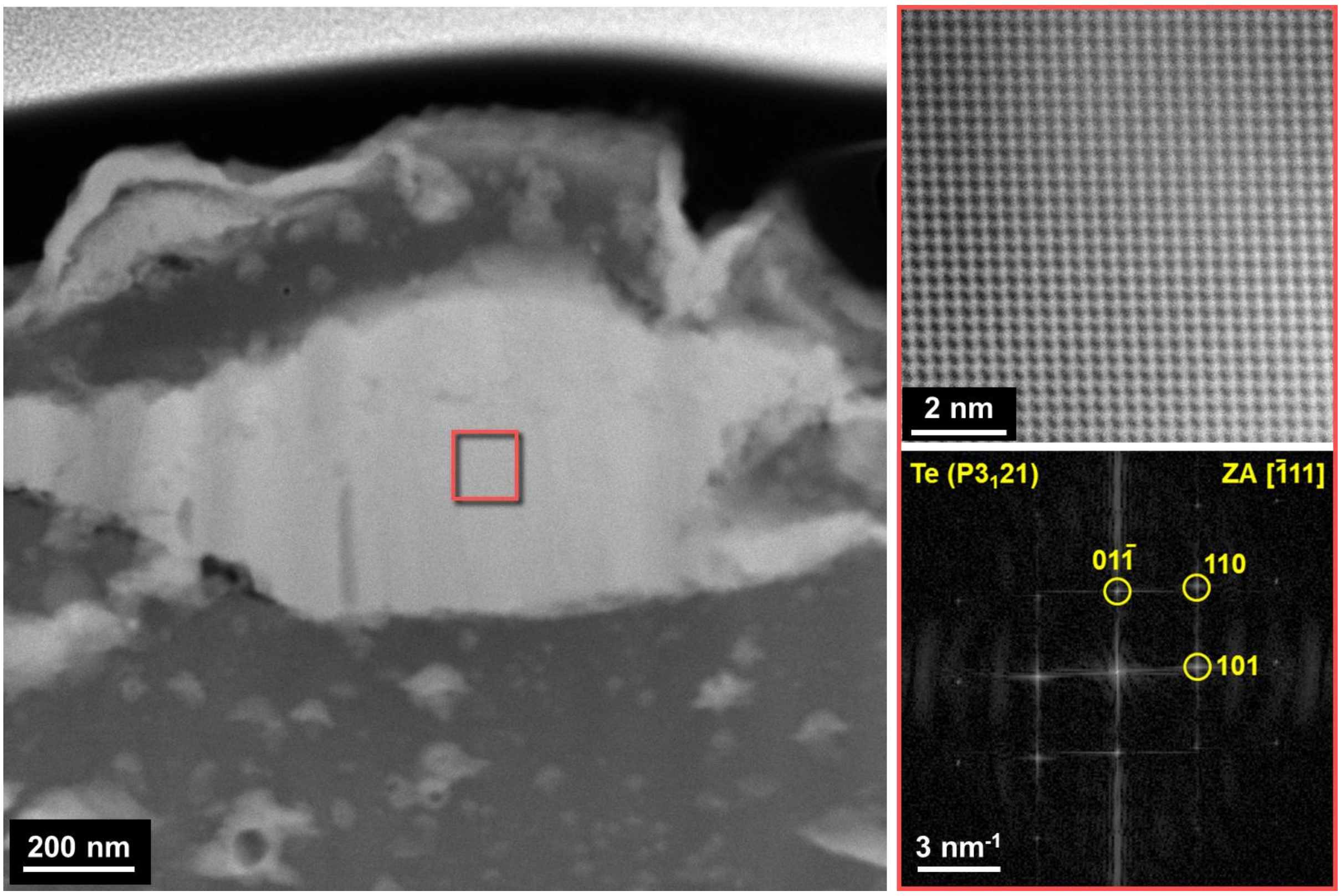

Figure S13. High-resolution STEM images of the Te nodule. HAADF image of a Te nodule on the surface of $Y_{2.1}Te_3Bi_{0.13}$, along with the corresponding atomic-resolution image and fast Fourier transform (FFT) pattern. The FFT pattern confirms that the Te nodules consist of pure elemental Te.

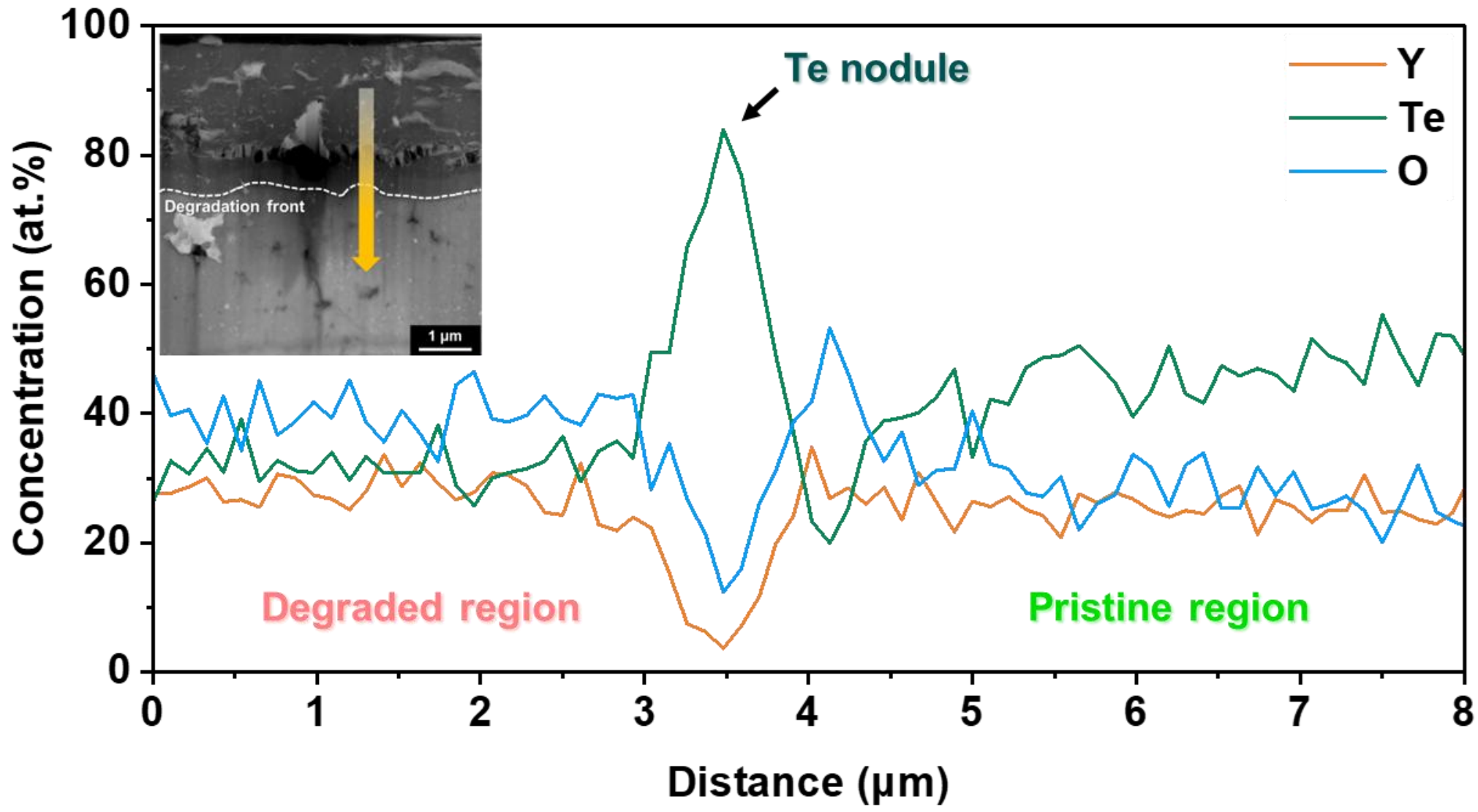

Figure S14. EDS line scan across the degradation front of the air-exposed Bi-$Y_{2.1}Te_3$ sample. The inset shows a cross-sectional HAADF-STEM image where the degradation front is indicated by the white dashed line. The yellow arrow marks the position and direction of the line scan, which traverses from the degraded surface region (left) through the degradation front into the pristine region (right). The Bi signal is not shown because of its low concentration and limited counting statistics.

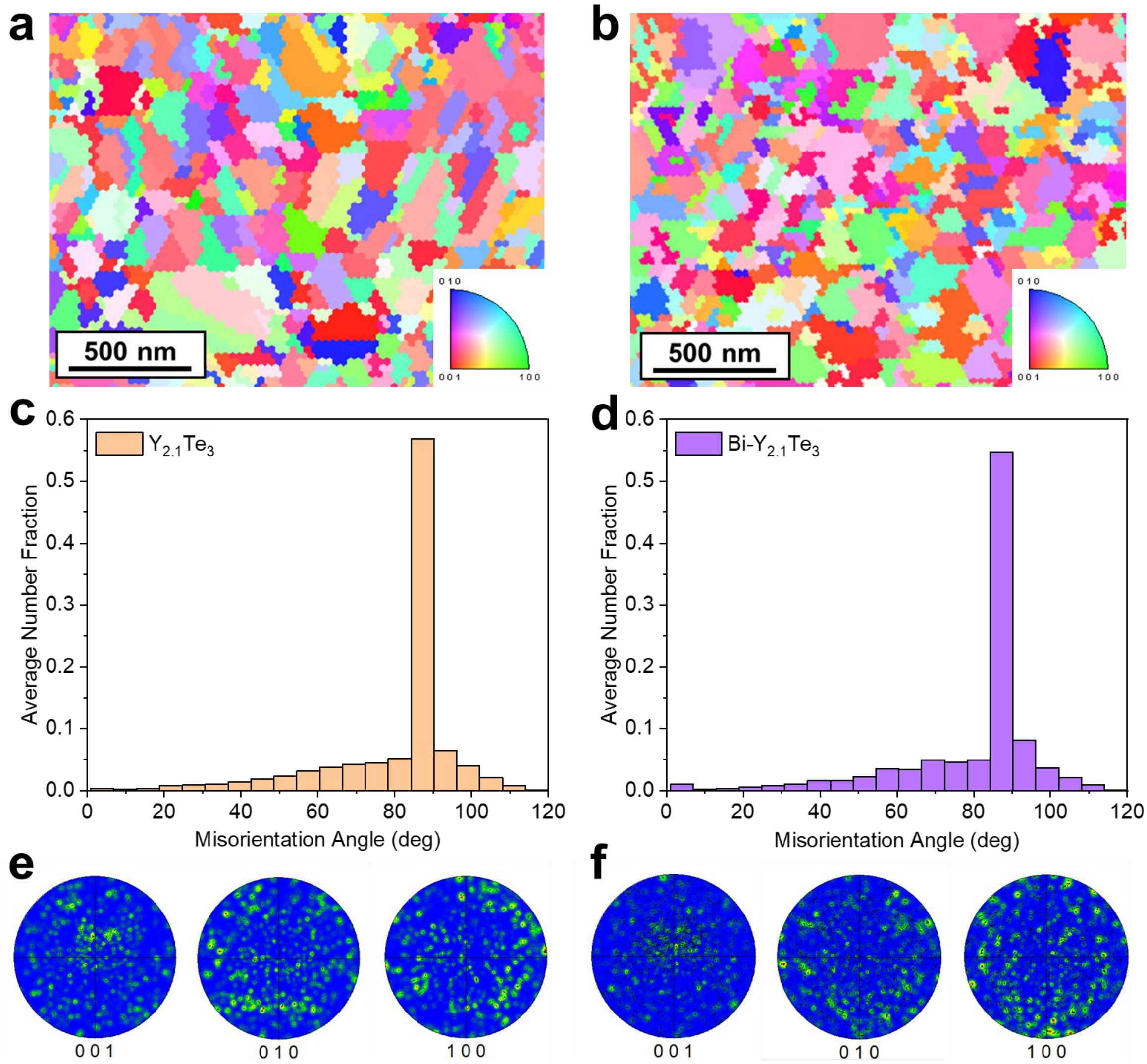

Figure S15. TKD results of $Y_{2.1}Te_3$ and Bi-$Y_{2.1}Te_3$. (a,b) Inverse pole figure maps of $Y_{2.1}Te_3$ and Bi-$Y_{2.1}Te_3$, respectively. Misorientation angle distributions for (c) $Y_{2.1}Te_3$ and (d) Bi-$Y_{2.1}Te_3$, both showing a dominant number of misorientation angles at ≈90°. The distributions were obtained from $n = 3$ independent TKD scans for $Y_{2.1}Te_3$ and $n = 4$ independent TKD scans for Bi-$Y_{2.1}Te_3$. Pole figures corresponding to {001}, {010}, and {100} planes for (e) $Y_{2.1}Te_3$ and (f) Bi-$Y_{2.1}Te_3$ reveal a weak preferred texture along the {001} direction, with no noticeable change upon Bi addition.

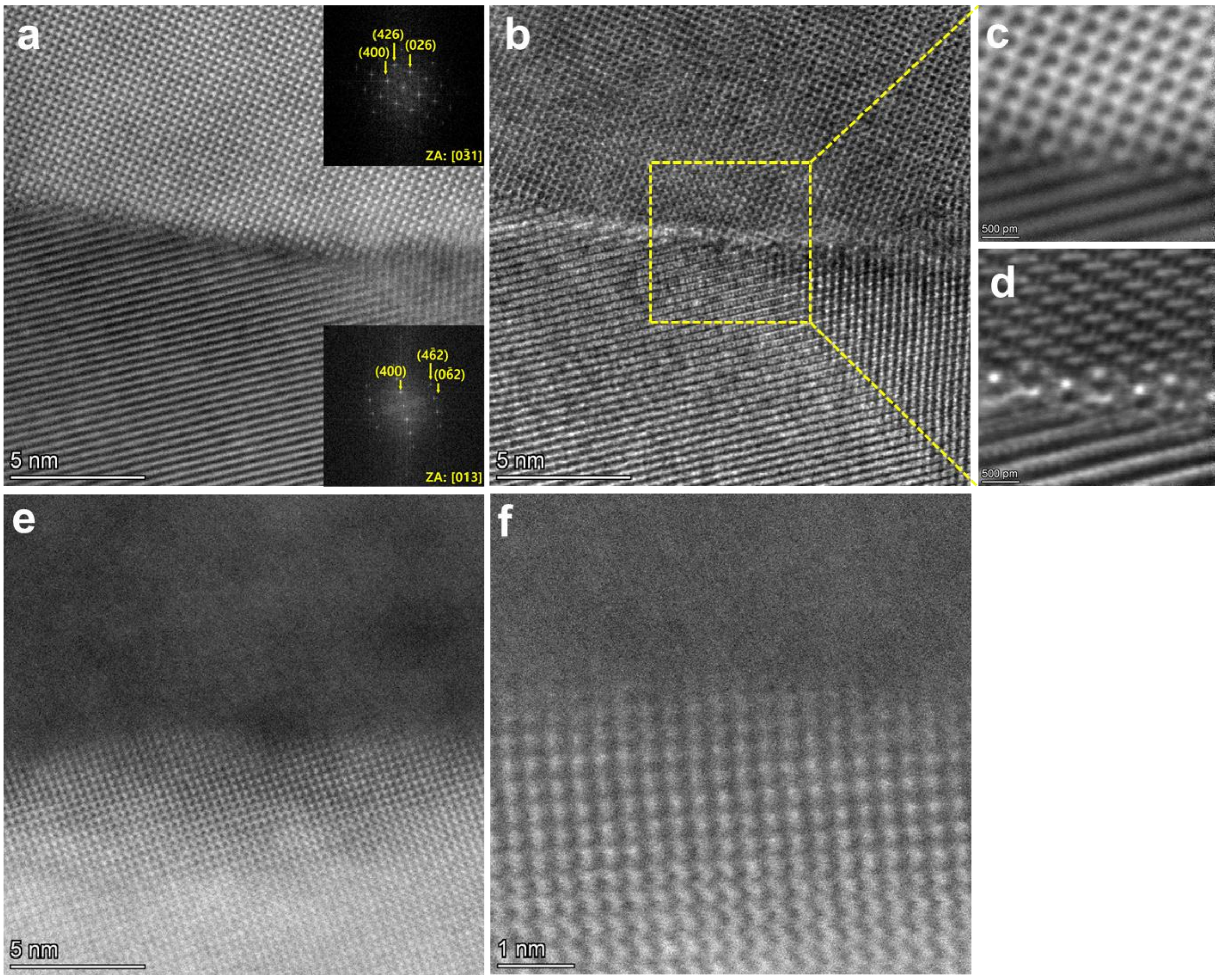

Figure S16. High-resolution STEM images of grain boundaries. (a–d) HAADF and BF images of the grain boundary with <0$\bar{3}$1>//<013> relation in $Y_{2.1}Te_3Bi_{0.13}$. Insets in (a) show the fast Fourier transform (FFT) pattern of the top and bottom grains, respectively. (c, d) magnified HAADF and BF images of the dotted region in (b), respectively. (e, f) HAADF images of $Y_{2.1}Te_3$.

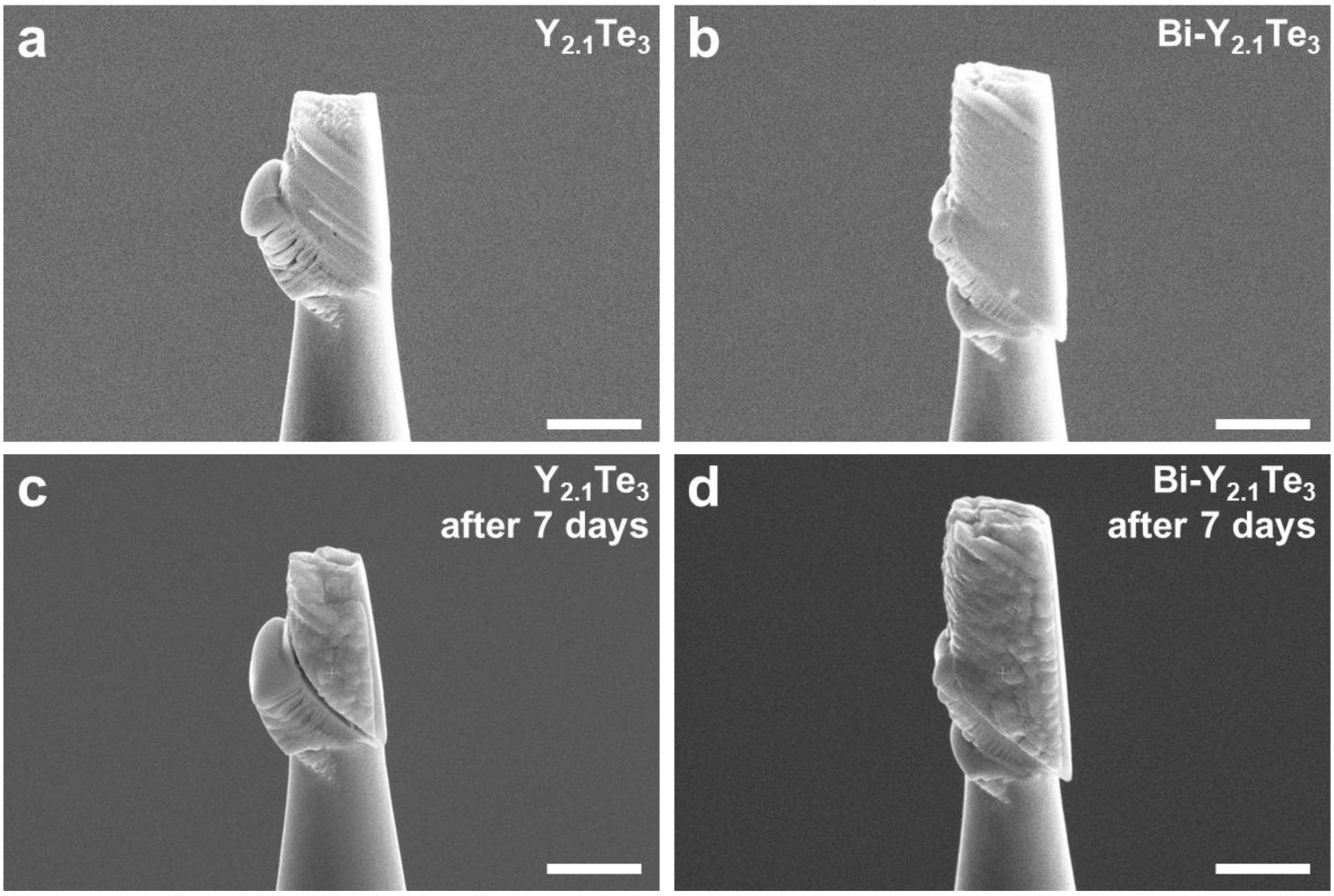

Figure S17. SEM images of the APT specimens before and after 7 days of air exposure. (a, b) As-prepared APT specimens of (a) $Y_{2.1}Te_3$ ($Y_{2.1}Te_3$) and (b) $Y_{2.1}Te_3Bi_{0.13}$ (Bi-$Y_{2.1}Te_3$) attached to Si microposts, respectively. (c, d) APT specimens of (c) $Y_{2.1}Te_3$ and (d) Bi-$Y_{2.1}Te_3$ after 7 days of air exposure, respectively. All scale bars are 2 μm.

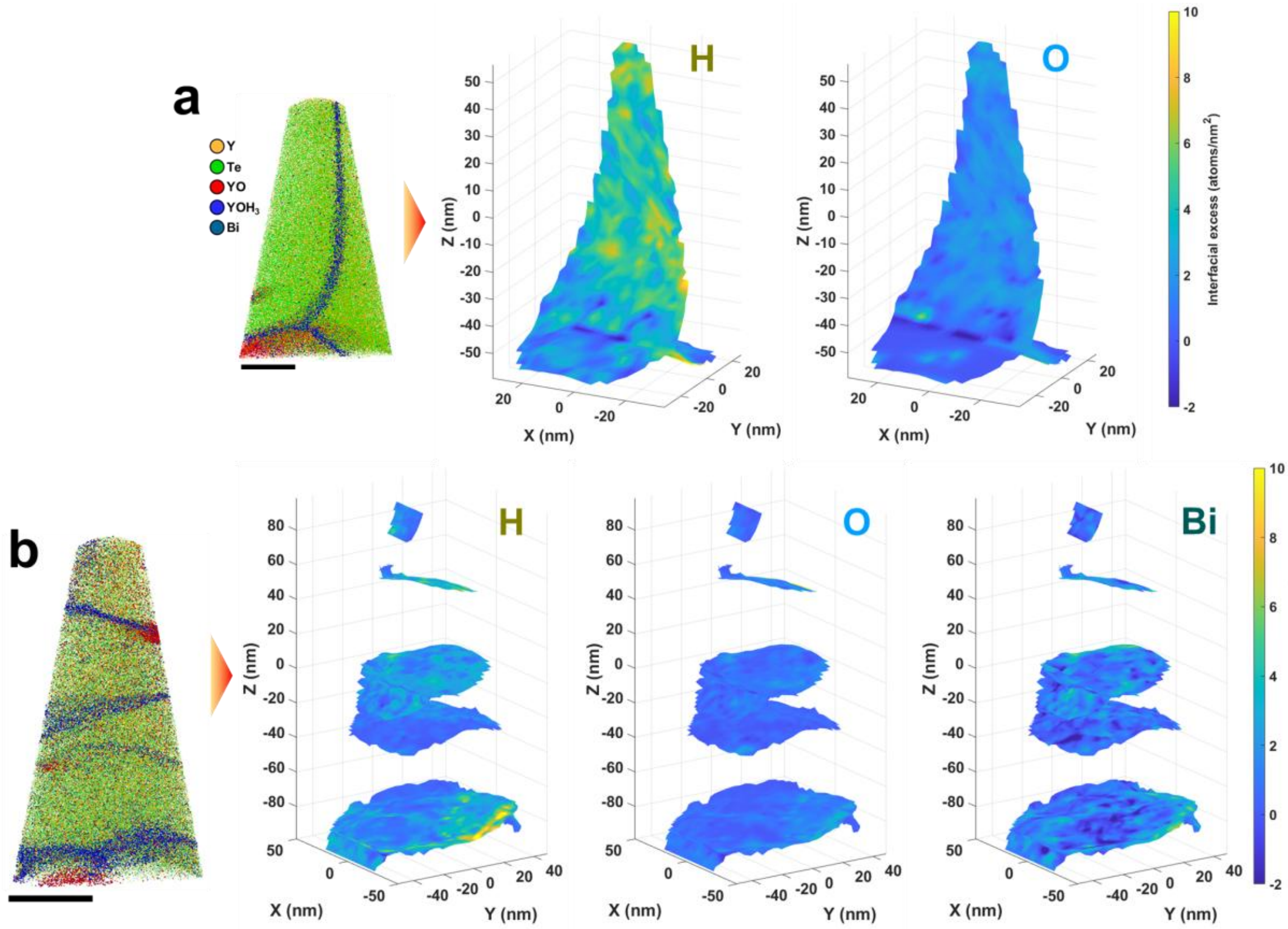

Figure S18. Local interfacial excess mapping of grain boundaries (GBs) for the as-prepared specimens. APT results of as-prepared (a) $Y_{2.1}Te_3$ ($Y_{2.1}Te_3$) and (b) $Y_{2.1}Te_3Bi_{0.13}$ (Bi-$Y_{2.1}Te_3$) with interfacial excess maps of H, O, and Bi for the GB planes detected in the 3D atom maps. Scale bars are 50 nm for (a) and 100 nm for (b).

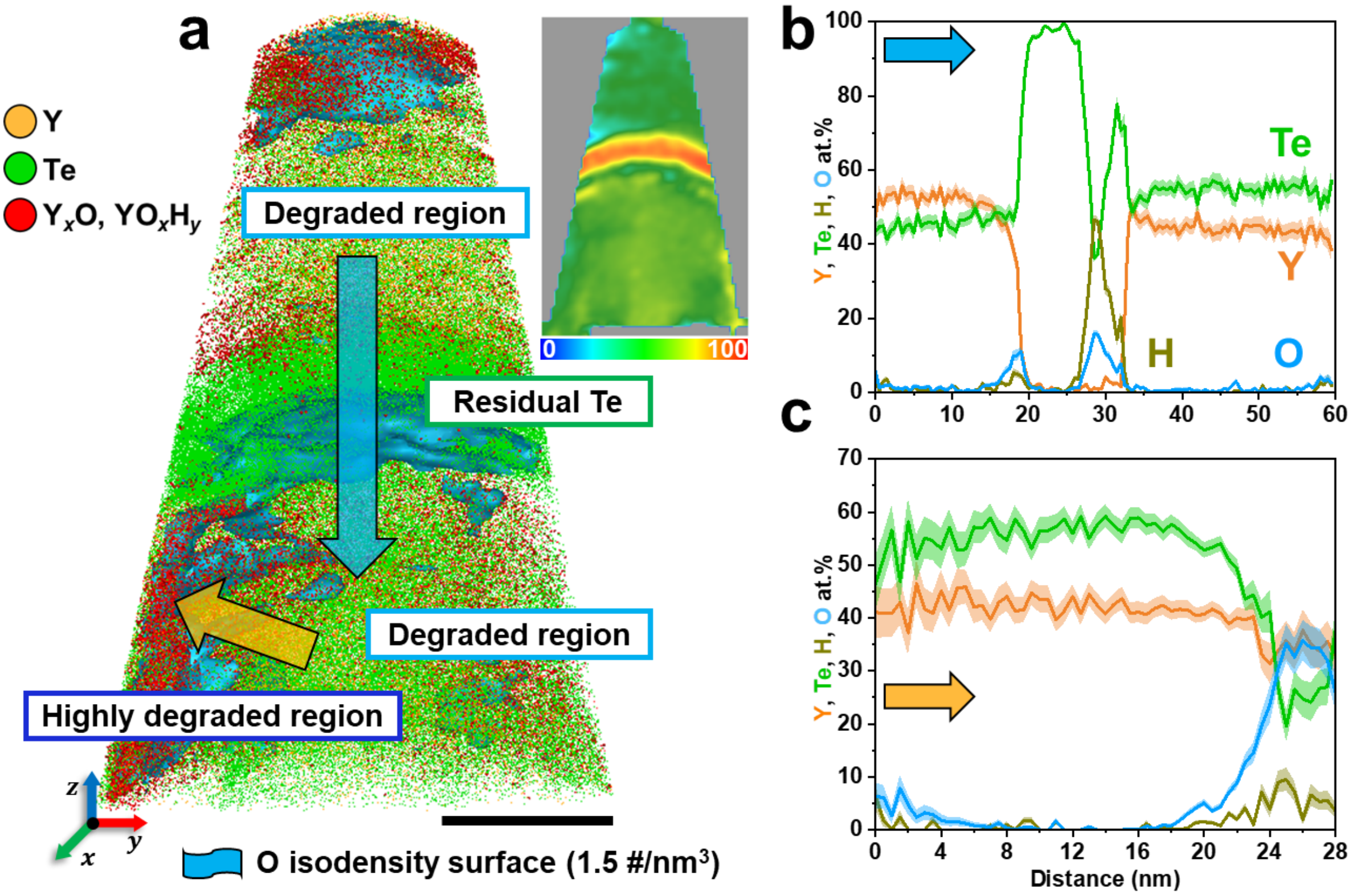

Figure S19. APT analysis of the air-exposed $Y_{2.1}Te_3$ for 7 days with the residual Te. (a) APT reconstruction of the air-exposed $Y_{2.1}Te_3$ for 7 days, showing residual Te, degraded and pristine regions with the isodensity surface of O (1.5 atoms/nm^3). Inset shows the 2D concentration plot of Te (unit: atomic percent). (b) 1D concentration profile (⌀10 nm) along the vertical direction of the atom map, following the cyan arrow indicated in (a). (c) 1D concentration profile along the orange arrow indicated in (a), representing the interface between degraded and pristine regions. Data in (b,c) are presented as bin-averaged concentration values, and the shaded regions indicate ±1 sigma statistical counting error. Scale bar is 20 nm.

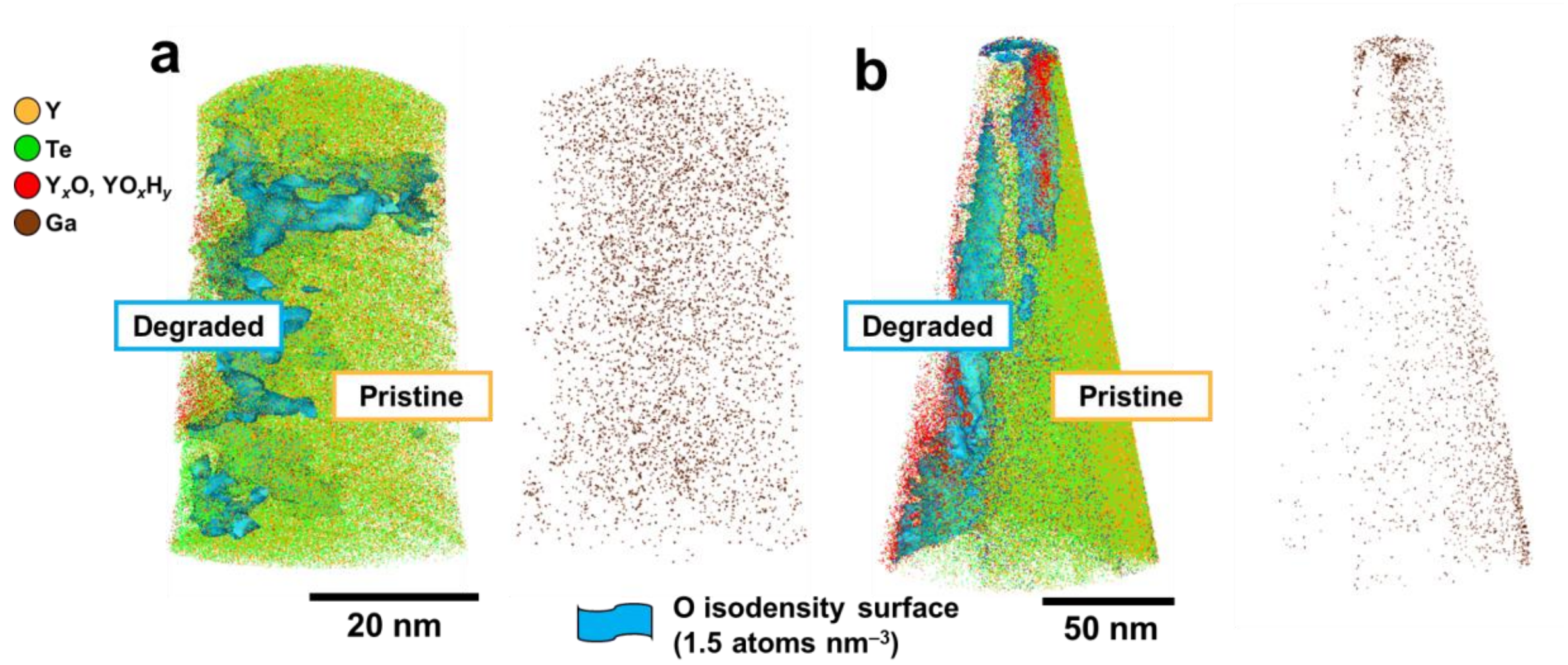

Figure S20. Spatial distribution of Ga in air-exposed APT specimens. (a, b) APT reconstructions of air-exposed (a) $Y_{2.1}Te_3$ and (b) Bi-$Y_{2.1}Te_3$, each shown with (left) the full atomic distribution including the O isodensity surface (1.5 atoms nm^{-3}) showing the degraded and pristine regions, and (right) the corresponding Ga-only distribution. In both specimens, Ga atoms are dispersed throughout the analyzed volume without preferential accumulation at grain boundaries or in the degraded regions, indicating no spatial correlation between Ga implantation from FIB specimen preparation and the moisture-induced degradation features.

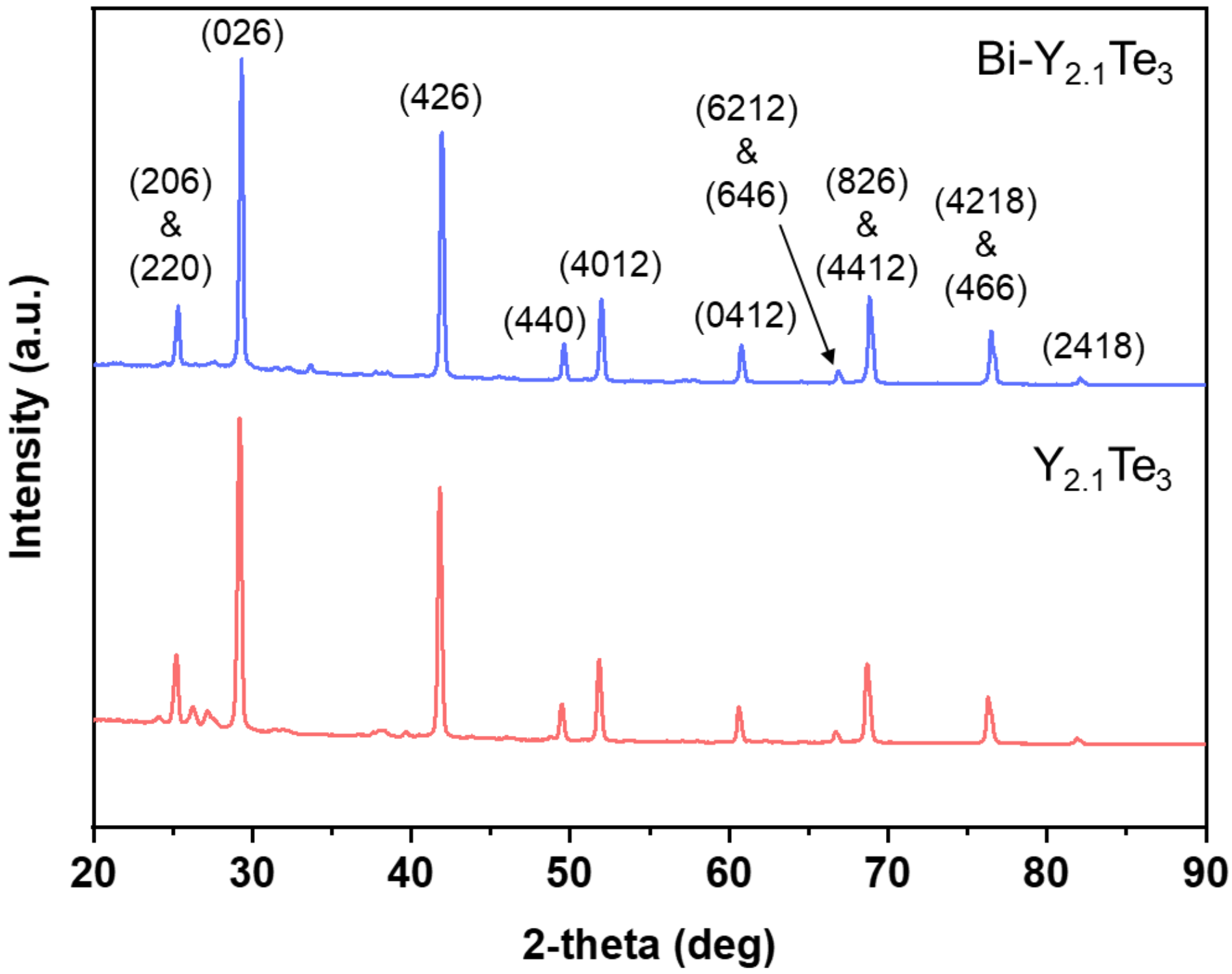

Figure S21. X-ray diffraction patterns of the as-prepared $Y_{2.1}Te_3$ and $Y_{2.1}Te_3Bi_{0.13}$ (Bi-$Y_{2.1}Te_3$) samples with indexed crystallographic planes. The prominent (026) plane indicates a preferential orientation, which was used for DFT calculations.

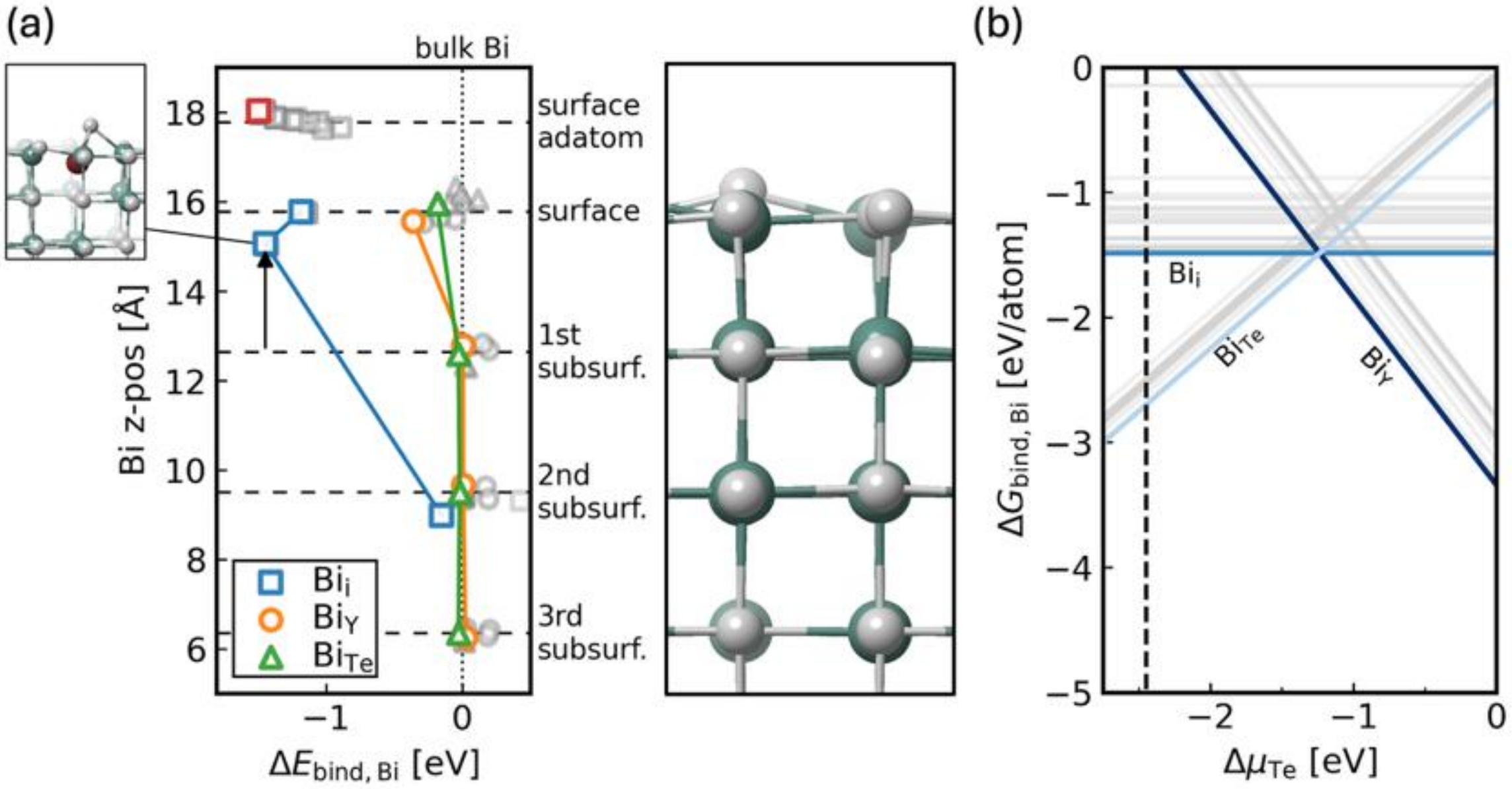

Figure S22. Binding energies of Bi defects at the surface and in the bulk. (a) Relative binding energies (ΔE_{bind}) of Bi_Y, Bi_{Te}, and Bi_i defects with respect to their bulk counterparts, plotted as a function of their vertical (z-axis) position within the Y_2Te_3(026) surface slab. Dashed horizontal lines indicate averaged atomic layer positions, and the vertical dotted lines indicate the zero reference, corresponding to the formation of each defect in the bulk. The rightmost panel illustrates the local surface structure model (cyan: Y, white: Te). (b) Gibbs free energies of binding (ΔG_{bind}) for each Bi defect as a function of Te chemical potential ($\Delta\mu_{Te}$).

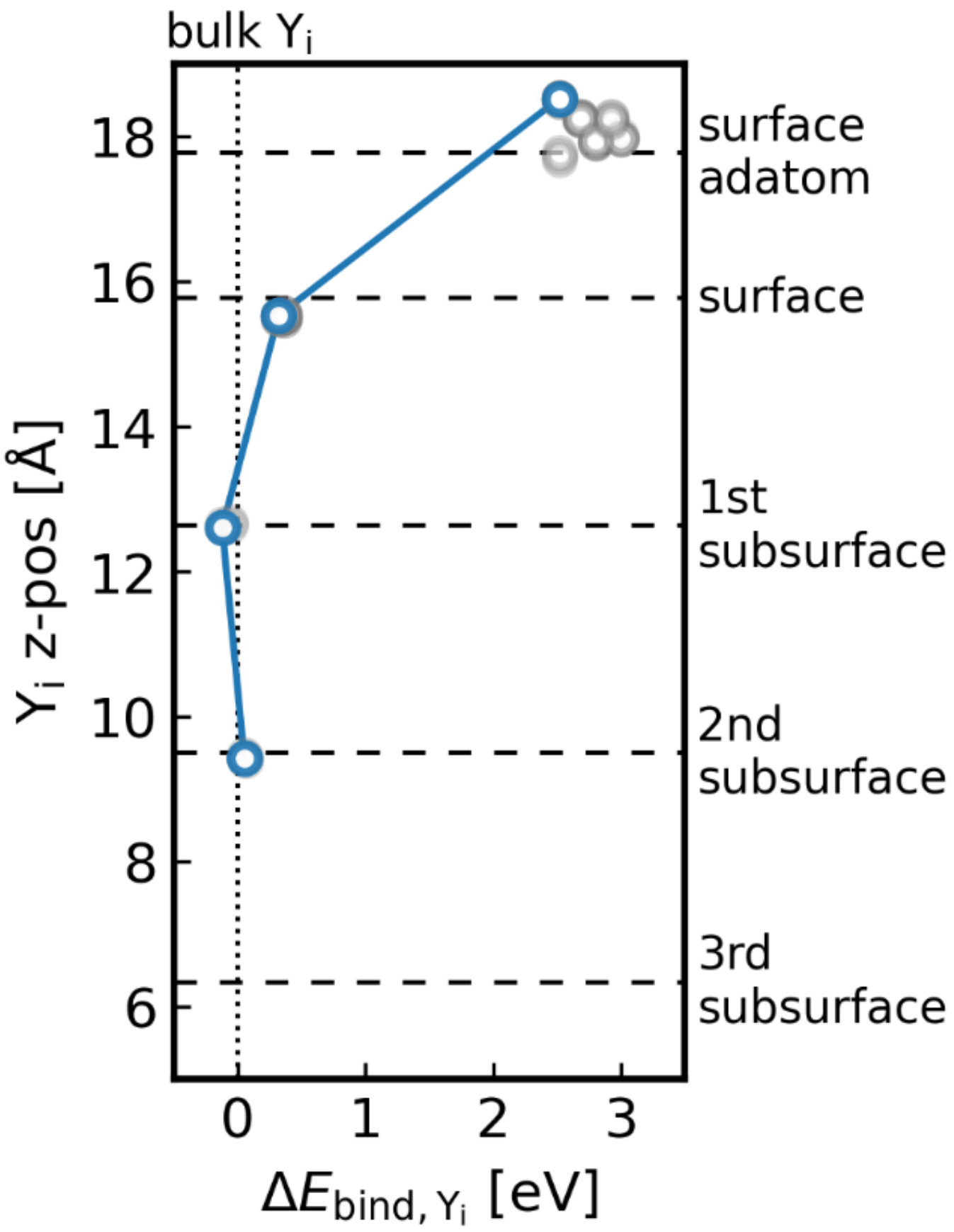

Figure S23. Relative binding energies (ΔE_{bind}) of interstitial Y (Y_i) as a function of depth from the Y_2Te_3(026) surface, shown for comparison with Bi_i in Figure 8a.

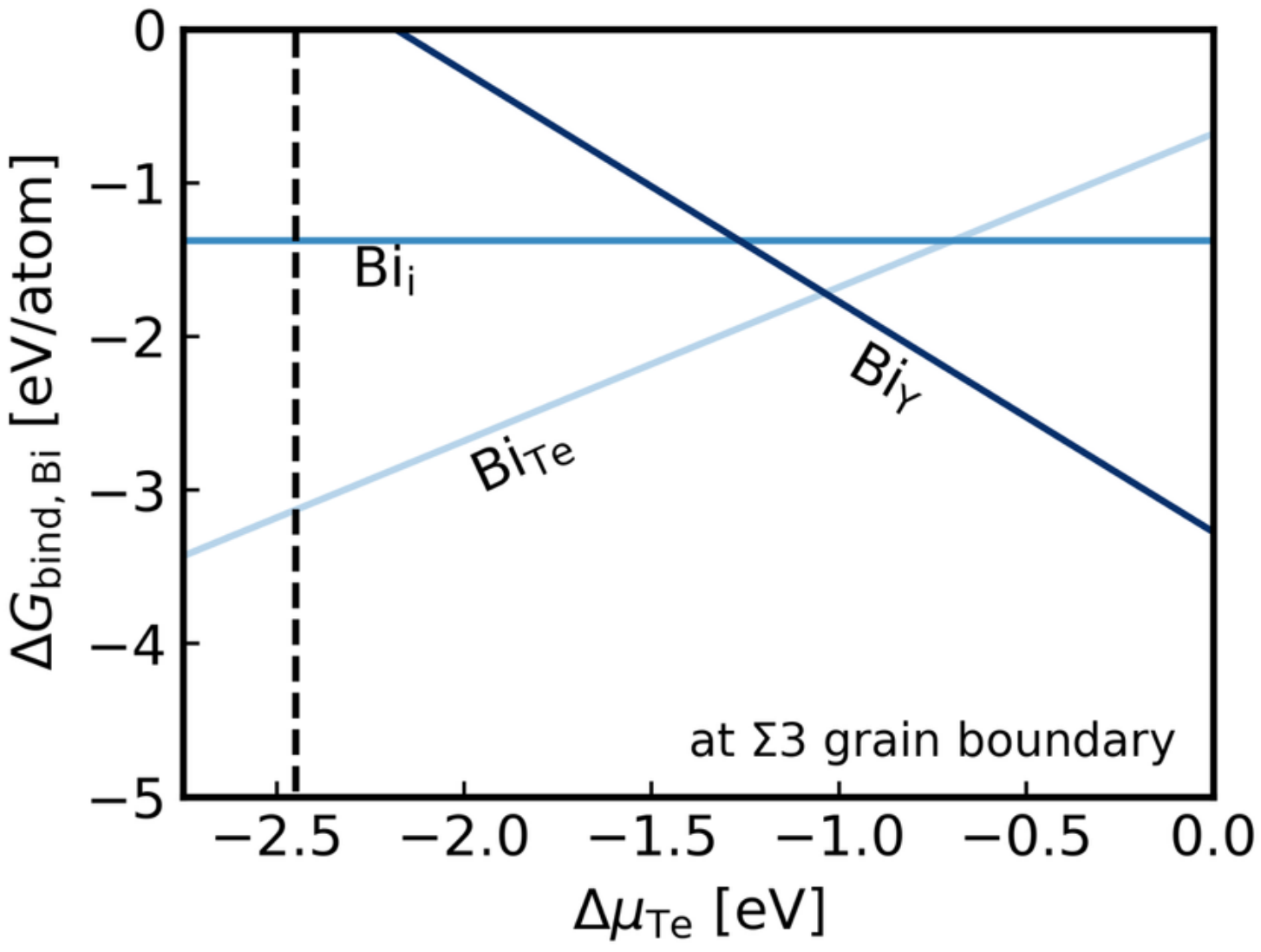

Figure S24. Gibbs free energies of binding (ΔG_{bind}) for each Bi defect as a function of Te chemical potential ($\Delta\mu_{\text{Te}}$) at the Σ3 grain boundary.

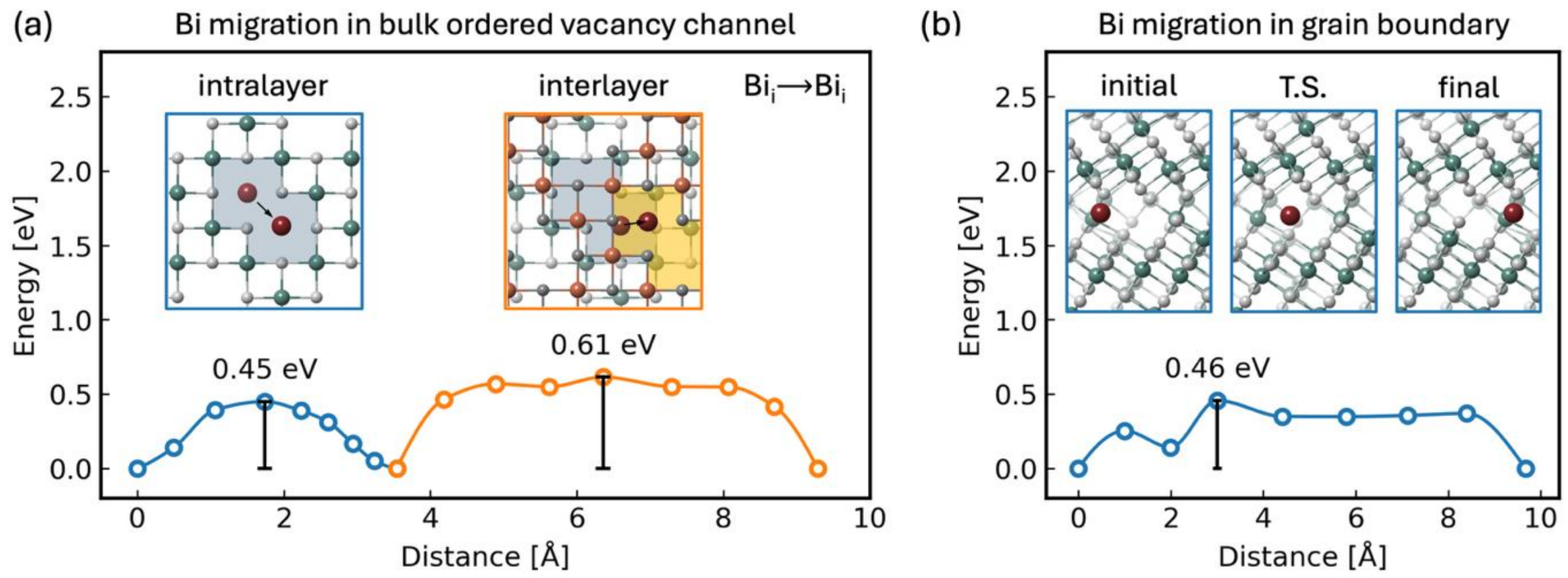

Figure S25. Diffusion energy barriers for Bi migration in bulk and grain-boundary (GB) regions of Y_2Te_3. (a) $Bi_i \rightarrow Bi_i$ diffusion along two crystallographically distinct interstitial pathways in the bulk ordered vacancy channel: intralayer diffusion within the same vacancy channel layer (blue) and interlayer diffusion between adjacent layers (orange). Both migration steps are involved in long-range Bi transport through the bulk interstitial network. Insets show schematic illustrations of the corresponding migration mechanisms. (b) $Bi_i \rightarrow Bi_i$ diffusion along the GB channel, showing a migration barrier comparable to that of bulk diffusion.

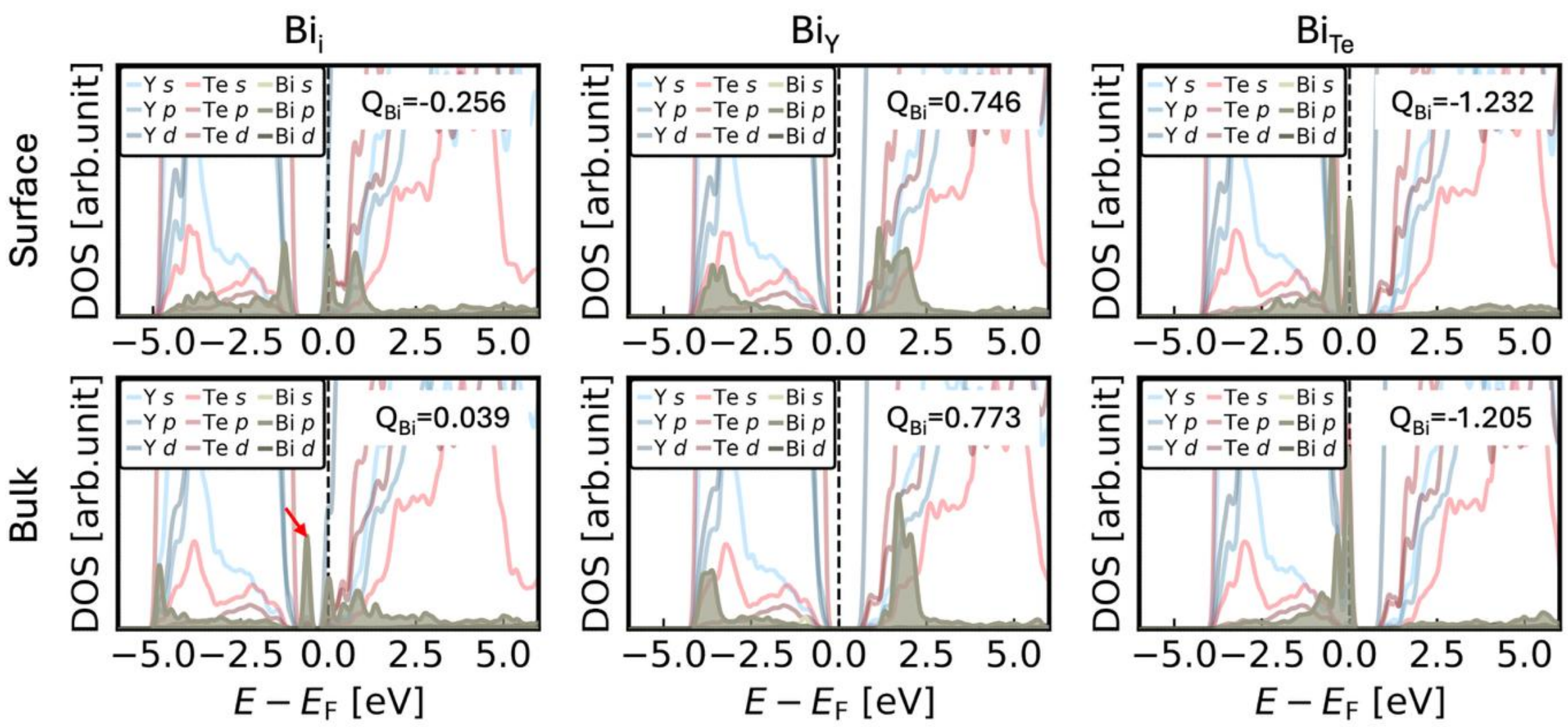

Figure S26. Projected DOS of Bi_i (left), Bi_Y (center), and Bi_{Te} (right) at the Y_2Te_3(026) surface (top row) and the deepest bulk-like subsurface layer (bottom row). Bi orbital contributions are highlighted. Corresponding Bader charges of Bi dopants are indicated within each plot.

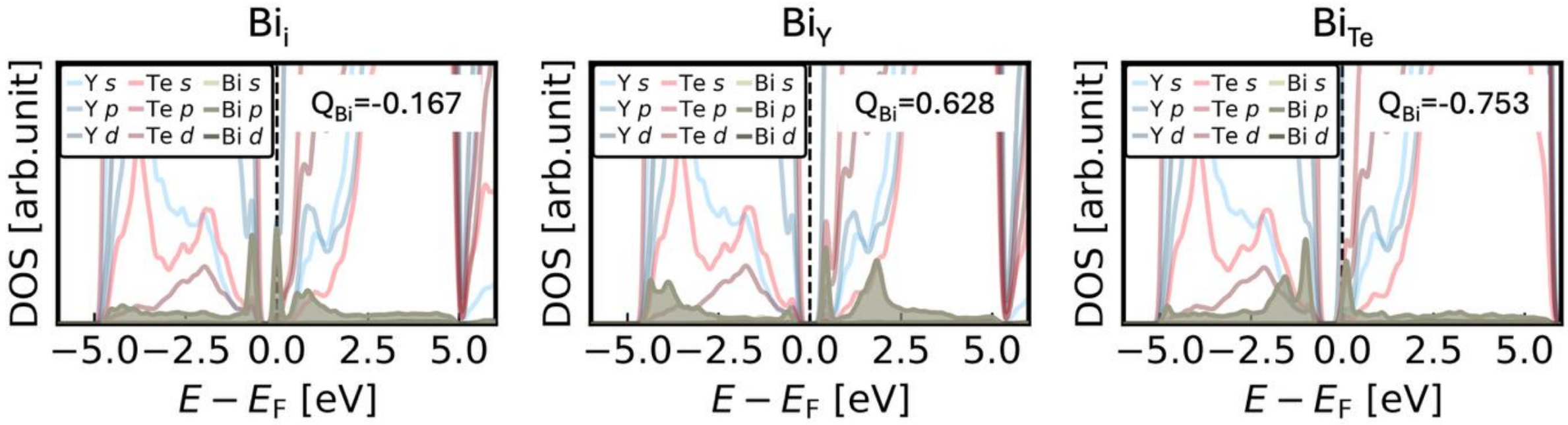

Figure S27. Projected DOS of Bi_i (left), Bi_Y (center), and Bi_{Te} (right) at the Σ3 grain boundary. Bi orbital contributions are highlighted, and corresponding Bader charges of Bi dopants are indicated within each plot.

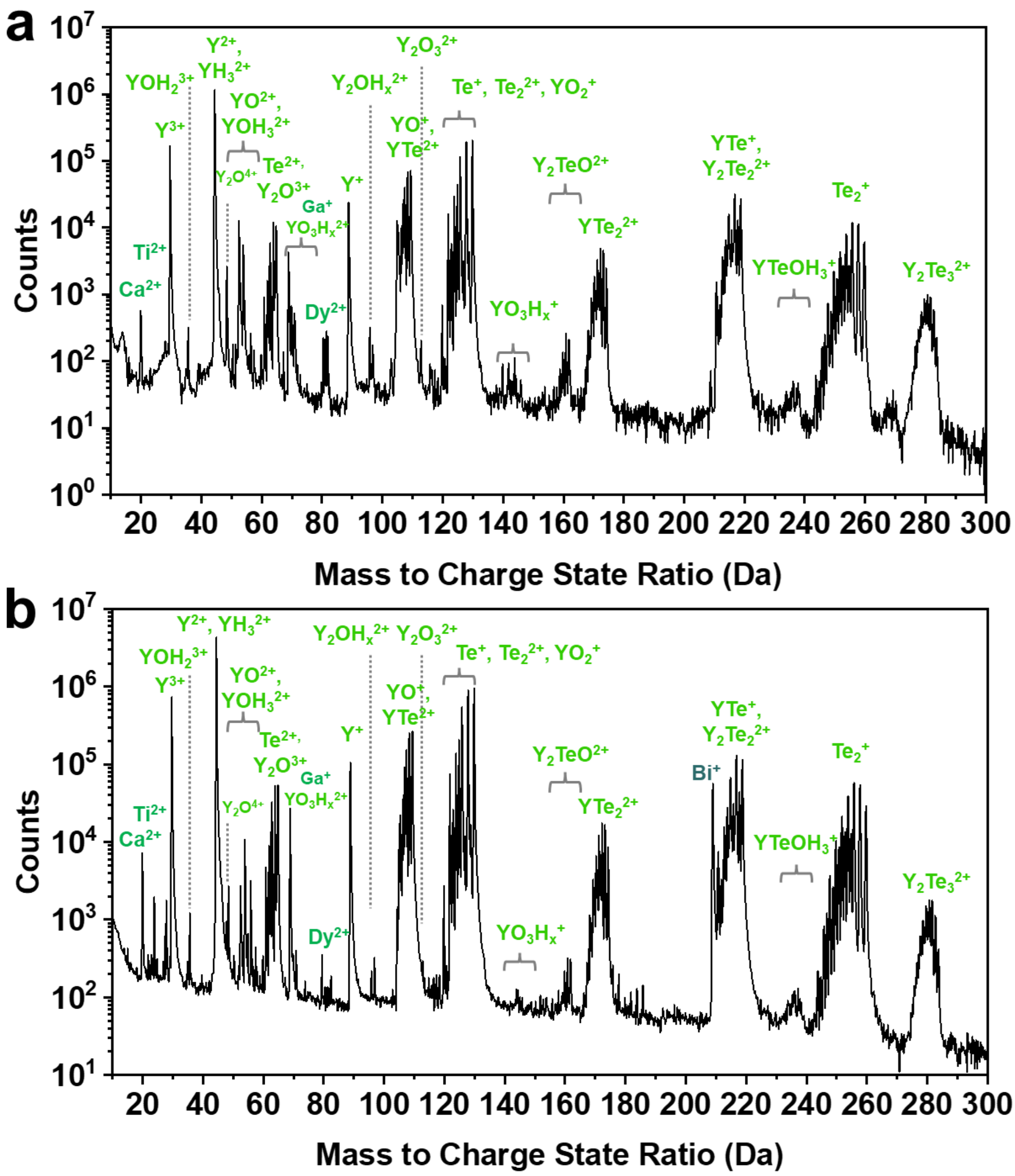

Figure S28. APT mass spectra of the as-prepared (a) $Y_{2.1}Te_3$ and (b) Bi-$Y_{2.1}Te_3$. Major matrix and dopant-related peaks are highlighted in green, whereas minor trace impurity or preparation-related peaks, including Ca, Ti, Dy, and Ga, are highlighted in red. Ca and Dy are likely associated with trace impurities in the Y raw material [Ref], while Ga is attributed to FIB preparation.

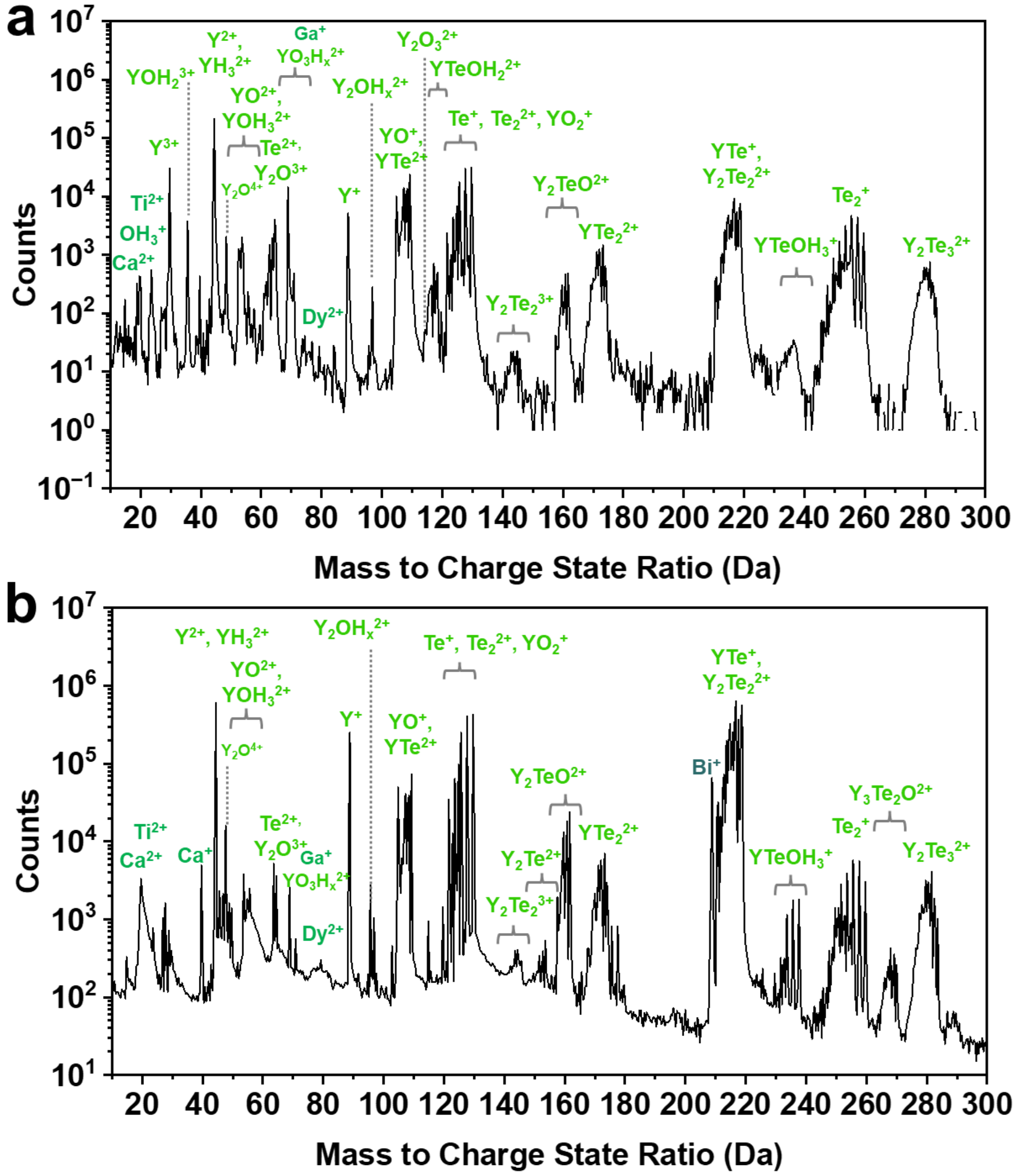

Figure S29. APT mass spectra of the air-exposed (a) $Y_{2.1}Te_3$ and (b) Bi-$Y_{2.1}Te_3$ for 7 days. Major matrix and dopant-related peaks are highlighted in green, whereas minor trace impurity or preparation-related peaks, including Ca, Ti, Dy, and Ga, are highlighted in red.

Table S1. Calculated volumetric and mass changes associated with moisture-induced degradation reactions of Y_2Te_3 for degradation reactions (1) to (3) described in the manuscript.

Reaction	Volume before degradation (cm^3/mol)	Volume after degradation (cm^3/mol)	Volume change (cm^3/mol)	Relative volume change (%)	Mass change (g/mol)
**Reaction (1)**	103.2	105.7	2.5	2.4	34
**Reaction (2)**	103.2	114.4	11.2	10.8	66
**Reaction (3)**	103.2	136.4	33.2	32.2	102

* The molar volume of each compound was calculated by dividing its molar mass by the corresponding density. The densities used in these calculations are as follows: Y_2Te_3 (5.43 g/cm^3, Ref [19]), YOH (4.78 g/cm^3, average from Ref [20]), YO_2H (4.60 g/cm^3, Ref [21]), $Y(OH)_3$ (3.73 g/cm^3, Ref [21]). For volume and mass change calculations, only solid compounds were considered.

Table S2. Ionic compositions from APT mass spectra.

Ion species	As-prepared $Y_{2.1}Te_3$ (ion.%)	As-prepared Bi-$Y_{2.1}Te_3$ (ion.%)	Air-exposed $Y_{2.1}Te_3$ (ion.%)	Air-exposed Bi-$Y_{2.1}Te_3$ (ion.%)
**Y**	27.28	32.24	19.53	2.91
**Te**	36.10	36.73	27.34	22.54
**Bi**	0.00	1.02	0.00	0.55
**YTe**	23.42	20.00	29.69	50.59
**YTe_2**	1.54	0.000	1.89	4.57
**TeY_2**	0.00	0.000	0.00	0.03
**Y_2Te_2**	1.73	1.33	3.78	14.83
**Te_2Y**	0.00	1.21	0.00	0.00
**Te_2**	6.68	6.36	7.03	0.59
**Y_2Te_3**	0.66	0.26	1.44	0.44
**YO**	0.99	0.10	2.79	0.84
**Y_2O**	0.26	0.08	1.51	0.08
**Y_2O_3**	0.01	0.002	0.00	0.00
**YO_2**	0.004	0.002	0.00	0.00
**Y_2TeO**	0.07	0.02	0.46	0.00
**TeY_2O**	0.00	0.00	0.00	1.25
**Y_3Te_2O**	0.00	0.00	0.00	0.04
**Te_2Y_2O**	0.00	0.00	0.00	0.02
**YOH**	0.00	0.01	0.25	0.003
**YOH_2**	0.01	0.01	1.02	0.01
**YOH_3**	0.32	0.11	0.71	0.15
**YH_3**	0.07	0.02	0.03	0.03
**$YTeOH_2$**	0.00	0.00	0.63	0.00
**$YTeOH_3$**	0.00	0.00	0.05	0.09
**YTe_2H_3**	0.00	0.00	0.00	0.02
**YO_3H_3**	0.04	0.003	0.08	0.00
**YO_3H_5**	0.04	0.004	0.12	0.01
**OH**	0.00	0.00	0.01	0.00
**OH_2**	0.00	0.00	0.01	0.001
**OH_3**	0.00	0.00	0.08	0.00
**O**	0.00	0.002	0.01	0.00
**Ca**	0.04	0.10	0.18	0.12
**Ti**	0.05	0.06	0.17	0.25
**Dy**	0.06	0.00	0.02	0.02
**Ga**	0.63	0.31	1.20	0.04

Table S3. Decomposed atomic compositions from APT mass spectra.

Element	As-prepared $Y_{2.1}Te_3$ (at.%)	As-prepared Bi-$Y_{2.1}Te_3$ (at.%)	Air-exposed $Y_{2.1}Te_3$ (at.%)	Air-exposed Bi-$Y_{2.1}Te_3$ (at.%)
**Y**	40.85	42.37	40.45	43.49
**Te**	56.25	55.90	50.01	54.45
**Bi**	0.000	0.76	0.00	0.26
**O**	1.33	0.27	4.60	1.17
**H**	1.03	0.35	4.00	0.43
**Ca**	0.03	0.07	0.10	0.06
**Ti**	0.03	0.05	0.09	0.12
**Dy**	0.04	0.00	0.01	0.01
**Ga**	0.43	0.23	0.68	0.02

References

1. Mishin, Y. & Herzig, C. Grain boundary diffusion: recent progress and future research. *Materials Science and Engineering* **260**, 55–71 (1999).

2. Borisov, V. T., Golikov, V. M. & Scherbedinskiy, G. V. Relation between diffusion coefficients and grain boundary energy. *Phys. Met. Metall* **17**, 881–885 (1964).

3. Page, D. E., Varela, K. F., Johnson, O. K., Fullwood, D. T. & Homer, E. R. Measuring simulated hydrogen diffusion in symmetric tilt nickel grain boundaries and examining the relevance of the Borisov relationship for individual boundary diffusion. *Acta Mater.* **212**, 116882 (2021).

4. Sen, S. *et al.* Grain boundary self- and Mn impurity diffusion in equiatomic CoCrFeNi multi-principal element alloy. *Acta Mater.* **264**, 119588 (2024).

5. Schweizer, P. *et al.* Atomic scale volume and grain boundary diffusion elucidated by in situ STEM. *Nat. Commun.* **14**, 7601 (2023).

6. Toriyama, M. Y., Cheikh, D., Bux, S. K., Snyder, G. J. & Gorai, P. Y2Te3: A New n-Type Thermoelectric Material. *ACS Appl. Mater. Interfaces* **14**, 43517–43526 (2022).

7. Zhu, T. *et al.* Charting lattice thermal conductivity for inorganic crystals and discovering rare earth chalcogenides for thermoelectrics. *Energy Environ. Sci.* **14**, 3559–3566 (2021).

8. Rahman, J. U. *et al.* Grain Boundary Engineering Enhances the Thermoelectric Properties of Y2Te3. *Adv. Energy Mater.* **15**, 2404243 (2024).

9. Sawant, K. J. & Sautet, P. Modular Global Optimization using Molecular Graphs : A Case Study of ZnO/Cu Surface for Methanol Synthesis Reaction. *ChemRxiv* https://doi.org/10.26434/chemrxiv-2025-t71bx (2025) doi:10.26434/chemrxiv-2025-t71bx.

10. Batatia, I. *et al.* Cross Learning between Electronic Structure Theories for Unifying Molecular, Surface, and Inorganic Crystal Foundation Force Fields. *ArXiv* http://arxiv.org/abs/2510.25380 (2025).

11. Bengtsson, L. Dipole correction for surface supercell calculations. *Phys. Rev. B* **59**, 12301 (1999).

12. Cheng, J., Luo, J. & Yang, K. Aimsgb: An algorithm and open-source python library to generate periodic grain boundary structures. *Comput. Mater. Sci.* **155**, 92–103 (2018).

13. Ong, S. P. *et al.* Python Materials Genomics (pymatgen): A robust, open-source python library for materials analysis. *Comput. Mater. Sci.* **68**, 314–319 (2013).

14. Shen, J.-X., Horton, M. & Persson, K. A. A charge-density-based general cation insertion algorithm for generating new Li-ion cathode materials. *NPJ Comput. Mater.* **6**, 161 (2020).

15. Garrido Torres, J. A., Jennings, P. C., Hansen, M. H., Boes, J. R. & Bligaard, T. Low-scaling algorithm for nudged elastic band calculations using a surrogate machine learning model. *Phys. Rev. Lett.* **122**, 156001 (2019).

16. Koistinen, O.-P., Dagbjartsdóttir, F. B., Ásgeirsson, V., Vehtari, A. & Jónsson, H. Nudged elastic band calculations accelerated with Gaussian process regression. *J. Chem. Phys.* **147**, 15 (2017).

17. del Rio, E., Mortensen, J. J. & Jacobsen, K. W. Local Bayesian optimizer for atomic structures. *Phys. Rev. B* **100**, 104103 (2019).

18. Smidstrup, S., Pedersen, A., Stokbro, K. & Jónsson, H. Improved initial guess for minimum energy path calculations. *Journal of Chemical Physics* **140**, (2014).

19. Hinz, I. *et al. Rare Earth Elements and Tellurium*. *Sc, Y, La-Lu Rare Earth Elements: Compounds with Te, Po* (Springer, 1986).

20. Pishtshev, A., Strugovshchikov, E. & Karazhanov, S. Conceptual Design of Yttrium Oxyhydrides: Phase Diagram, Structure, and Properties. *Cryst. Growth Des.* **19**, 2574–2582 (2019).

21. Jain, A. *et al.* Commentary: The Materials Project: A materials genome approach to accelerating materials innovation. *APL Mater.* **1**, 011002 (2013).
