# Supplementary material for: Retarding moisture-induced chemical degradation of Yttrium Tellurides by tailoring grain boundary chemistry
Source: Nat Commun. 2026 Jul 25;17:9069. doi: 10.1038/s41467-026-76056-8 (PMC13519032; doi:10.1038/s41467-026-76056-8)
Supplement: Supplementary file 2 [file 41467_2026_76056_MOESM2_ESM.pdf]

Description of Additional Supplementary Files

File Name: Supplementary Data 1
Description: Atomic coordinates of optimized DFT models of Y_2Te_3 in .xyz and .txt formats.
