# Supplementary material for: Retarding moisture-induced chemical degradation of Yttrium Tellurides by tailoring grain boundary chemistry
Source: Nat Commun. 2026 Jul 25;17:9069. doi: 10.1038/s41467-026-76056-8 (PMC13519032; doi:10.1038/s41467-026-76056-8)
Supplement: Supplementary file 3 [file 41467_2026_76056_MOESM4_ESM.pdf]

nature portfolio

Peer Review File

Retarding moisture-induced chemical degradation of Yttrium Tellurides by tailoring grain boundary chemistry

Corresponding Author: Dr Kyuseon Jang

This file contains all reviewer reports in order by version, followed by all author rebuttals in order by version.

Version 0:

Reviewer comments:

Reviewer #1

(Remarks to the Author)
The chemical stability problem is important for the widespread applications of thermoelectric materials, which is less studied in previous reports. The authors have reported the enhanced thermoelectric properties and improved chemical stability by grain boundary engineering in their previous work (Adv. Energy Mater. 2024, 15, 240423). In this work, they tried to systemically elucidate the degradation mechanisms by STEM, APT and DFT studies. Indeed, they have done many systematic work, but I still have some comments.
1. Since they have published their previous work on enhancing the thermoelectric and chemical stability by adding Bi, I am not sure whether the novelty of grain boundary engineering is high enough to be published in Nature Communications.
2. There are 9 figures presented in the manuscript.Although these systematic characterizations can explain well the degradation mechanisms, the discussions and explanations are too wordy. Therfore, I am not sure whether such long and specialized presentation can draw most of the attention from the readers of Nature Communications.
3. As shown in their Figure S5, the thermoelectric properties of their Y2.1Te3Bi0.13 samples are very poor. If the thermoelectric properties of their samples are very poor, I do not know whether the study of their chemical stability is meaningful.
4. I think they should present the aging-dependent thermoelectric characterization in their main text, when considering the main applications of Y2Te3 in thermoelectrics.

Reviewer #2

(Remarks to the Author)
Dear Editors,

The he paper “Retarding moisture-induced chemical degradation of Yttrium Tellurides by tailoring grain boundary chemistry” presents an experimental study on the effect of Bi on the chemical degradation of $Y_{2.1}Te_3$. The main finding suggests that Bi segregation to grain boundaries plays a pivotal role in mitigating H_2O-induced chemical degradation.

Below, I outline several comments and reservations regarding the interpretation of the results and the conclusions, which I would like to discuss with the authors.

• Introduction:
The statement “Although some studies have begun to apply GB engineering to improve thermal stability[13,36], the potential of GB engineering in mitigating chemical degradation remains unexplored thus far” appears overly broad. While the effect of Bi grain-boundary segregation on the properties of Y_2Te_3 may indeed represent a novel contribution of the present manuscript, the general claim that “mitigating chemical degradation remains unexplored” is difficult to support. Several publications, including Zhou 2026 (DOI: 10.1039/d5ee06360d), Zheng 2021 (DOI: 10.1039/D1CS00347J), Abbey 2022 (DOI: 10.1039/D2EE02169B), and Wang 2021 (DOI: 10.30919/es8d513), together with the papers already cited in the manuscript, provide a foundation for understanding possible mitigation strategies based on grain boundary engineering. Therefore, I would recommend using a more precise and problem specific formulation rather than a general statement implying that this research direction has not been explored at all.

• “ Kinetic barriers for Bi…” a more usual term is “migration energy barrier”

• How was the vacancy modelled in T_2Te_3?
Was it treated as a charged defect, and if so, how was the charge state selected?
Moreover, how was this charge state incorporated into the DFT calculations?
For the other defects considered in the study, were they treated as charged or neutral defects in the calculations?

• Conclusions regarding Bi kinetics are based solely on migration barrier calculations.
However, a diffusion coefficient generally contains not only the migration energy but also a prefactor, which can also be computed within DFT.
Why was this prefactor neglected, and could the authors comment on how this omission affects the validity of conclusions drawn solely from migration-energy barriers?

• The authors consider only bulk Bi diffusion.
Nevertheless, it is well known that, in polycrystalline materials, diffusion at relatively low temperatures is typically dominated by grain-boundary (GB) diffusion, whereas bulk diffusion becomes the dominant mechanism only at sufficiently high temperatures.
Could the authors comment on the relevance and applicability of their conclusions, given that they are based exclusively on bulk-diffusion calculations?

• Please provide more complete computational details for the CI-NEB calculations.
Additional information is needed to ensure reproducibility.
Specifically, please include the number of images used and provide an illustration of the selected atomic migration pathways (in at least one additional projection).
Such visualization also requires justification, as multiple diffusion pathways or mechanisms may be possible.
Why was only a single atomic migration mechanism considered?

• Discussion:
The statement “To the best of our knowledge, this work represents the first demonstration of enhanced chemical durability in thermoelectric materials through GB segregation” is not fully convincing. Based on both the DFT and experimental results presented, the evidence does not yet appear sufficient to attribute the mitigation of material degradation primarily to Bi grain boundary (GB) segregation.

1. Is there any evidence that H_2O diffusion occurs primarily along GBs?
The proposed mechanism involves GB or bulk mediated diffusion processes associated with the dissociation of H_2O into H^+ and OH^- at the surface (a step not shown in Fig. 6), followed by atomic—rather than molecular—diffusion.
Do the authors have any evidence for molecular H_2O diffusion to support their conclusion, or is the mechanism assumed?
Clarification on the dominant diffusing species (molecular vs. dissociated species) is necessary for interpreting the results.

2. What is the possible role of Bi surface segregation?
Why is surface segregation of Bi excluded from the conclusions?
Bi enrichment at surfaces may influence oxidation resistance, adsorption energetics, or surface reaction pathways. This should be discussed explicitly.

3. Bi is generally known to reduce GB cohesion, as documented in several studies. Therefore, proposing that Bi improves resistance to chemical degradation via GB segregation appears counterintuitive.
o Did the authors evaluate the effect of Bi on the GB work of separation in their DFT calculations?
o How does Bi segregation modify GB decohesion resistance?
o How does this behavior change in the presence of H and O species?

Such DFT based insights—if available—would provide a stronger physical basis for the proposed hypothesis.

Alternatively, a micro pillar bending test on GBs with and without Bi segregation, before and after oxidation, could help validate the mechanical implications of Bi segregation experimentally.

Reviewer #3

(Remarks to the Author)
The manuscript titled “Retarding moisture-induced chemical degradation of Yttrium Tellurides by tailoring grain boundary chemistry” has been reviewed. This study systematically investigates the reason behind an enhanced mechanical and chemical stability of Y2Te3 under ambient conditions as an emerging n-type thermoelectrical material. While grain boundary (GB) engineering is used to improve thermoelectric performance, it’s role in mitigating chemical degradation remains a largely unexplored area. Using advanced characterization techniques, the authors reveal that moisture-driven degradation occurs via rapid H2O diffusion along GBs, leading to oxidation into Y-O-H phase, Te segregation into a relatively pure form, and eventual chemo-mechanical failure with increased exposure time. Remarkably, incorporating about 1at% Bi significantly retards this process by segregating at GVs, forming a chemically passivated layer that blocks water ingress. APT is used to map the compositional distribution show a reduced O and H enrichment at Ri-rich GVs, while DFT calculations indicate that Bi segregation is thermodynamically favorable. The addition of Bi not only improves chemical durability but also enhances

the thermoelectric properties. These finding establish GB segregation as a multifunctional engineering strategy for simultaneously optimizing structural, chemical, and performance stability.

The manuscript is very well written with an appropriate presentation of background and motivation followed by a logical presentation of results. I find no fatal issues with the results and the interpretation thereof. However before the manuscript should be considered for publication here or elsewhere, the authors should address the following questions, comments, or recommendations, which are presented in chronological order.
1) Page 7: Regarding the description of the use of silica gel in the text and the images in Figure 1, I assume that the silica gel is the thick black layer in the right-hand-side vile for x=0 and x=0.13. Please clarify this since silica gel is usually somewhat clear in appearance with a yellowish tint.
2) Page 9: When describing the results of Figure 2 in the text, please include mention about the lack of not seeing any Y-oxide in the XRD, stating that this is likely due to it being an amorphous phase and refer to results later in the manuscript that corroborate this. As a reader, I was left wondering about this in the back of my mind until it was addressed later (page 11).
3) Page 13: Regarding "results indicate that the overall Y content of the matrix is preserved during degradation, thus…" – this is not clear from the EDS images. Provide an EDS line scan to better support this claim.
4) Page 16: what is "ion projection microscopy". Think you mean to say Field ion microscopy which is a more common/proper description keeping with the jargon of the field.
5) Page 16: Regarding "Because of their extremely small volumes and large surface-to-volume ratios, the air-exposed specimens of both Y2.1Te3 and Bi-Y2.1Te3 rapidly degraded and showed noticeable surface transformations during exposure", please show SEM images of this "noticeable surface transformation" in the supplementary information section.
6) Please include a clearly labeled APT mass spectrum in the supplementary information section, for both samples. ALSO include a table of both the ionic and decomposed atomic compositions in the supplementary information section.
7) Since the APT samples were Ga FIB-prepared, please comment on the surface damage as indicated by implanted Ga. Is there a spatial correlation with the Ga distribution and "degraded" regions in the sample. What I'm getting at is if/how Ga (if present) may be influencing your results. Surface damage from Ga could lead to observing more water and hydrocarbons on the damaged surface from air exposure.
8) I find it surprising that you got such large amounts of data from the samples without Bi that were exposed to air. If chemo-mechanical degradation is occurring, I would expect the samples to be relatively brittle leading to unstable field evaporation and premature fracture before significant data can be collected. Please provide a comment.

Version 1:

Reviewer comments:

Reviewer #1

(Remarks to the Author)
In the revised manuscript, the authors have properly addressed my comments and made corresponding revisions. I think this revised paper can be accepted now.

Reviewer #2

(Remarks to the Author)
Dear Editors,
I have read the reports, the replies and the updated manuscript. In my opinion, the authors did a good job by replying to the most critical questions and enhancing the manuscript quality. It can be accepted for publication in the present form.

Reviewer #3

(Remarks to the Author)
I feel that the authors have sufficiently addressed my review comments. Additionally, to the best of my knowledge, the authors have also sufficiently addressed the comments from the two other reviewers

Open Access This Peer Review File is licensed under a Creative Commons Attribution 4.0 International License, which permits use, sharing, adaptation, distribution and reproduction in any medium or format, as long as you give appropriate credit to the original author(s) and the source, provide a link to the Creative Commons license, and indicate if changes were made.
In cases where reviewers are anonymous, credit should be given to 'Anonymous Referee' and the source.
The images or other third party material in this Peer Review File are included in the article's Creative Commons license, unless indicated otherwise in a credit line to the material. If material is not included in the article's Creative Commons license and your intended use is not permitted by statutory regulation or exceeds the permitted use, you will need to obtain permission directly from the copyright holder.
To view a copy of this license, visit https://creativecommons.org/licenses/by/4.0/

Response to reviewers' comments

Reviewer #1

The chemical stability problem is important for the widespread applications of thermoelectric materials, which is less studied in previous reports. The authors have reported the enhanced thermoelectric properties and improved chemical stability by grain boundary engineering in their previous work (Adv. Energy Mater. 2024, 15, 240423). In this work, they tried to systemically elucidate the degradation mechanisms by STEM, APT and DFT studies. Indeed, they have done many systematic work, but I still have some comments.

Comment 1:

"Since they have published their previous work on enhancing the thermoelectric and chemical stability by adding Bi, I am not sure whether the novelty of grain boundary engineering is high enough to be published in Nature Communications."

Response 1:

We thank the Reviewer for this important comment. We would like to respectfully note that chemical stability was not addressed in our previous publication (Rahman et al., Adv. Energy Mater. 2024, 2404243). That work focused exclusively on the role of grain boundary engineering in tuning thermoelectric transport, specifically, how excess Y and minor Bi incorporation modify charge carrier scattering at GBs to enhance the Seebeck coefficient and *zT*. Neither the chemical degradation behavior of Y_2Te_3 nor the protective mechanism of Bi was investigated or reported.

The novelty of the present manuscript lies in three distinct contributions that go beyond synthesis and thermoelectric performance:

1. Mechanistic elucidation of chemical degradation in Y_2Te_3, identifying H_2O (rather than O_2) as the primary degradation agent and grain boundaries as the dominant ingress pathway, established through controlled silica-gel experiments, humidity-resolved TGA, and direct atomic-scale evidence from STEM and APT.
2. Demonstration that GB engineering can enhance chemical durability of a thermoelectric material, achieved by Bi segregation that chemically passivates GBs against H_2O ingress. To

our knowledge, prior GB engineering studies in thermoelectrics have addressed transport properties or thermal stability, but not chemical stability.

3. A surprisingly low threshold of just ~1 at.% Bi is sufficient to suppress moisture-induced degradation, supported by quantitative APT correlation analyses and DFT calculations that establish both the thermodynamic and kinetic origins of GB segregation.

Thus, the present manuscript reveals a new function of GB engineering: suppressing moisture-induced chemical degradation. We believe this substantially expands the significance of GB engineering in thermoelectrics by establishing a unified strategy for improving both performance and durability.

We have revised the Introduction to more clearly delineate the scope of our previous work from the present study, and have explicitly highlighted these novel aspects in the revised abstract (page 4):

"Our previous work demonstrated that introducing excess Y and minor Bi incorporation modifies charge carrier scattering at GBs and increases the Seebeck coefficient, leading to a peak *zT* of 1.23 at 970 K for $Y_{2.1}Te_3Bi_{0.13}$ with a two-fold increase in *zT* than $Y_{2.1}Te_3$[22]. However, the chemical stability and degradation behavior of these materials were not investigated in that study."

Comment 2:

"There are 9 figures presented in the manuscript. Although these systematic characterizations can explain well the degradation mechanisms, the discussions and explanations are too wordy. Therefore, I am not sure whether such a long and specialized presentation can draw most of the attention from the readers of Nature Communications."

Response 2:

We sincerely thank the Reviewer for this constructive comment. In the revised manuscript, we have therefore substantially streamlined the presentation while preserving the essential evidence supporting the degradation mechanism.

First, we reduced and reorganized the main figures. The original manuscript contained 9 main figures. In the revised version, we reduced the number of main figures to 7 and moved more specialized explanations to the Supplementary Information. The DFT results previously distributed across several figures have been consolidated into a single main figure, while detailed surface-defect energetics, chemical-potential-dependent defect stability, migration pathways, and electronic-structure analyses

have been moved to the Supplementary Information.

Second, we shortened the Introduction and Results sections to make the narrative more focused. The Introduction was revised to more directly frame the central knowledge gap. We also clarified the distinction between our previous work, which focused on thermoelectric transport, and the present study, which addresses chemical degradation and Bi-mediated stabilization.

Third, we condensed the experimental discussion in Sections 2.1 and 2.2. Repetitive descriptions of optical images, XRD peak evolution, SEM observations, FT-IR, and STEM-EDS maps were shortened or integrated into a more direct mechanistic sequence. Supporting details, such as secondary characterization results and extended methodological explanations, are now provided in the Supplementary Information where appropriate.

Fourth, we streamlined the APT and DFT discussions. In Section 2.3, we shortened the general description of the APT technique and focused on the key observations: GB-localized O and H enrichment, the reaction front in air-exposed $Y_{2.1}Te_3$, and the anticorrelation between Bi segregation and H plus O accumulation in Bi-$Y_{2.1}Te_3$. In Section 2.4, we condensed the DFT discussion by focusing on the main conclusions that Bi segregation to GBs is thermodynamically favorable, kinetically accessible, and modifies the local chemical environment of GBs toward H_2O-derived species. More specialized datasets are now placed in the Supplementary Information.

Overall, these revisions reduced the length of the manuscript by ~2000 words and reduced the number of main figures from 9 to 7. We believe that the revised manuscript now presents a clearer and more accessible storyline for a broad readership, while retaining the systematic evidence needed to support the proposed mechanism of moisture-induced degradation and Bi-mediated GB stabilization.

Comment 3:

"As shown in their Figure S5, the thermoelectric properties of their Y2.1Te3Bi0.13 samples are very poor. If the thermoelectric properties of their samples are very poor, I do not know whether the study of their chemical stability is meaningful."

Response 3:

We thank the Reviewer for this comment. We apologize for a unit error in the original Figure S5. The y-axis unit for electrical conductivity was incorrectly labeled as S m^{-1}. The correct unit is S cm^{-1}. This error has now been corrected in the revised Supplementary Information.

With the corrected unit, the electrical conductivity of $Y_{2.1}Te_3Bi_{0.13}$ is ~200 S cm^{-1} at room temperature and 120 S cm^{-1} at 970 K, consistent with our previous report on this composition (Rahman, Jamil Ur, et al. *Advanced Energy Materials* 15.14 (2025): 2404243). To further address the thermoelectric relevance of the material, we have added the thermal conductivity data and calculated the zT values in the revised manuscript and Supplementary Information (revised Figure 1 and Figure S5). The peak zT of $Y_{2.1}Te_3Bi_{0.13}$ reaches 1.23 at 970 K. This confirms that $Y_{2.1}Te_3Bi_{0.13}$ is a high-performance thermoelectric material and that studying its chemical stability is meaningful for thermoelectric applications.

Comment 4:

"I think they should present the aging-dependent thermoelectric characterization in their main text, when considering the main applications of Y2Te3 in thermoelectrics."

Response 4:

We agree with the Reviewer that aging-dependent thermoelectric characterization is essential to substantiate the practical relevance of our chemical stability findings. In the revised manuscript, we have added the aging-dependent thermoelectric dataset in the Figure 1 and Figure S5 as shown below. Notably, the aging-dependent measurements demonstrate that the chemical stability provided by Bi-segregated GBs translates directly into preserved thermoelectric performance. The peak zT of $Y_{2.1}Te_3Bi_{0.13}$ remains essentially unchanged after 48 h of ambient air exposure, with negligible variation in σ, S, and κ.

We also note that the thermoelectric properties of Bi-free $Y_{2.1}Te_3$ sample could not be measured after 1 h of air exposure because severe surface degradation rendered the sample electrically non-contactable, as mentioned in the manuscript. This observation further highlights the protective role of Bi incorporation.

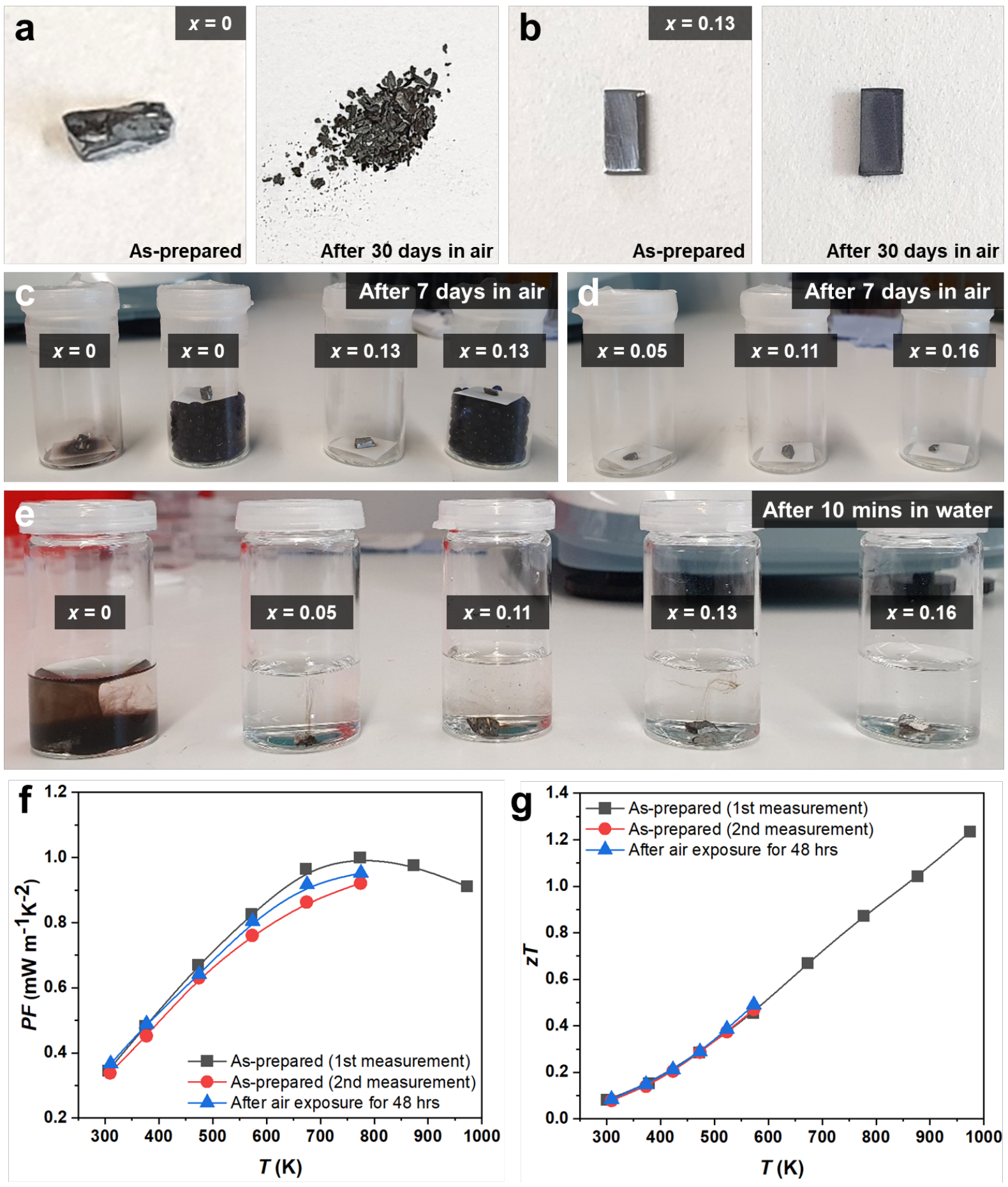

Fig. 1. Enhanced chemical stability of $Y_{2.1}Te_3Bi_x$ for air and water exposure by Bi incorporation. (f, g) power factor (PF) and *zT* of $Y_{2.1}Te_3Bi_{0.13}$ before and after air exposure for 48 hours.

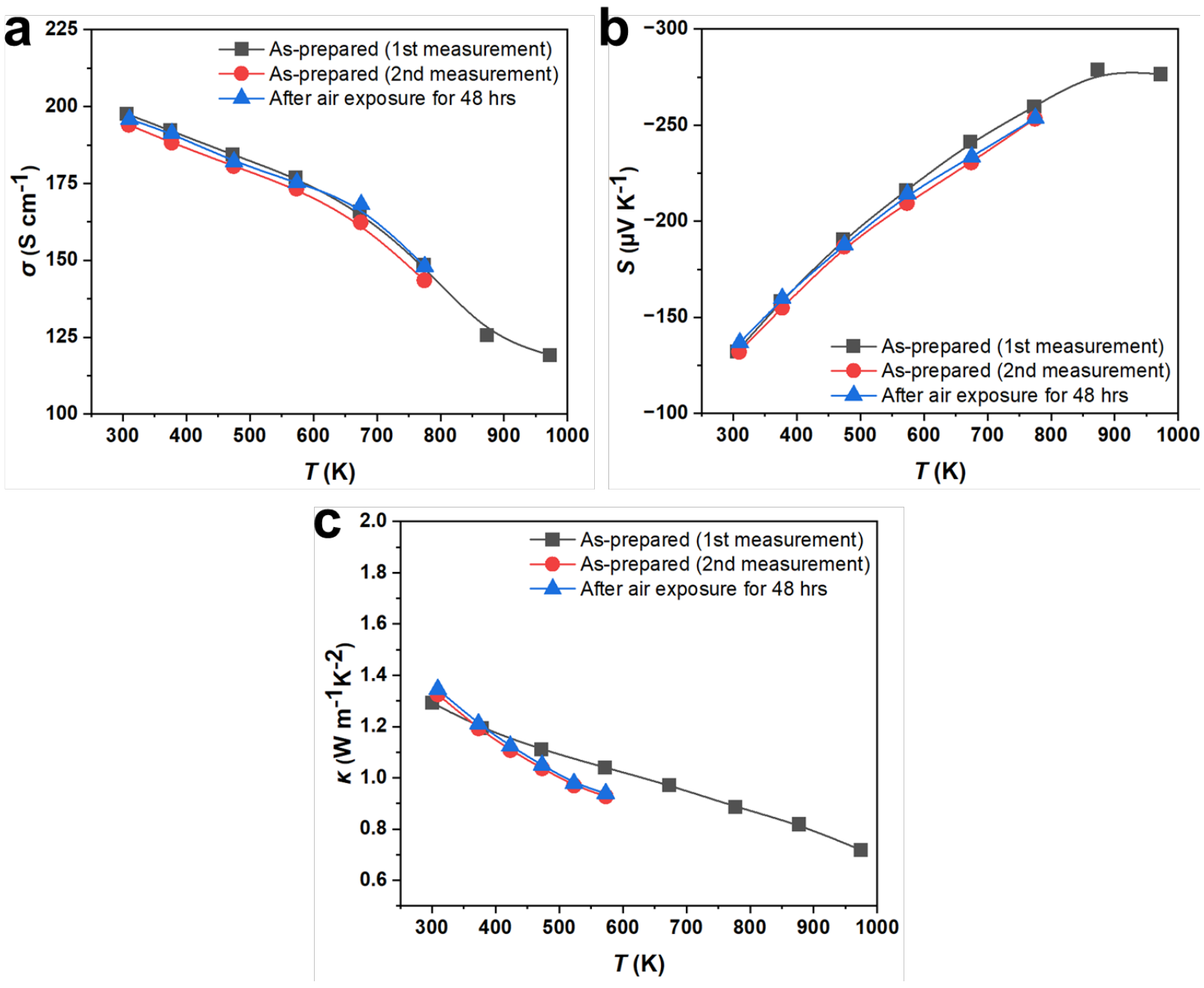

Figure S5. Thermoelectric transport properties of $Y_{2.1}Te_3Bi_{0.13}$ before and after 48 hours of air exposure. (a) Electrical conductivity, (b) Seebeck coefficient, and (c) thermal conductivity. The properties of the air-exposed sample remain unchanged within the measurement uncertainty.

Reviewer #2

The paper "Retarding moisture-induced chemical degradation of Yttrium Tellurides by tailoring grain boundary chemistry" presents an experimental study on the effect of Bi on the chemical degradation of $Y_{2.1}Te_3$. The main finding suggests that Bi segregation to grain boundaries plays a pivotal role in mitigating H_2O-induced chemical degradation. Below, I outline several comments and reservations regarding the interpretation of the results and the conclusions, which I would like to discuss with the authors.

Comment 1:

"The statement "Although some studies have begun to apply GB engineering to improve thermal stability[13,36], the potential of GB engineering in mitigating chemical degradation remains unexplored thus far" appears overly broad. While the effect of Bi grain-boundary segregation on the properties of Y_2Te_3 may indeed represent a novel contribution of the present manuscript, the general claim that "mitigating chemical degradation remains unexplored" is difficult to support. Several publications, including Zhou 2026 (DOI: 10.1039/d5ee06360d), Zheng 2021 (DOI: 10.1039/D1CS00347J), Abbey 2022 (DOI: 10.1039/D2EE02169B), and Wang 2021 (DOI: 10.30919/es8d513), together with the papers already cited in the manuscript, provide a foundation for understanding possible mitigation strategies based on grain boundary engineering. Therefore, I would recommend using a more precise and problem specific formulation rather than a general statement implying that this research direction has not been explored at all."

Response 1:

We sincerely thank the Reviewer for this constructive comment. We have accordingly revised the statement to a more precise and problem-specific wording and incorporated all the recommended references throughout the Introduction.

The revised passage in the Introduction now reads (page 4):

"More recently, it has also been applied to improve the thermal stability[26], and the broader role of GBs in governing both transport and stability of TE systems has been reviewed[27–30]. At the same time, GBs can act as fast diffusion pathways for reactive species, making them both functional interfaces and potential degradation pathways[31]. This duality of GBs makes GBs a compelling target for the rational design of multifunctional TE materials that combine high *zT* and long-term chemical stability. Despite

this progress, the potential of GB engineering for mitigating chemical degradation has received less attention to date."

Comment 2:

"Kinetic barriers for Bi…" a more usual term is "migration energy barrier"

Response 2:

We have replaced 'kinetic barrier' with 'migration energy barrier' in the revised manuscript.

Comment 3:

"How was the vacancy modelled in Y_2Te_3? Was it treated as a charged defect, and if so, how was the charge state selected? Moreover, how was this charge state incorporated into the DFT calculations? For the other defects considered in the study, were they treated as charged or neutral defects in the calculations?"

Response 3:

Thank you for these comments. Y_2Te_3 is a vacancy-ordered compound in which part of the cation sublattice is intrinsically vacant, and these ordered cation vacancies are already included in the crystallographic model used in our calculations. Therefore, we did not introduce additional vacancy defects beyond the intrinsic vacancy sites. Motivated by previous defect studies of bulk Y_2Te_3 (Toriyama, Michael Y., et al. *ACS applied materials & interfaces* 14.38 (2022): 43517-43526), we considered Bi interstitials and substitutional Bi defects at Y and Te sites as the relevant defect species in our surface and GB models.

In the present work, we employed charge-neutral supercells throughout the study and focused on comparing the relative energetic trends of Bi-related defects across bulk, surface, and GB environments, rather than explicitly evaluating multiple charged-defect states and charge-transition levels. Importantly, although neutral supercells were used, donor- or acceptor-like electronic behavior can still emerge naturally through local charge redistribution, as reflected in our PDOS and Bader charge analyses. While 3D charged-defect correction schemes such as the Freysoldt correction are widely used for bulk point defects, their application to extended interfacial systems such as surfaces and GBs is

nontrivial because of anisotropic dielectric screening and the absence of a unique bulk-like electrostatic reference. Therefore, we focused on structural relaxation and relative binding energy trends to provide a physically consistent comparison of Bi segregation behavior.

We have clarified the defect-modeling approach and the use of charge-neutral supercells in the revised Supplementary Information:

"Bi-related defects were modeled using charge-neutral supercells, which allows us to study general trends of defect-related energies, local charge redistribution and donor-/acceptor-like electronic behavior at GB."

Comment 4:

"Conclusions regarding Bi kinetics are based solely on migration barrier calculations. However, a diffusion coefficient generally contains not only the migration energy but also a prefactor, which can also be computed within DFT. Why was this prefactor neglected, and could the authors comment on how this omission affects the validity of conclusions drawn solely from migration-energy barriers?"

Response 4:

We agree that the diffusion coefficient formally includes both the migration barrier and the prefactor. In this work, our primary objective was to compare the relative diffusion kinetics between bulk and grain-boundary pathways, for which the migration barrier provides the dominant contribution. The prefactor was not explicitly evaluated due to the substantial computational cost associated with vibrational calculations, particularly for the large GB supercells considered here.

For the same diffusing species within the same host lattice, the prefactor is generally expected to remain largely unchanged within a relatively limited range, as it is primarily determined by vibrational frequencies and attempt rates within transition state theory (G. H. Vineyard, J. Phys. Chem. Solids 3, 121 (1957); Y. Mishin et al., Acta Materialia 58, 1117 (2010)). In contrast, the migration barrier is explicitly resolved and shows a lower value for GB diffusion (0.45 eV) compared to bulk diffusion (0.61 eV). This barrier difference (~0.16 eV) is expected to result in enhanced diffusivity along the GB over the temperature range relevant to synthesis and processing conditions. Therefore, while inclusion of prefactors would refine the quantitative diffusivity values, it is not expected to alter the qualitative conclusion that GB diffusion is kinetically favored over bulk diffusion. We have clarified this point in

the revised manuscript as below (page 26):

"Although diffusion prefactors were not explicitly evaluated, the migration barrier is expected to provide the dominant contribution when comparing relative diffusion kinetics for the same diffusing species within the same host lattice."

Comment 5:

"The authors consider only bulk Bi diffusion. Nevertheless, it is well known that, in polycrystalline materials, diffusion at relatively low temperatures is typically dominated by grain-boundary (GB) diffusion, whereas bulk diffusion becomes the dominant mechanism only at sufficiently high temperatures. Could the authors comment on the relevance and applicability of their conclusions, given that they are based exclusively on bulk-diffusion calculations?"

Response 5:

We thank the reviewer for this comment. We agree that GB diffusion is expected to dominate mass transport at relatively low temperatures in polycrystalline materials. In the original manuscript, bulk diffusion was considered to demonstrate that Bi is sufficiently mobile within the lattice to reach GBs, which is a prerequisite for segregation. To directly address the reviewer's point, we have now evaluated Bi migration along GBs. The calculated GB migration barrier for Bi_i diffusion (~0.45 eV) is lower than that in the bulk (0.61 eV), confirming that GB diffusion is kinetically favored. These results indicate that Bi atoms can first migrate through the bulk and subsequently diffuse more rapidly along GBs, thereby facilitating Bi segregation at GBs. Therefore, our conclusions remain valid under realistic polycrystalline conditions, where bulk diffusion governs transport to GBs while GB diffusion dominates along the boundary. We have clarified this point in the revised manuscript.

Comment 6:

"Please provide more complete computational details for the CI-NEB calculations. Additional information is needed to ensure reproducibility. Specifically, please include the number of images used and provide an illustration of the selected atomic migration pathways (in at least one additional projection). Such visualization also requires justification, as multiple diffusion pathways or mechanisms may be possible. Why was only a single atomic migration mechanism considered?"

Response 6:

To improve reproducibility and clarify the migration mechanism, we have added additional computational details for the CI-NEB calculations in the revised Methods section. We have also revised the diffusion figure to more clearly visualize the selected migration pathways in both the bulk and GB environments. In bulk Y_2Te_3, diffusion occurs through geometrically constrained ordered-vacancy channels, and the representative nearest-neighbor hopping pathways are therefore most effectively visualized within the vacancy network. In contrast, the GB diffusion process is highly localized within the structurally disordered interface region. Multiple simultaneous projections tend to obscure the local diffusion geometry in the disordered GB environment. Accordingly, for the GB pathway, we visualized the local hopping event using the initial, transition-state, and final configurations.

The diffusion pathway was constructed using a nearest-neighbor hopping mechanism based on the local atomic geometry. In the vacancy-ordered Y_2Te_3 structure, the available diffusion channels form a narrow and well-defined low-energy diffusion network. Alternative migration pathways are either symmetry-equivalent or involve significantly longer hops through sterically hindered regions and are therefore not expected to contribute substantially to diffusion. We have clarified these points in the revised Supporting Information:

"To determine the diffusion pathway, a nearest-neighbor hopping mechanism was constructed based on the local atomic geometry. In the Y_2Te_3 structure, the diffusion channels are geometrically constrained and form a narrow and well-defined low-energy diffusion network due to the connectivity of available vacancy sites. Alternative migration pathways are either symmetry-equivalent or involve significantly longer hops through sterically hindered regions, and are therefore not expected to contribute substantially to diffusion."

Comment 7:

"The statement "To the best of our knowledge, this work represents the first demonstration of enhanced chemical durability in thermoelectric materials through GB segregation" is not fully convincing. Based on both the DFT and experimental results presented, the evidence does not yet appear sufficient to attribute the mitigation of material degradation primarily to Bi grain boundary (GB) segregation.

Is there any evidence that H_2O diffusion occurs primarily along GBs? The proposed mechanism involves GB or bulk mediated diffusion processes associated with the dissociation of H_2O into H^+ and OH^- at the surface (a step not shown in Fig. 6), followed by atomic—rather than

molecular—diffusion. Do the authors have any evidence for molecular H_2O diffusion to support their conclusion, or is the mechanism assumed? Clarification on the dominant diffusing species (molecular vs. dissociated species) is necessary for interpreting the results."

Response 7:

We thank the reviewer for this comment. We agree that clarifying the dominant diffusing species is important for interpreting the degradation mechanism and the role of GBs. To address this point, we have performed additional adsorption calculations for representative H_2O-related species, including H_2O, O, and OH, at both clean and Bi-containing GBs.

Unique adsorption sites were first identified using graph-based adsorption site analysis, followed by pre-relaxation using the universal ML potential (MACE-MH-1). The five most stable candidate structures for each species were subsequently refined using DFT calculations. The binding energy at the GB was defined as:

$$E_{\mathrm{bind}}(X) = E_{\mathrm{GB}+X} - E_{\mathrm{GB}} - E_X,$$

where *X* denotes H_2O, O, or OH. For atomic O adsorption, a $\frac{1}{2}\mathrm{O}_2$ reference was used.

The calculated binding energies clearly distinguish the behavior of molecular and dissociated species. Molecular H_2O exhibits relatively moderate binding energies, ranging from –1.02 to –1.13 eV (average –1.11 eV) at the clean GB and from –1.09 to –1.29 eV (average –1.17 eV) at the Bi-containing GB, indicating only a minor change upon Bi incorporation. In contrast, dissociated species bind substantially more strongly. O adsorption energies ranging from –2.19 to –3.67 eV (average –2.82 eV) at the clean GB and from –2.26 to –3.70 eV (average –3.07 eV) at the Bi-containing GB. An even more pronounced effect is observed for OH, whose binding energies increase from –4.55 to –5.09 eV (average –4.98 eV) at the clean GB to –5.62 to –5.74 eV (average –5.69 eV) at the Bi-containing GB.

Importantly, the strengthening effect induced by Bi is significantly larger for dissociated species, particularly OH (~0.7 eV), than for molecular H_2O (<0.1 eV). This indicates that Bi selectively enhances the stabilization of dissociated species at the GB, rather than molecular H_2O itself. Furthermore, OH adsorption remains centered on neighboring Y atoms instead of directly binding to Bi sites, consistent with the experimentally observed Y-O-H phases. Interestingly, the most stable OH configuration changes from a three-fold coordinated motif at the clean GB to a two-fold coordinated motif at the Bi-containing GB despite the stronger binding energy, suggesting that the enhanced stabilization originates primarily from the electronically modified GB environment by Bi incorporation

rather than direct Bi-OH bonding. These results are also consistent with our APT observations, which reveal clear segregation of O and H at GBs. This suggests that dissociated O- and H-containing species are likely to be more relevant to the GB degradation chemistry rather than molecular H_2O. In addition, OH preferentially binds to neighboring Y sites rather than directly to Bi, forming Y-OH-like motifs that are consistent with the experimentally observed Y-O-H phases. We have clarified this point in the revised manuscript with newly added Figure 7.

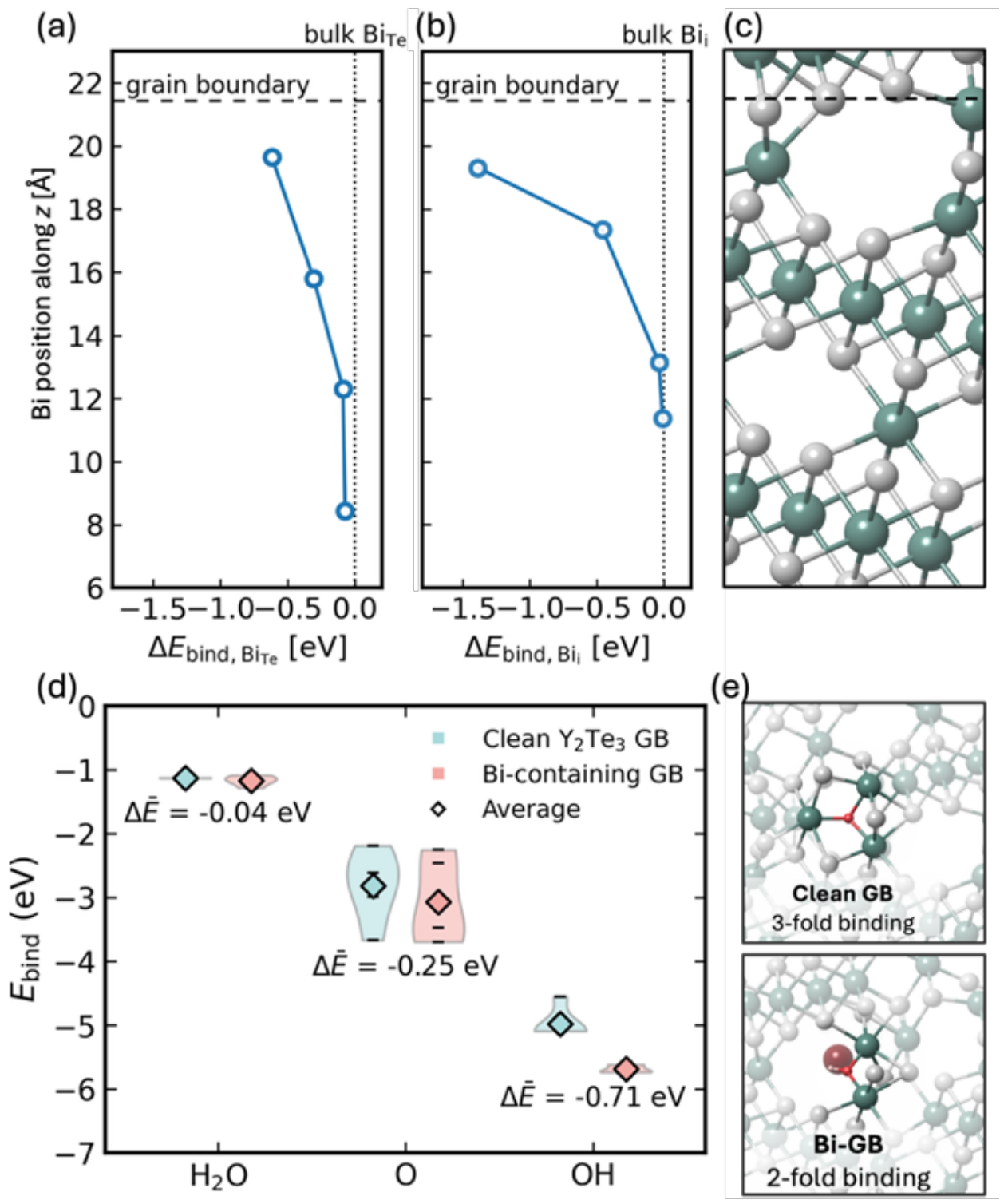

Fig. 7. Binding energy behavior of Bi defect and H_2O-related species at GBs. (a-c) Relative binding energy of Bi_{Te} (a) and Bi_i (b) as a function of distance from the GB plane with (c) the atomic structure of the Σ3 GB (cyan: Y, white: Te). The zero reference corresponds to the bulk defect energy, and horizontal dashed lines indicate the GB core. (d) Binding energy distributions of H_2O, O, and OH adsorbed at clean (blue) and Bi-containing (red) GBs. (e) Representative adsorption configurations of OH at clean and Bi-containing GBs.

Comment 8:

"What is the possible role of Bi surface segregation? Why is surface segregation of Bi excluded from the conclusions? Bi enrichment at surfaces may influence oxidation resistance, adsorption energetics, or surface reaction pathways. This should be discussed explicitly."

Response 8:

We thank the Reviewer for raising this important point. We agree that Bi surface segregation should be discussed explicitly. In the revised manuscript, we now further clarify that Bi enrichment may contribute to delaying the initial surface reaction.

Indeed, our data provide evidence for Bi enrichment at surfaces or near-surface regions, including the metallic Bi peak in the XRD pattern of as-prepared Bi-$Y_{2.1}Te_3$, discrete Bi particles observed by SEM/STEM, and DFT calculations showing favorable stabilization of Bi-related defects at the Y_2Te_3 surface. The smaller Te nodules observed on air-exposed Bi-$Y_{2.1}Te_3$ compared with Bi-free $Y_{2.1}Te_3$ are also consistent with a reduced initial surface reaction rate.

However, we do not attribute the surface segregation as a dominant stabilization mechanism. The degradation of Bi-$Y_{2.1}Te_3$ is not completely stopped at the surface, as a degraded near-surface region of approximately 6 μm is still observed after 30 days of air exposure. This shows that moisture can penetrate the surface even in the presence of Bi enrichment. In contrast, further degradation into the bulk is strongly retarded, indicating that the key difference lies in the propagation step through the GB network.

We therefore interpret Bi surface segregation as a contributing factor that can delay the onset of surface oxidation, whereas Bi segregation at GBs controls the subsequent transport and propagation of degradation. This assignment is supported by the APT results, which show a direct local anticorrelation between Bi interfacial excess and H plus O accumulation at individual GBs. Thus, the GB-level correlation provides direct evidence that Bi-segregated GBs suppress H_2O ingress and govern the dominant protection mechanism.

To make this point clear, we have added the following discussion to the revised manuscript (page 28):

"Bi enrichment at surfaces may also contribute to the improved resistance against the initial surface reaction, as suggested by the smaller Te nodules formed on Bi-Y_2Te_3 after air exposure. However, the presence of a degraded near-surface region after prolonged exposure shows that surface

enrichment alone does not fully block moisture ingress. Instead, APT reveals a direct anticorrelation between Bi segregation and H plus O accumulation at individual GBs, indicating that Bi-segregated GBs control the subsequent propagation of degradation into the bulk. We therefore consider Bi surface segregation as a secondary contribution that delays the onset of degradation, while GB segregation provides the dominant mechanism for suppressing moisture transport into the bulk and the resulting chemical degradation."

Comment 9:

"Bi is generally known to reduce GB cohesion, as documented in several studies. Therefore, proposing that Bi improves resistance to chemical degradation via GB segregation appears counterintuitive.

o Did the authors evaluate the effect of Bi on the GB work of separation in their DFT calculations?

o How does Bi segregation modify GB decohesion resistance?

o How does this behavior change in the presence of H and O species?

Such DFT based insights—if available—would provide a stronger physical basis for the proposed hypothesis. Alternatively, a micro pillar bending test on GBs with and without Bi segregation, before and after oxidation, could help validate the mechanical implications of Bi segregation experimentally."

Response 9:

Thanks for the reviewer's comments. We evaluated the work of separation (W_{sep}) for both clean and Bi-containing GBs by considering multiple GB-parallel cleavage planes. The minimum values are 0.043 eV/Å^2 (clean) and 0.042 eV/Å^2 (Bi-containing), indicating that Bi does not significantly change the intrinsic GB decohesion resistance within DFT accuracy. This suggests that the commonly reported embrittling effect of Bi is not strongly manifested in the clean GB structure considered here. For reference, the obtained values are higher than typical interlayer separation energies of van der Waals systems such as graphite (~0.02 eV/Å^2), indicating that the GB is not weakly bound in a van der Waals-like manner. Therefore, the experimentally observed long-term GB degradation is unlikely to originate from immediate mechanical weakening. In the presence of H and O species, our adsorption calculations show that Bi segregation preferentially stabilizes H_2O/OH/O-related species at GB. Therefore, the role of Bi in this system is better understood as modifying the chemical degradation pathway rather than directly reducing GB cohesion. We have clarified this point in the revised manuscript (page 26):

“This trapping mechanism is further supported by our calculation for work of separation of GB (See Supplementary text S4), which shows that the presence of Bi does not compromise intrinsic GB cohesion, allowing Bi to remain stably incorporated without inducing mechanical instability.”

and Supporting Information:

S4. Effect of Bi segregation on GB cohesion

To investigate whether Bi segregation induces mechanical embrittlement, we evaluated the work of separation (W_{sep}) for both clean and Bi-containing GBs. We considered multiple cleavage planes parallel to the GB interface to identify the minimum energy.

The calculated minimum W_{sep} values are 0.043 eV/Å^2 for the clean GB and 0.042 eV/Å^2 for the Bi-containing GB. This negligible difference indicates that Bi incorporation does not significantly alter the intrinsic decohesion resistance of the GB within DFT accuracy. These values suggests that the commonly reported embrittling effect of Bi is not strongly manifested in the clean GB structure considered in this work.

Reviewer #3

The manuscript titled "Retarding moisture-induced chemical degradation of Yttrium Tellurides by tailoring grain boundary chemistry" has been reviewed. This study systematically investigates the reason behind an enhanced mechanical and chemical stability of Y2Te3 under ambient conditions as an emerging n-type thermoelectrical material. While grain boundary (GB) engineering is used to improve thermoelectric performance, it's role in mitigating chemical degradation remains a largely unexplored area.

Using advanced characterization techniques, the authors reveal that moisture-driven degradation occurs via rapid H2O diffusion along GBs, leading to oxidation into Y-O-H phase, Te segregation into a relatively pure form, and eventual chemo-mechanical failure with increased exposure time. Remarkably, incorporating about 1at% Bi significantly retards this process by segregating at GVs, forming a chemically passivated layer that blocks water ingress. APT is used to map the compositional distribution show a reduced O and H enrichment at Ri-rich GVs, while DFT calculations indicate that Bi segregation is thermodynamically favorable. The addition of Bi not only improves chemical durability but also enhances the thermoelectric properties. These finding establish GB segregation as a multifunctional engineering strategy for simultaneously optimizing structural, chemical, and performance stability.

The manuscript is very well written with an appropriate presentation of background and motivation followed by a logical presentation of results. I find no fatal issues with the results and the interpretation thereof. However, before the manuscript should be considered for publication here or elsewhere, the authors should address the following questions, comments, or recommendations, which are presented in chronological order.

Comment 1:

"Page 7: Regarding the description of the use of silica gel in the text and the images in Figure 1, I assume that the silica gel is the thick black layer in the right-hand-side vial for x=0 and x=0.13. Please clarify this since silica gel is usually somewhat clear in appearance with a yellowish tint."

Response 1:

We thank the Reviewer for pointing out this potential source of confusion. We confirm that the dark material at the bottom of the vials in Figure 1c (right-hand side, for x = 0 and x = 0.13) is indeed

silica gel. The dark appearance arises from the specific type of silica gel used in our laboratory, which differs in color from the more familiar transparent or yellowish variants. To avoid confusion, we have revised the caption of Fig. 1 to explicitly state that the dark material at the bottom of the corresponding vials is silica gel. The revised caption now reads (page 7):

"For x = 0 and 0.13, additional samples were stored in vials containing silica gel (dark material at the bottom of the right-hand-side vials) to distinguish the effects of dry and humid air. "

Comment 2:

"Page 9: When describing the results of Figure 2 in the text, please include mention about the lack of not seeing any Y-oxide in the XRD, stating that this is likely due to it being an amorphous phase and refer to results later in the manuscript that corroborate this. As a reader, I was left wondering about this in the back of my mind until it was addressed later (page 11)."

Response 2:

We thank the Reviewer for this helpful suggestion. We agree that introducing the absence of crystalline Y-oxide phases in the XRD patterns at the point of first mention will improve the readability.

In the revised manuscript, we have added the following sentence at the end of the XRD discussion in Section 2.2 (page 10):

"Notably, no crystalline Y-oxide phases were detected in the XRD patterns, likely due to their amorphous or nanocrystalline nature as further confirmed by Fourier-transform infrared (FT-IR) spectroscopy and high-resolution STEM discussed below"

Comment 3:

"Page 13: Regarding "results indicate that the overall Y content of the matrix is preserved during degradation, thus…" – this is not clear from the EDS images. Provide an EDS line scan to better support this claim."

Response 3:

We agree with the Reviewer that an EDS line scan provides more direct evidence than elemental maps alone. We have added an EDS line scan across the degradation front of the air-exposed Bi-$Y_{2.1}Te_3$ specimen. The new line scan, presented as Figure S14 in the revised Supplementary Information, shows that the Y signal remains nearly constant across the interface, whereas the Te concentration decreases from approximately 49 at.% in the pristine region to approximately 32 at.% in the degraded region, and the O concentration correspondingly increases from approximately 26 at.% to approximately 40 at.%. These quantitative profiles confirm that the degradation front is governed by Y-O-H formation and Te redistribution, while the overall Y content of the matrix remains essentially conserved.

The corresponding statement in the main text has been revised to explicitly cite this new figure (page 12):

"Across this front, EDS maps showed Te depletion from the degraded surface (31.6±6.7 at.%) than the $Y_{2.1}Te_3$ bulk below (49.2±7.8 at.%) and O enrichment (40.0±6.7at.% (top); 25.7±3.0 at.% (bottom)), whereas Y concentration remained nearly constant (28.4±6.3 at.% (top); 25.2±5.9 at.% (bottom)) (Fig. 2k). A complementary EDS line scan across the degradation front directly visualizes these compositional changes (Fig. S14)."

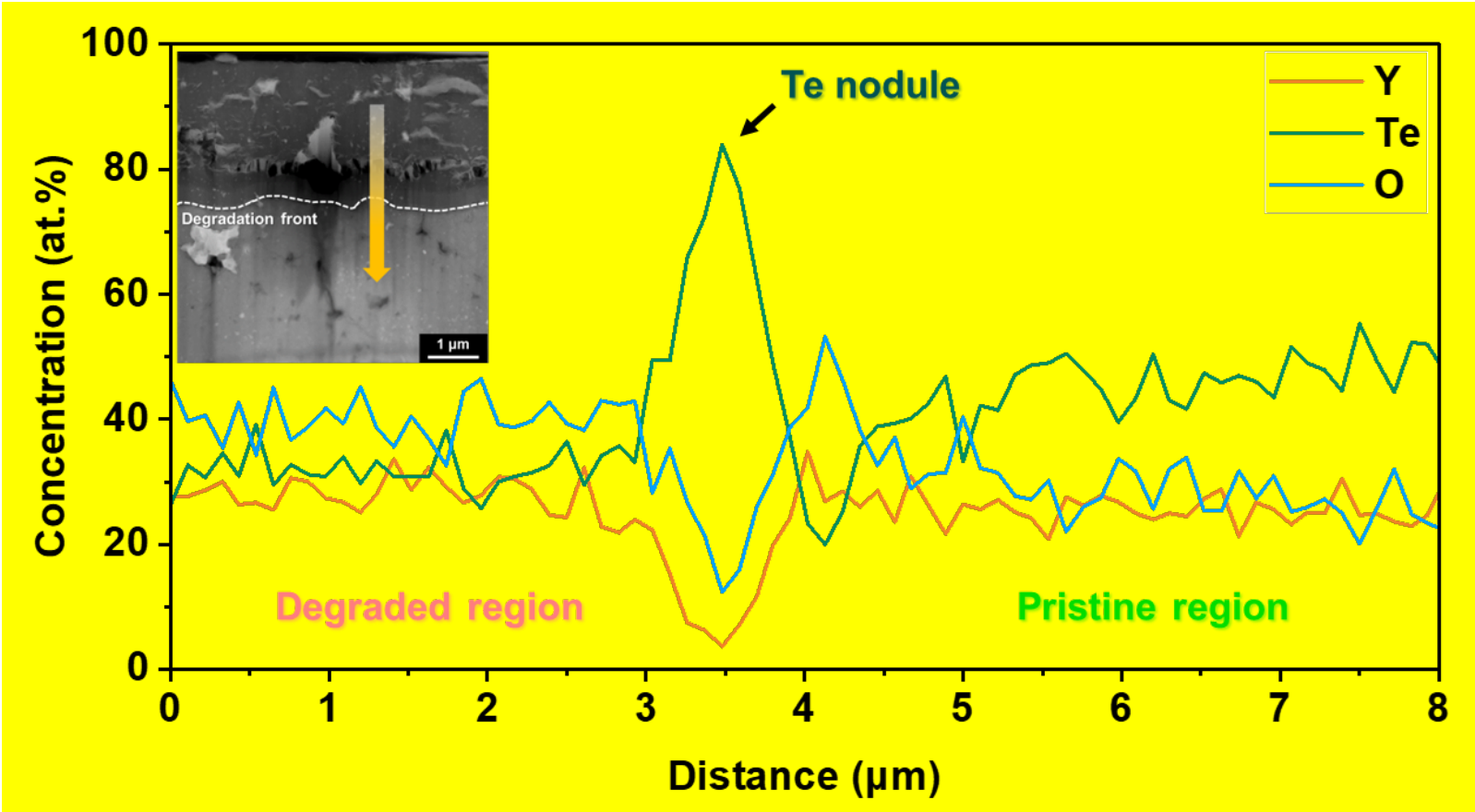

Figure S14. EDS line scan across the degradation front of the air-exposed Bi-$Y_{2.1}Te_3$ sample. The inset shows a cross-sectional HAADF-STEM image where the degradation front is indicated by the white dashed line. The yellow arrow marks the position and direction of the line scan, which traverses from the degraded surface region (left) through the degradation front into the pristine region (right). The Bi signal is not shown because of its low concentration and limited counting statistics.

Comment 4:

"Page 16: what is "ion projection microscopy". Think you mean to say Field ion microscopy which is a more common/proper description keeping with the jargon of the field."

Response 4:

We thank the Reviewer for this terminological correction. We have replaced "ion projection microscopy" with "field ion microscopy" in the revised manuscript to align with the standard nomenclature used in the atom probe tomography community (page 15):

"The compositional evolution within the bulk and along GBs during the degradation was further investigated using APT, which is an advanced analytical technique that combines field ion microscopy with time-of-flight mass spectrometry"

Comment 5:

"Page 16: Regarding "Because of their extremely small volumes and large surface-to-volume ratios, the air-exposed specimens of both Y2.1Te3 and Bi-Y2.1Te3 rapidly degraded and showed noticeable surface transformations during exposure", please show SEM images of this "noticeable surface transformation" in the supplementary information section."

Response 5:

We thank the Reviewer for this suggestion. SEM images of the APT specimens before and after 7 days of air exposure are provided in Figure S17 of the original Supplementary Information, which clearly shows the surface transformation of the tips. To make this more accessible to readers, we have updated the corresponding sentence in the main to explicitly cite this figure text (page 15):

"The air-exposed specimens showed noticeable surface transformations during exposure due to the large surface-to-volume ratios of APT specimens (Fig. S17)."

Comment 6:

"Please include a clearly labeled APT mass spectrum in the supplementary information section, for both samples. Also include a table of both the ionic and decomposed atomic compositions in the supplementary information section."

Response 6:

We appreciate the Reviewer for this constructive suggestion. In the revised Supplementary Information, we have added clearly labeled APT mass spectra for both $Y_{2.1}Te_3$ and Bi-$Y_{2.1}Te_3$ specimens, including the as-prepared and air-exposed states as new Figure S28 and Figure S29. We have also added new tables, Table S2 and Table S3, summarizing both the ionic compositions and the decomposed atomic compositions for each analyzed specimen.

The mass spectra also contain minor peaks assigned to Ca, Ti, and Dy. These trace impurities are likely attributed to the Y raw material (which has a nominal purity of 99.9%), consistent with previous reports (Mukherjee, Abhishek, A. Awasthi, and N. Krishnamurthy. *Mineral Processing and Extractive Metallurgy* 125.1 (2016): 26-31; Cheremisina, Olga, et al. *Metals* 9.12 (2019): 1317), Importantly, these minor species are present in both $Y_{2.1}Te_3$ and Bi-$Y_{2.1}Te_3$ specimens at very low

concentrations (typically ≤0.1 at.%) and do not show spatial correlation with grain boundaries or degraded regions. Therefore, they do not affect the interpretation of the APT results or the conclusion that Bi segregation at GBs suppresses moisture ingress and degradation.

We have added the following sentence in the Methods section (page 32):

"The APT mass spectra (Figs. S28 and S29), ionic compositions (Table S2) and atomic compositions (Table S3) of each specimen are provided in Supplementary Information.

"

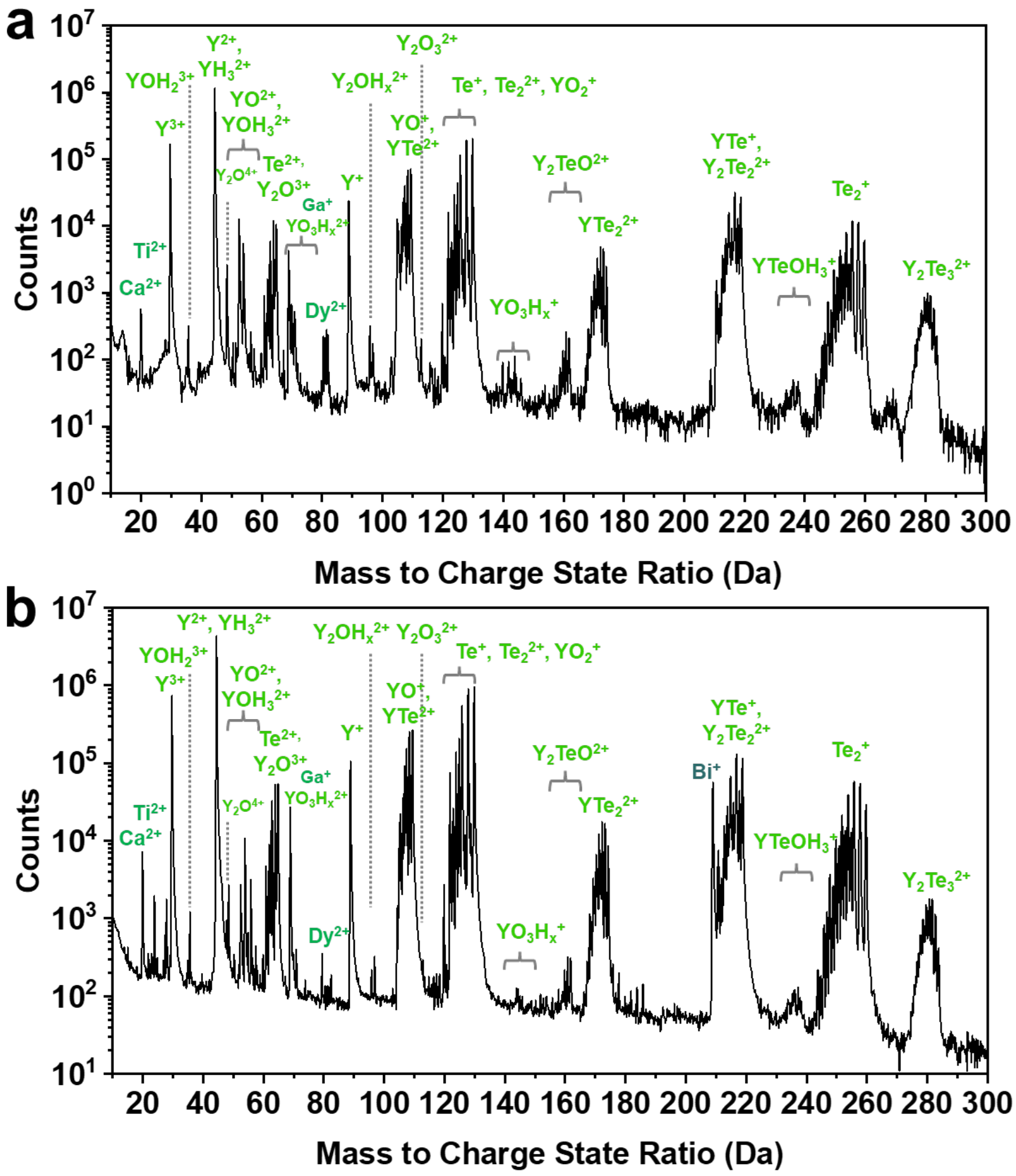

Figure S28. APT mass spectra of the as-prepared (a) $Y_{2.1}Te_3$ and (b) Bi-$Y_{2.1}Te_3$. Major matrix and dopant-related peaks appeared, whereas minor trace impurity or preparation-related peaks, including Ca, Ti, Dy, and Ga also exist. Ca and Dy are likely associated with trace impurities in the Y raw material (Cheremisina, Olga, et al. *Metals* 9.12 (2019): 1317), while Ga is attributed to FIB preparation.

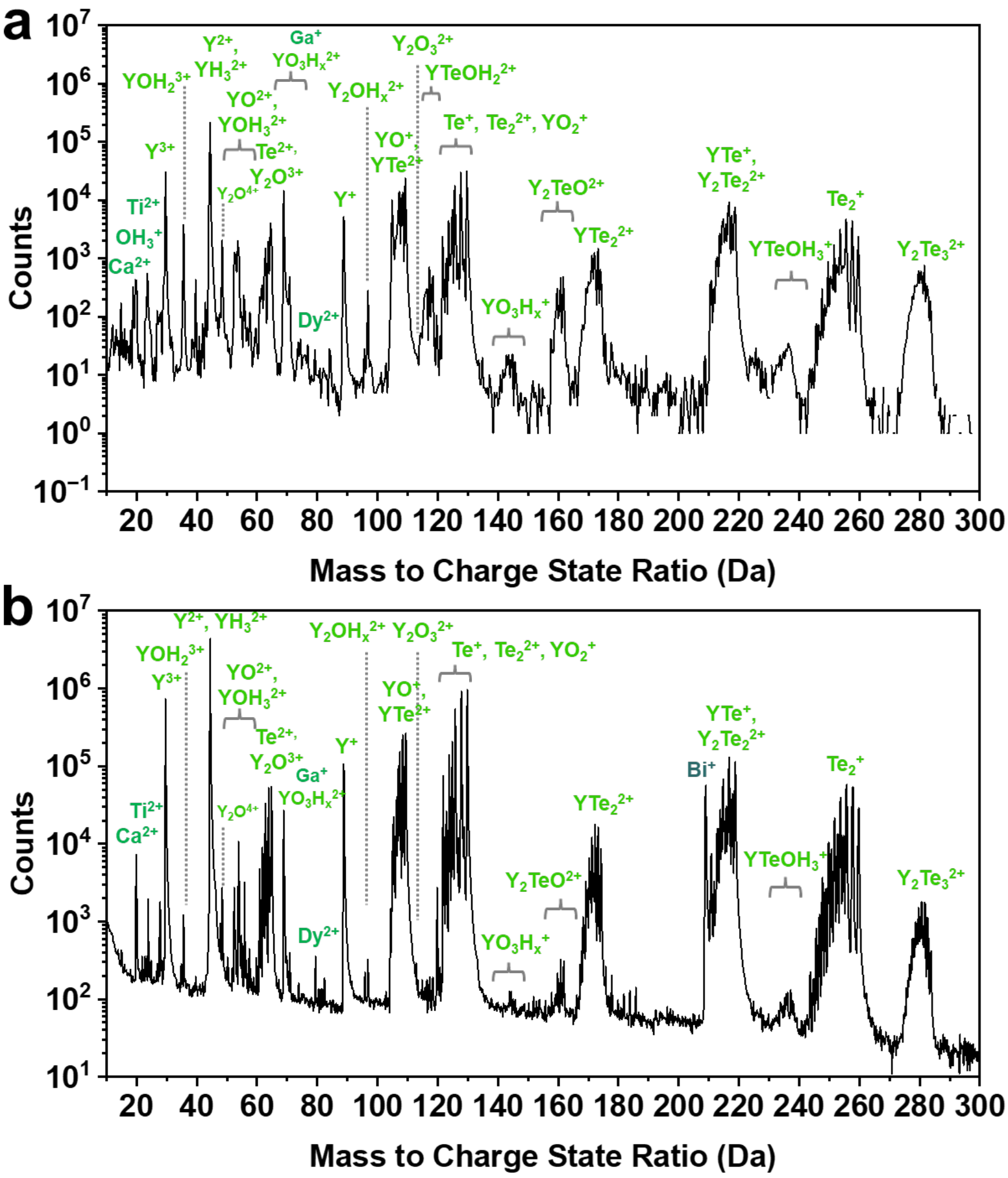

Figure S29. APT mass spectra of the air-exposed (a) $Y_{2.1}Te_3$ and (b) Bi-$Y_{2.1}Te_3$ for 7 days.

Table S2. Ionic compositions from APT mass spectra.

Ion species	As-prepared $Y_{2.1}Te_3$ (ion.%)	As-prepared Bi-$Y_{2.1}Te_3$ (ion.%)	Air-exposed $Y_{2.1}Te_3$ (ion.%)	Air-exposed Bi-$Y_{2.1}Te_3$ (ion.%)
**Y**	27.28	32.24	19.53	2.91
**Te**	36.10	36.73	27.34	22.54
**Bi**	0.00	1.02	0.00	0.55
**YTe**	23.42	20.00	29.69	50.59
**YTe_2**	1.54	0.000	1.89	4.57
**TeY_2**	0.00	0.000	0.00	0.03
**Y_2Te_2**	1.73	1.33	3.78	14.83

**Te_2Y**	0.00	1.21	0.00	0.00
**Te_2**	6.68	6.36	7.03	0.59
**Y_2Te_3**	0.66	0.26	1.44	0.44
**YO**	0.99	0.10	2.79	0.84
**Y_2O**	0.26	0.08	1.51	0.08
**Y_2O_3**	0.01	0.002	0.00	0.00
**YO_2**	0.004	0.002	0.00	0.00
**Y_2TeO**	0.07	0.02	0.46	0.00
**TeY_2O**	0.00	0.00	0.00	1.25
**Y_3Te_2O**	0.00	0.00	0.00	0.04
**Te_2Y_2O**	0.00	0.00	0.00	0.02
**YOH**	0.00	0.01	0.25	0.003
**YOH_2**	0.01	0.01	1.02	0.01
**YOH_3**	0.32	0.11	0.71	0.15
**YH_3**	0.07	0.02	0.03	0.03
**$YTeOH_2$**	0.00	0.00	0.63	0.00
**$YTeOH_3$**	0.00	0.00	0.05	0.09
**YTe_2H_3**	0.00	0.00	0.00	0.02
**YO_3H_3**	0.04	0.003	0.08	0.00
**YO_3H_5**	0.04	0.004	0.12	0.01
**OH**	0.00	0.00	0.01	0.00
**OH_2**	0.00	0.00	0.01	0.001
**OH_3**	0.00	0.00	0.08	0.00
**O**	0.00	0.002	0.01	0.00
**Ca**	0.04	0.10	0.18	0.12
**Ti**	0.05	0.06	0.17	0.25
**Dy**	0.06	0.00	0.02	0.02
**Ga**	0.63	0.31	1.20	0.04

Table S3. Decomposed atomic compositions from APT mass spectra.

**Element**	**As-prepared $Y_{2.1}Te_3$ (at.%)**	**As-prepared Bi-$Y_{2.1}Te_3$ (at.%)**	**Air-exposed $Y_{2.1}Te_3$ (at.%)**	**Air-exposed Bi-$Y_{2.1}Te_3$ (at.%)**
**Y**	40.85	42.37	40.45	43.49
**Te**	56.25	55.90	50.01	54.45
**Bi**	0.000	0.76	0.00	0.26
**O**	1.33	0.27	4.60	1.17
**H**	1.03	0.35	4.00	0.43
**Ca**	0.03	0.07	0.10	0.06
**Ti**	0.03	0.05	0.09	0.12
**Dy**	0.04	0.00	0.01	0.01
**Ga**	0.43	0.23	0.68	0.02

Comment 7:

"Since the APT samples were Ga FIB-prepared, please comment on the surface damage as indicated by implanted Ga. Is there a spatial correlation with the Ga distribution and "degraded" regions in the sample. What I'm getting at is if/how Ga (if present) may be influencing your results. Surface damage from Ga could lead to observing more water and hydrocarbons on the damaged surface from air exposure."

Response 7:

We appreciate the Reviewer raising this important point. We agree that Ga implantation and FIB-induced surface damage must be carefully considered when interpreting O and H signals in APT datasets. We therefore evaluated whether Ga is spatially correlated with the degraded regions observed in the air-exposed specimens.

All APT specimens were prepared with a final low-kV Ga milling step to minimize FIB-induced damage. Nevertheless, residual Ga was still detected in some datasets, with the amount varying from specimen to specimen. To address this explicitly, we have added the spatial distributions of Ga ions in the APT reconstructions of the air-exposed $Y_{2.1}Te_3$ and Bi-$Y_{2.1}Te_3$ specimens as Figure S20 in the revised Supplementary Information.

This analysis shows no detectable spatial correlation between Ga and the O-rich degraded regions. In the air-exposed $Y_{2.1}Te_3$ specimen, Ga was detected at 0.68 at.% and distributed rather homogeneously throughout the analyzed volume, without enrichment at the O-rich degraded region, grain boundaries (GBs), or degradation front. In the air-exposed Bi-$Y_{2.1}Te_3$ specimen, the Ga concentration was much lower, approximately 0.02 at.%, and the weak Ga signal was mainly observed near the top or relatively pristine region rather than in the degraded region. This distribution is consistent with minor residual Ga from the final FIB preparation or re-sharpening step and does not follow the spatial distribution of O and H enrichment.

Therefore, the observed degradation features cannot be explained by Ga-induced damage. If Ga implantation were responsible for the increased O and H signals, one would expect Ga to be spatially correlated with the O-rich degraded regions or to show enrichment at the same GBs. Instead, O and H enrichment is localized at GBs and degradation fronts, while Ga shows no such correlation. Moreover, in Bi-$Y_{2.1}Te_3$, the key observation is the anticorrelation between Bi interfacial excess and H plus O accumulation at individual GBs, which cannot be explained by Ga implantation.

To make this point explicit, we have added the following sentence to Section 2.3 of the revised manuscript (page 20):

"Possible effect of Ga implantation on such O and H enrichment was evaluated by analyzing the Ga distribution in the air-exposed APT specimens. Although residual Ga was detected in the datasets, Ga showed no spatial correlation with O-rich degraded regions, GBs, or degraded regions in both $Y_{2.1}Te_3$ and Bi-$Y_{2.1}Te_3$ (Fig. S20), indicating that the observed O and H enrichment is not governed by Ga-induced damage."

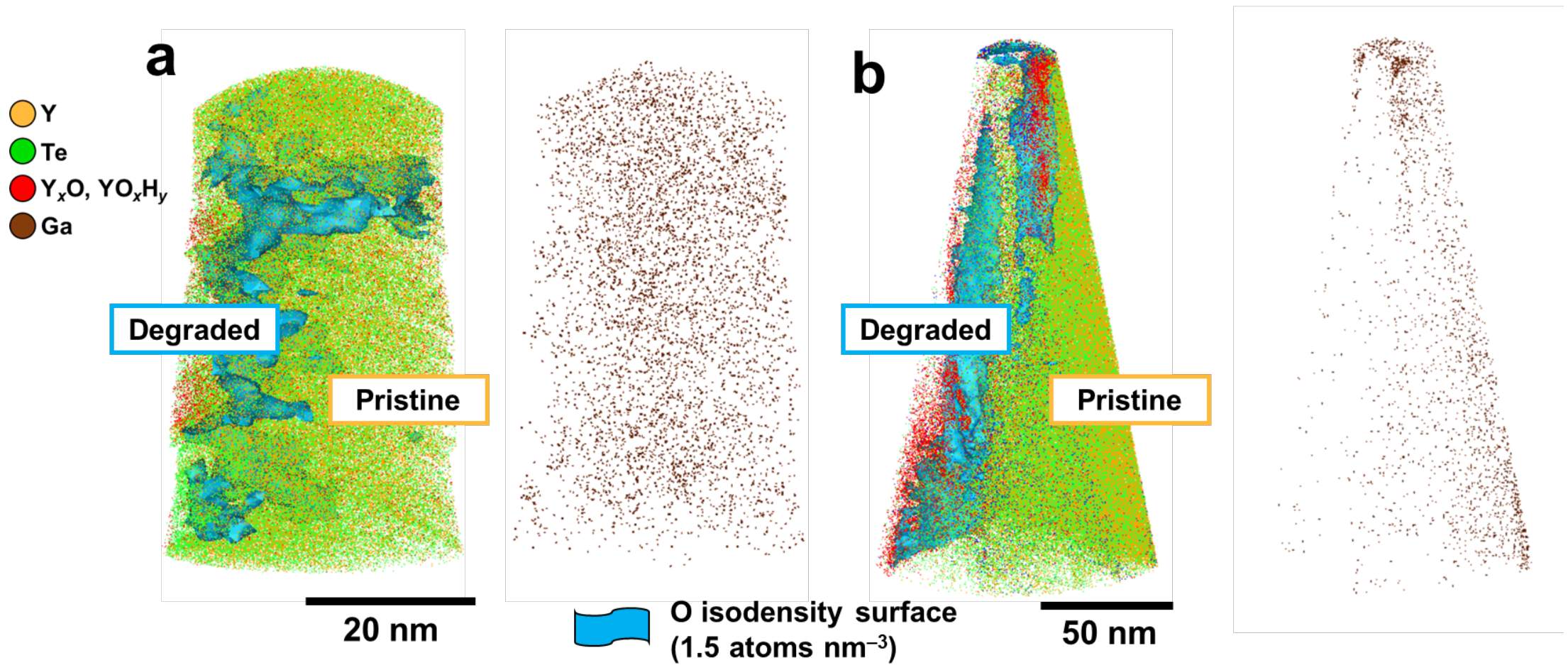

Figure S20. Spatial distribution of Ga in air-exposed APT datasets. (a, b) APT reconstructions of air-exposed (a) $Y_{2.1}Te_3$ and (b) Bi-$Y_{2.1}Te_3$, each shown with (left) the full atomic distribution including the O isodensity surface (1.5 atoms nm^{-3}) showing the degraded and pristine regions, and (right) the corresponding Ga-only distribution. In both specimens, Ga atoms are dispersed throughout the analyzed volume without preferential accumulation at grain boundaries or in the degraded regions, indicating no spatial correlation between Ga implantation from FIB specimen preparation and the moisture-induced degradation features.

Comment 8:

"I find it surprising that you got such large amounts of data from the samples without Bi that were exposed to air. If chemo-mechanical degradation is occurring, I would expect the samples to be relatively brittle leading to unstable field evaporation and premature fracture before significant data can be collected. Please provide a comment."

Response 8:

We thank the Reviewer for this thoughtful question. We agree that fully chemo-mechanically degraded $Y_{2.1}Te_3$ is expected to be mechanically fragile and prone to premature fracture during field evaporation. Indeed, a fraction of our air-exposed specimens did fracture prematurely, and the overall yield of successful APT measurements was much lower for these tips than for the as-prepared ones. However, representative datasets could still be obtained because the analyzed volumes did not correspond to the fully disintegrated outer regions.

We would like to clarify the specimen preparation workflow: FIB-cut lamellae mounted on Si posts were exposed to ambient air for 7 days while still in their lift-out form, and the FIB sharpening into APT needles was performed only after this exposure (Fig. S17). This sharpening step removed the most severely degraded outer surface, which is expected to be porous and mechanically compromised, and exposed material closer to the propagating degradation front. Therefore, the analyzed volume represents a partially degraded transition region rather than the fully decomposed surface layer.

This interpretation is supported by the APT reconstructions themselves. As shown in Fig. 4a, the air-exposed $Y_{2.1}Te_3$ specimen contains both O-rich degraded regions and chemically intact regions within the same reconstruction. The spatial separation between these regions indicates that the dataset captured the advancing reaction front. Such partially degraded material can still retain mechanical cohesion for field evaporation under conservative laser-pulsed APT measurement conditions (0.5% detection rate, ~60 pJ laser pulse energy, and a base temperature of 50 K). In contrast, fully decomposed regions are much more likely to fracture before useful data can be collected.

To clarify this point, we have added the following sentence to the Methods section (page 32):

"For the air-exposed APT specimens, FIB-cut lamellae mounted on Si posts were exposed to ambient air for 7 days while still in their lift-out form, and the FIB sharpening into APT needles was performed only after this exposure (Fig. S17). Although several of these tips fractured during APT analysis, representative datasets could be obtained because the analyzed volumes, exposed by the post-exposure FIB sharpening, corresponded to partially degraded reaction-front regions rather than fully disintegrated outer regions. These transitional regions retained sufficient mechanical integrity for field evaporation under the APT measurement conditions described above."
